# Identification of hydantoin based Decaprenylphosphoryl-β-d-Ribose Oxidase (DprE1) inhibitors as antimycobacterial agents using computational tools

**DOI:** 10.1038/s41598-022-20325-1

**Published:** 2022-09-30

**Authors:** Suraj N. Mali, Anima Pandey, Richie R. Bhandare, Afzal B. Shaik

**Affiliations:** 1 Department of Pharmaceutical Sciences and Technology, Birla Institute of Technology, Mesra, 835215 India; 2 Department of Pharmaceutical Sciences, College of Pharmacy and Health Sciences, Ajman University, P O Box 346, Ajman, United Arab Emirates; 3 Center of Medical and Bio-allied Health Sciences Research, Ajman University, P O Box 346, Ajman, United Arab Emirates; 4 St. Mary’s College of Pharmacy, St. Mary’s Group of Institutions Guntur, Affiliated to Jawaharlal Nehru Technological University Kakinada, Chebrolu, Guntur, Andhra Pradesh 522212 India

**Keywords:** Computational biology and bioinformatics, Drug discovery, Microbiology, Diseases

## Abstract

Tuberculosis (TB) is one of the emerging infectious diseases in the world. DprE1 (Decaprenylphosphoryl-β-d-ribose 2′-epimerase), an enzyme accountable for mycobacterial cell wall synthesis was the first drug gable target based on discoveries of inhibitors via HTS (high throughput screening). Since then, many literature reports have been published so far enlightening varieties of chemical scaffolds acting as inhibitors of DprE1. Herein, in our present study, we have developed statistically robust GA-MLR (genetic algorithm multiple linear regression), atom-based as well as field based-3D-QSAR models. Both atom-based as well as field based-3D-QSAR models (internally as well as externally validated) were obtained with robust Training set, R^2^ > 0.69 and Test set, Q^2^ > 0.50. We have also developed top ranked 5 point hypothesis AAAHR_1 among 14 CPHs (common pharmacophore hypotheses). We found that our dataset molecule had more docking score (XP mode = − 9.068 kcal/mol) than the standards isoniazid and ethambutol; when docked into binding pockets of enzyme 4P8C with Glide module. We further queried our best docked dataset molecule 151 for ligand based virtual screening using “*SwissSimilarity*” platform. Among 9 identified hits, we found ZINC12196803 had best binding energies and docking score (docking score = − 9.437 kcal/mol, MMGBSA dgBind = − 70.508 kcal/mol). Finally, our molecular dynamics studies for 1.2–100 ns depicts that these complexes are stable. We have also carried out in-silico ADMET predictions, Cardiac toxicity, ‘SwissTargetPredictions’ and Molecular Mechanics/Generalized Born Surface Area (MM/GBSA) binding energy calculations for further explorations of dataset as well as hit molecules. Our current studies showed that the hit molecule ZINC12196803 may enlighten the path for future developments of DprE1 inhibitors.

## Introduction

TB (tuberculosis), an infectious disease is responsible for deaths of 1.5 million people every year throughout the world. TB is caused by pathogenic bacteria called “*Mycobacterium tuberculosis*”^1^. Despite being a preventable infectious disease, millions of people die every year^2,3^. Most of these cases have been seen from low- and middle-income countries, but it has a profound presence throughout the world. TB is the main cause of HIV deaths and is being contributed to anti-Tb drug resistance^4,5^. WHO estimates the presence of one-quarter of the world’s population infected with TB^1^. As TB bacteria exist in the replicating and dormant forms, it becomes challenging to develop a novel anti-TB drug. Anti-Tb agents should act on both forms of the bacterium. Previously, we were just focusing on the developments of anti-TB drugs acting on the replicating forms, whilst it is also important to develop drugs acting and inhibiting the dormant forms of *Mtb.* Recently, there has been an emergence of MDR-TB and XDR-TB cases^6–20^.

**Figure1:**
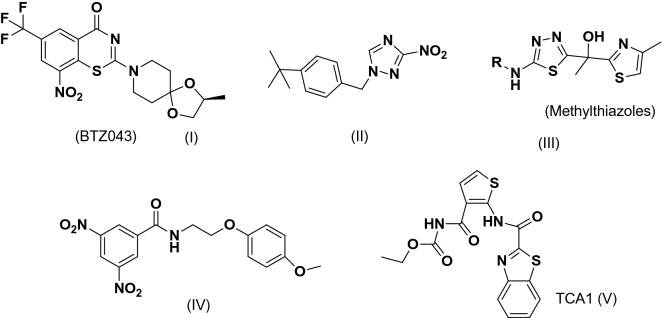
Literature reported compounds targeting DprE1inhibitors.

The research carried out by Christophe et al. and Makarov et al.^2^ depicted significance of decaprenylphosphoryl-β-d-ribose 2′-epimerase (DprE1) as a new potential anti-TB target for active drug molecules against *Mtb*. Decaprenylphosporyl arabinose (DPA) plays a crucial role by acting itself as a substrate for the arabinosyltransferase^12^. This is a key step responsible for *Mtb* cell wall synthesis i.e. arabinogalactan and lipoarabinomannan. DprE1 is a flavoprotein responsible for the oxidation of DPR (decaprenylphosphoryl-d-ribose) to DPX (decaprenylphosphoryl-2-ketoribose), which further reported to reduce by the enzyme DprE2 to DPA. A recent review explains thoroughly the broad classifications of DprE1 inhibitors based on the modes of bindings with the enzyme as covalent and non-covalent DprE1 inhibitors^12,13^. In one of the study, it was found that 2-carboxyquinoxalines (non-covalent inhibitors) possess an essential 2-carboxylate moiety essential for formations of key hydrogen bonds with the side-chain of Lysine 418 and the hydroxyl group of Tyrosine 60^14,15^. There have been several reports for a variety of the chemical scaffolds acting as DprE1 inhibitors. This list includes but not limited to scaffolds like dinitrobenzamides, azaindoles, pyrazolopyridines, benzothiazinones, etc. Recently, benzothiazinones BTZ043, PBTZ-169/macozinone and the azaindole AZ7371 have been entered in clinical trials as inhibitors of DprE1^13–20^. Some of the important inhibitors of DprE1 have been displayed in (Fig. 1). Nowadays, CADD (computer aided-drug designing) techniques are finding their ways in successful drug discoveries^20–43^.

In our present study, we have explored hydantoin as a new class of DprE1 inhibitors using atom based- as well as field-based-3D-QSAR studies. Thorough molecular modelling studies were carried out to generate pharmacophore hypothesis, and to identify new possible hits using ligand-based virtual screening, docking simulations, and molecular dynamics (online Supplementary, Figs. S1–S15). We had also extended our study to identify ADMET properties of the dataset as well as for new hits using a variety of commercial (QikProp, *V. 6.6*, Schrodinger, LLC, NY, 2020) as well as non-commercial software tools (admetSAR, SwissADME, pred-hERG, etc.). Our generated hits might pave the new way towards the development of DprE1 inhibitors.

## Materials and methods

### Dataset used for the developments of pharmacophore, QSARINS (QSAR-INSUBRIA) and 3D-QSAR models

For the developments of pharmacophore and 3D-QSAR models, we have used previously reported dataset of 100 compounds (online Supplementary Table S1). This dataset has diversity among pharmacological and structural characteristics^13^. For conversion of the IC_50_ (µM) values to pIC_50_, we used well known conversion formula i.e. (pIC_50_ =  − log10 IC_50_). DprE1 pIC_50_ is the negative logarithm of the IC_50_-concentration expressed in molar (M) obtained in the DprE1-inhibition assay. All pIC_50_ values were further considered for modelling studies^21^. The same dataset was used for the developments of GA-MLR (genetic algorithm multiple linear regression) models using popular software QSARINS ver. 2.2.2 and validated both internally and externally^43–45^.

### Software and hardware used

For our current study, we have utilized commercial Schrodinger’s small molecules drug discovery package installed on Linux operating system (Intel Pentium, i7 processor, 16 Gb RAM) (release 2020_4, Schrödinger, LLC, NY). For molecular docking analysis, we used Glide module incorporated in Schrödinger, LLC, NY package, 2020, V 8.9, (Maestro version 12.6). Studies pertaining to molecular dynamics, ADMET predictions, pharmacophore developments and binding energy calculations (MMGBSA) were carried out using desmond (V.6.4), Qikprop (V 6.6), Phase and Prime modules respectively (release 2020, Schrödinger, LLC, NY)^21,24–26,29,36^. Further, we have also executed popular non-commercial (free) online software tools like SwissADME, SwissSimilarity, SwissTarget prediction, lazar toxicity predictions and admetSAR for our current studies^22,30–32^. The higher resolution images pertaining to 3D-QSAR, docking, MMGBSA and CPH (common pharmacophore hypothesis) were generated through Schrödinger’s molecular modelling software and Gimp freeware. The detailed analysis of descriptors generation, pruning of descriptors, and QSAR validations using the QSARINS ver. 2.2.2 has been enclosed in *the supporting information*^43–45^.

### Retrieval of the 3D crystal structure of the *M. tuberculosis* DprE1 complex

**Figure 2:**
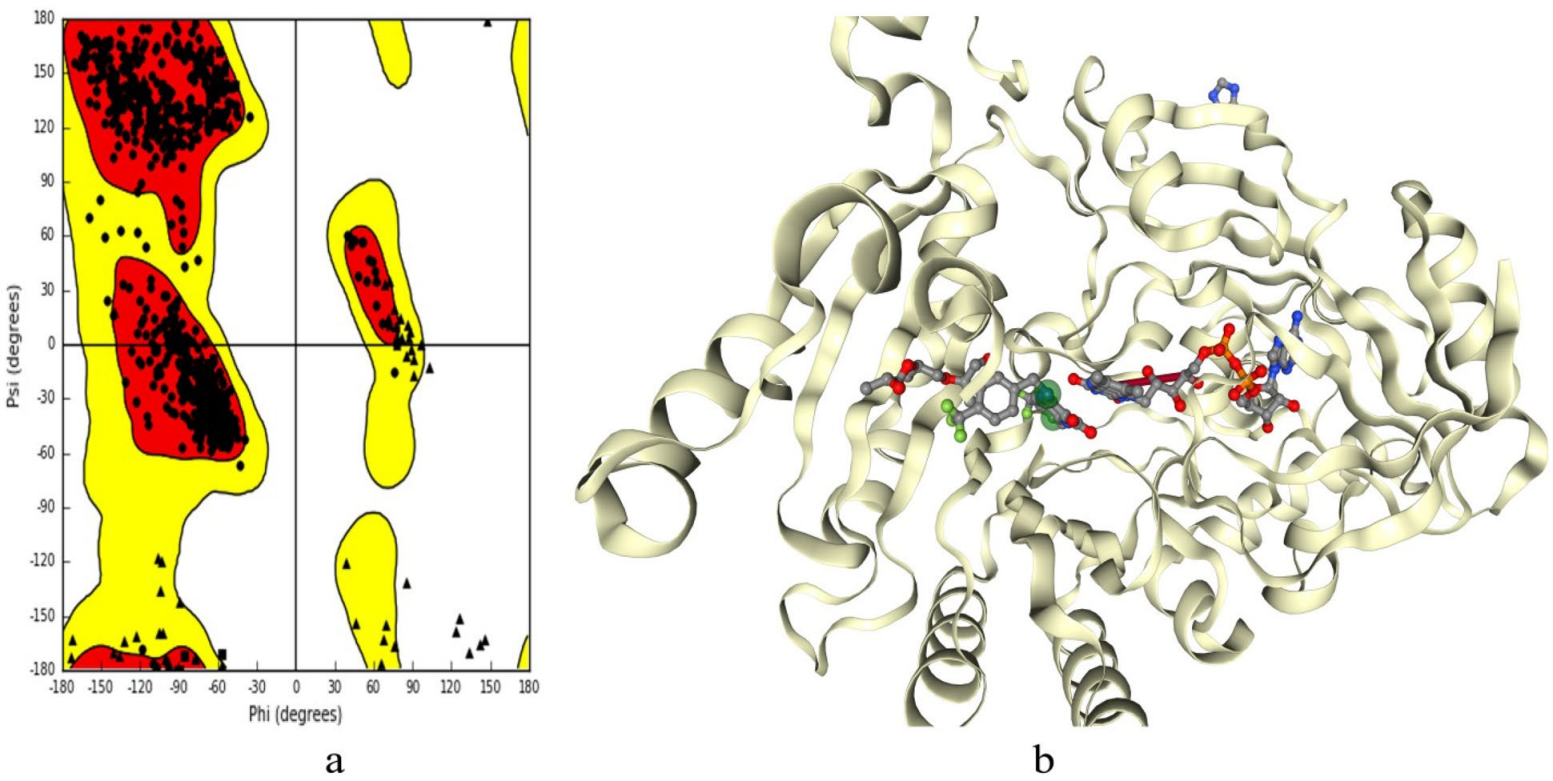
(**a**) Ramchandran plot and (**b**) binding pocket for native ligands for protein 4P8C [(**b**) this figure has been visualized with ‘BIOVIA Discovery Studio Visualizer’ V.2022, available at: https://discover.3ds.com/discovery-studio-visualizer-download].

We have carefully selected and collected the necessary crystal structure of the M. tuberculosis DprE1 complex (pdb id:4P8C, Resolution: 1.95 Å) from the Protein Data Bank (http://www.rcsb.org). The selection of pdb id was performed based on our careful literature studies. This enzyme (monomer) essentially consisting of 2 chains, chain A (448 residues) and chain B (417 amino acid residues). Figure 2a, depicts necessary angles ψ against φ of amino acid residues characteristics to be visualized from Ramachandran plot for given protein structure **4P8C**. The favoured region and generously allowed regions are generally depicted by red and yellow regions of Ramachandran plot respectively. The key residues involved in binding of native ligands were taken into considerations for molecular docking simulations, which generally gives idea for binding pockets of enzyme (Fig. 2b).

### Protein preparation for selected target *Mycobacterium tuberculosis* DprE1

The required 3D crystal structure of ***M. tuberculosis DprE1*** was imported into maestro workflow (http://www.rcsb.org). We processed the protein so that it was free from all the cofactors, inbound ligands and water molecules. Schrödinger’s protein preparation wizard was used for adding missing residues, adding hydrogens, generating Het states, and optimisation of the selected protein. After, processing of required protein, it was further processed for grid generation. Default parameter like centroid of inbound co-crystal ligand was used for making the grid. OPLS-2005 (optimized potentials for liquid simulations) force field was utilized throughout the entire protein preparations and simulations^36,37^.

### Preparations of ligands for study

Firstly, we have sketched all the ligand structures selected from the literature source^13^ (tabulated in SI) in popular ‘ChemBiodraw’ (Version 12.0.2) in 2D forms and further saved in .sdf or .mol file format. These 2D structures then allowed importing into LigPrep module (Schrödinger, LLC, NY,2020) incorporated in maestro (V 12.6, 2020) for successful conversion into 3D forms. LigPrep utility was known to provide least energy conformers via ConfGen. After conversion into 3D forms, we allowed to run for geometrical optimization, add hydrogen, and generate a stereoisomer. The default option of generating 32 conformers for ligand was kept intact. The ‘Epik’ module (V 5.4, Schrödinger, LLC, NY 2020) was used for generating possible ionisation states for each ligand at pH = 7.0 ± 0.0. For our entire ligand preparation protocols, we adhered to OPLS-2005 force field^28^.

### Common pharmacophore modelling (virtual screening approach)

**Figure 3:**
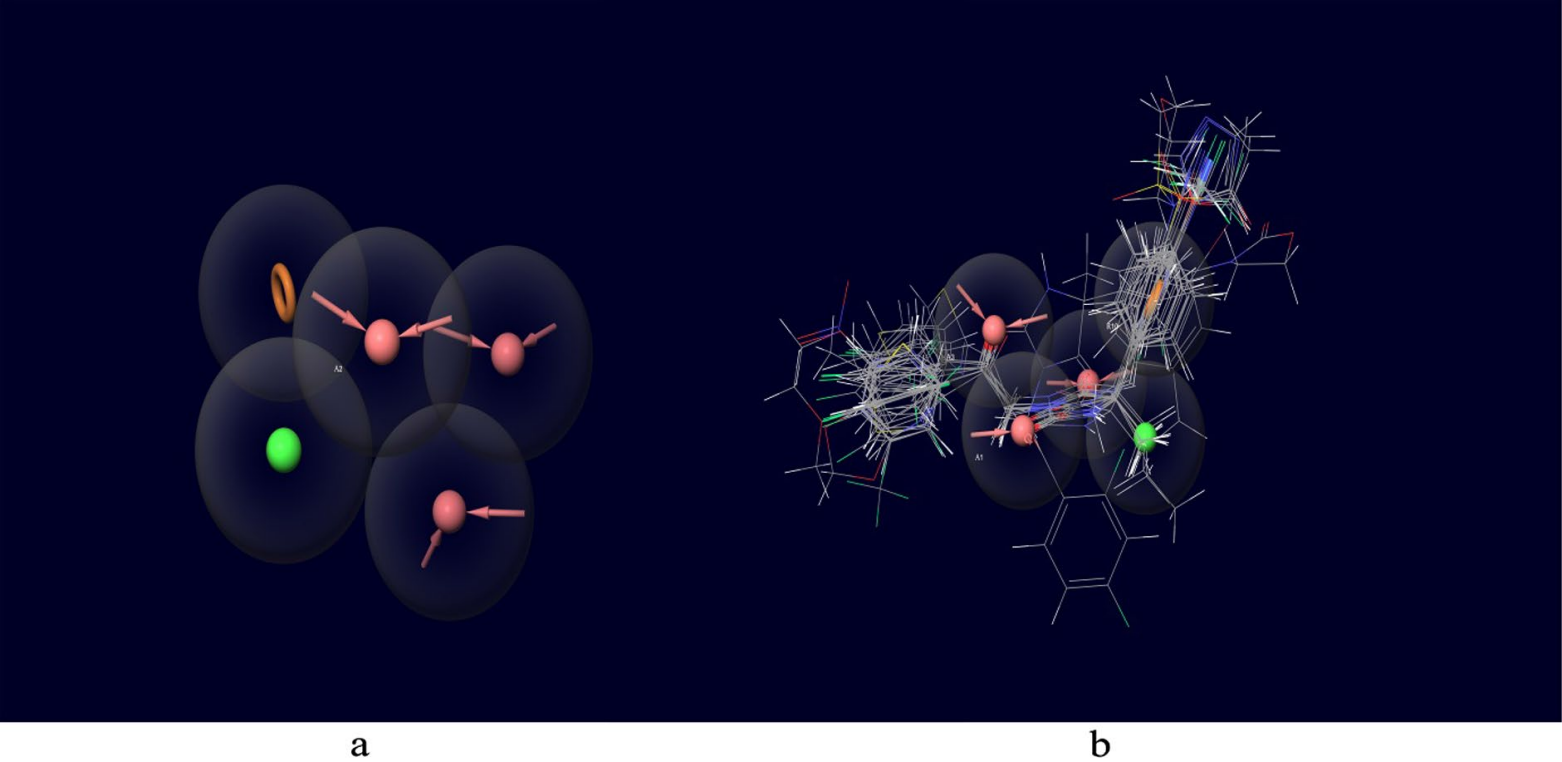
(**a**) A five point Pharmacophore model (AAAHR_1) generated by PHASE. The model illustrates acceptor feature (AAA; red coloured arrows), Hydrophobic feature (H:green) and aromatic ring (R: Brown color) features. (**b**) All active ligands overlapped on the generated model AAAHR_1 (this figure has been visualized from ‘Phase module’ Schrodinger, LLC, NY, V.2020; Available at: https://www.schrodinger.com/).

We have applied “Phase” module (Schrodinger, 2020) for efficient developments of common pharmacophore hypothesis (CPH) (Table 1)^21^. There are number of literature reports for usage of Phase for CPH generations and 3D-QSAR models. With the help of flexible ligand alignments, firstly we aligned all 100 ‘Ligprep’ molecules. Use of the macromodel search method with maximum of 1000 per structure was applied in order to generate conformers for all the ligands. They were further minimized using the OPLS-2005 force field, as it has 100 steps of minimization. Finally, we have generated 14 CPH models, out of them we selected best top-ranked CPH (AAAHR_1) based on its scores and utilized for 3D-QSAR generations. We developed 5 point hypothesis as AAAHR_1. All actives were allowed to align on AAAHR_1 (Fig. 3). This CPH has 3 hydrogen bond acceptor (A), 1 hydrophobic group (H), and 1 aromatic ring (R) features as depicted by Phase utility. We have shortlisted ligands into 3 categories as active, inactive and intermediates based on biological data. The splitting pattern was considered by referring previous publications. Ligands with activity above 5.50 (pIC50 > 5.50) were considered as actives, while those having below 4.50 (pIC50 < 4.50) considered as inactive. While in between actives and in-actives there were intermediates (5.50 > Intermediates > 4.50). So, in our CPH developments there were 54 actives, 33 inactive and 13 intermediates (total of 100 ligands).

**Table 1:** Various pharmacophore hypotheses generated by PHASE.

Sr. no	Hypothesis	Survival scores	Site score	Vector score	Volume score	Selectivity	Inactive score	Phase hypo score
1	**AAAHR_1**	**5.931836**	**0.895113**	**0.962592**	**0.81223**	**1.529507**	**2.309382**	**1.36**
2	AAAHR_3	5.901082	0.891958	0.968926	0.796781	1.511023	2.319135	1.35
3	AAHRR_1	6.045822	0.844886	0.9061	0.815984	1.754577	2.244988	1.35
4	AAAHR_2	5.90853	0.873433	0.973764	0.798487	1.530452	2.278402	1.35
5	AAHRR_2	6.023762	0.876879	0.855126	0.820522	1.746959	2.021338	1.35
6	AAHRR_4	6.018948	0.881016	0.875326	0.809973	1.728357	2.336682	1.35
7	AAHRR_3	6.019396	0.849673	0.92015	0.787902	1.737394	2.266527	1.35
8	AAHRR_5	5.992291	0.807977	0.902254	0.81122	1.746563	2.05645	1.35
9	AAHRR_6	5.944514	0.773089	0.894152	0.780675	1.772322	2.045571	1.35
10	AAHRR_7	5.933289	0.782156	0.911423	0.793733	1.721702	2.240607	1.34
11	AAHR_3	5.652884	0.907227	0.956151	0.812921	1.244191	2.231216	1.34
12	AAHR_1	5.672322	0.908235	0.956427	0.81333	1.261937	2.188775	1.33
13	AAHR_2	5.660586	0.936981	0.973037	0.785876	1.232298	2.566902	1.33
14	AHRR_1	5.772924	0.916642	0.884065	0.833694	1.414248	2.142401	1.33

Significant values are given in bold.

### Scoring for best pharmacophore hypothesis

We have developed 14 pharmacophore hypotheses; which were further ranked according to their scores. The best hypothesis was selected on basis of survival score, site score, vector score, etc. (Table 1).

### Atom-based and field-based 3D-QSAR modelling using the flexible ligand alignment

**Figure 4:**
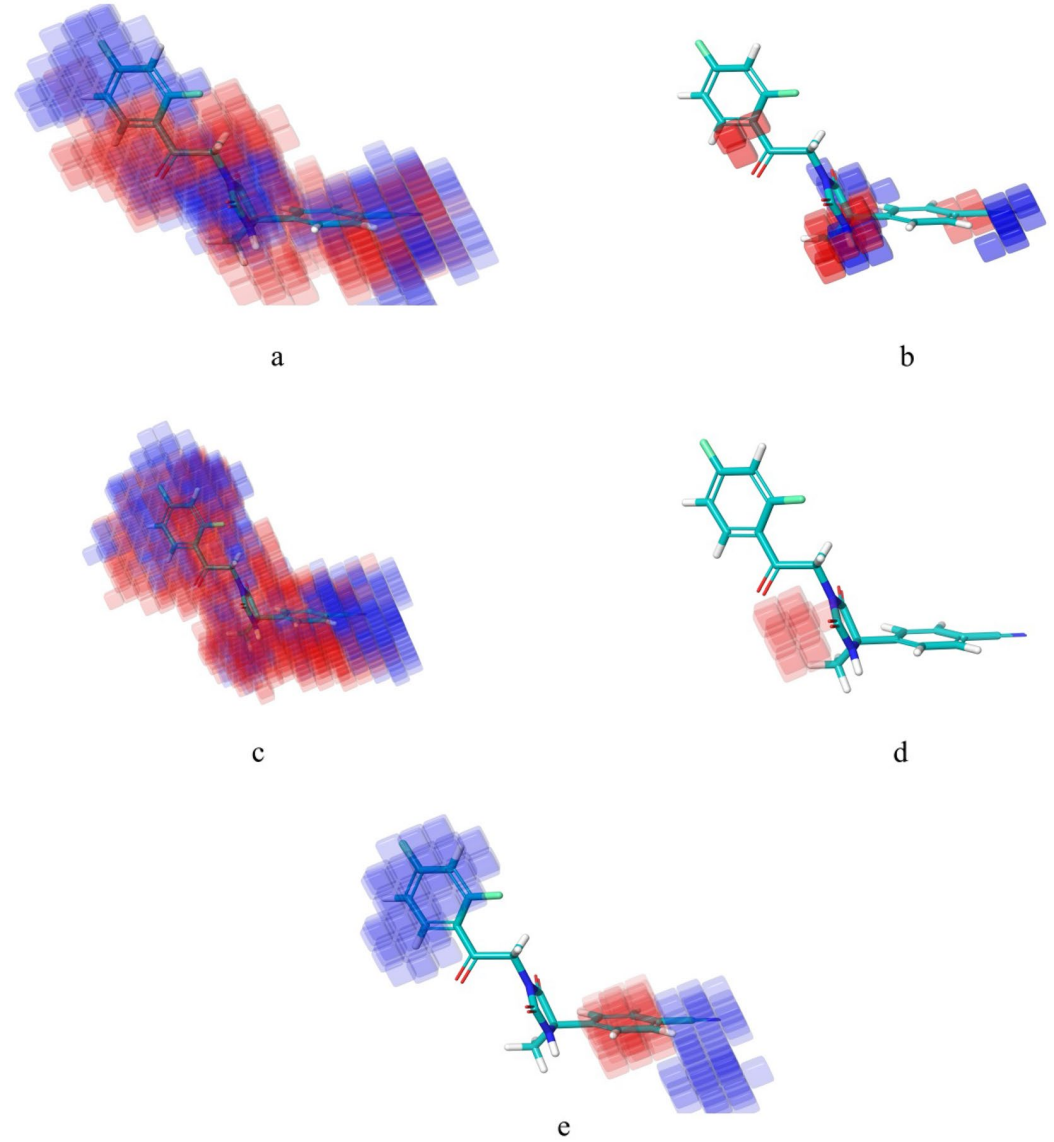
(**a**–**e**) Visual representation of atom-based PHASE 3D-QSAR model(compound 1)—(**a**) electron withdrawing, (**b**) hydrogen bond donor, (**c**) hydrophobic, (**d**) negative ionic and (**e**) others. Blue color cubes indicate positive coefficient or increase in activity and red colour cubes indicate negative coefficient or decrease in activity (this figure has been visualized from ‘Phase module’ Schrodinger, LLC, NY, V.2020, Available at: https://www.schrodinger.com/).

**Figure 5:**
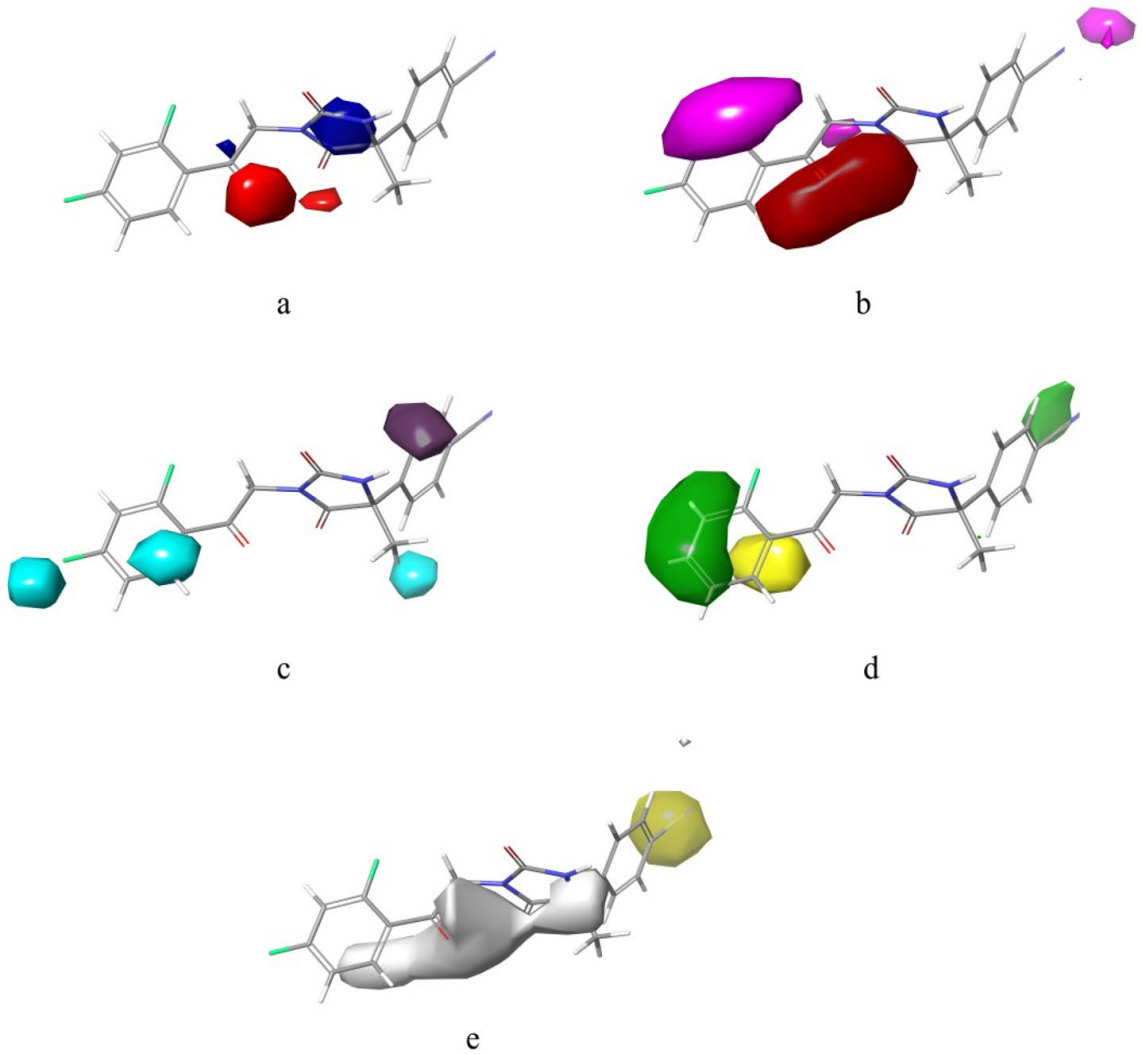
Fields contour maps based on test set compounds. (**a**) Gaussian electrostatic fields: favored electropositive (blue) and disfavored electronegative (red). (**b**) Gaussian hydrogen bond acceptor field: favored (red) and disfavored (magenta). (**c**) Gaussian Hydrogen bond donor field: favored (purple) and disfavored (cyan). (**d**) Gaussian Steric field: favored (green) unfavorable (yellow). (**e**) Gaussian Hydrophobic field: favoured (yellow) and disfavored (white) (this figure has been visualized from ‘Phase module’ Schrodinger, LLC, NY, V.2020, Available at: https://www.schrodinger.com/].

Phase module from maestro (V 12.6) interface of Schrodinger's utility was utilized for developments of 3D-QSAR models. For better understanding of correlation between structural features and biological activity, we tend to develop atom based as well as field based-3D-QSAR models (online Supplementary, Fig. S1). All the models were developed through random selection of training set and test set into 70%:30% by using default settings of ‘Phase’ module as per literature procedures defined earlier and adopted widely^36,38–41^. However, we ensured that developed models were not a chance of randomness and further assessed for their internal and external validations for statistical significance. Further splitting of datasets into training and test sets, were further checked for diverse chemical space adopted by molecules^36,38–41^. Active as well as inactive molecules were part of both sets to ensure good reliability of developed models. Further, for MLR based QSAR models, same strategy was used. In all cases, we have thoroughly checked our models for their robustness as per statistical formulas mentioned in the Supplementary information^36,38–41^. We randomly divided the dataset into 70% training set and 30% test set molecules with application of PLS factor of 4 for both 3D-QSAR models. The random selection made by software was further checked by visualizing the training and test set molecules (diversity among dataset molecules in training and test sets). We have taken care to maintain 1 Å grid spacing for the selected hypothesis. We have developed 4 atom-based 3D-QSAR models, while in case of filed based we developed 3 models (Tables 2, 3, 4, 5, 6, 7). In case of Atom-based models, we have incorporated 68 molecules in training set and 32 molecules in test set (Table 4). However, in case of field-based models there were 70 training set and 30 test set molecules. Statistical parameters were applied to select best models (Table 5) (online Supplementary Table S2). The Gaussian field-based 3D-QSAR models were consisting of Gaussian steric, electrostatic, hydrophobic, hydrogen bond donor, and hydrogen bond acceptor. In field-based models, we considered Gaussian intensities as descriptors (as independent variables). Finally, best selected 3D-QSAR models were developed to visualize for the 3D contour maps associated with structural features (Figs. 4, 5). QSAR model visualization has its own importance for better optimization of the scaffolds.

**Table 2:** PLS parameters for developed Atom based 3D-QSAR models.

Statistical parameters	PLS model (PLS factor 4)			
	PLS factor 1	PLS factor 2	PLS factor 3	PLS factor 4
Number of molecules in the training set	68	68	68	68
Number of molecules in the test set	32	32	32	32
**Regression coefficient**				
Training set, R^2^	0.4053	0.5826	0.7815	0.8838
Test set, Q^2^	0.1976	0.4695	0.4844	0.5054
Standard deviation (SD)	0.926	0.7818	0.5701	0.419
Root mean square error (RMSE)	1.1	0.89	0.88	0.86
Stability	0.904	0.753	0.364	0.253
Pearson correlation coefficient (Pearson-r)	0.4585	0.6909	0.7088	0.725
Variance ratio (F-value)	45	45.4	76.3	119.7
Significance level of variance ratio (P-value)	5.37E−09	4.66E−13	4.20E−21	1.04E−28

**Table 3:** The atom based 3D-QSAR statistics for atom type fraction.

# Factors	H-bond donor	Hydrophobic/non-polar	Negative ionic	Electron-withdrawing	Other
1	0.02	0.631	0.01	0.288	0.051
2	0.021	0.611	0.006	0.291	0.071
3	0.022	0.597	0.001	0.286	0.093
4	0.022	0.598	0.001	0.286	0.094

**Table 4:** Experimental dataset^a^ employed for Atom based 3D-QSAR study along with docking scores and predicted/actual pIC_50_ (µM) values (against) (PLS Factor-4).

Ligand name	QSAR set	Actual activity	Predicted activity	Docking score (xp) kcal/mol (pdb id: 4P8C)
1	Training	7	6.71122	− 7.238
4	Training	4.3	5.80306	− 6.117
7	Training	6.7	7.10433	− 7.836
24	Training	6.7	5.80306	− 6.117
25	Test	6.7	6.46505	− 6.756
26	Training	6	5.65203	− 7.01
27	Test	6	5.82176	− 6.284
28	Training	4.2	5.33654	− 5.817
29	Test	< 4	4.97518	− 6.513
30	Test	< 4	4.54298	− 6.847
31	Training	< 4	4.44724	− 6.684
32	Test	< 4	4.21836	− 6.843
33	Training	< 4	3.64479	− 6.251
34	Test	< 4	4.03335	− 6.754
35	Training	4.3	4.41229	− 7.305
36	Training	4.4	4.17767	− 6.705
37	Training	< 4	3.86292	− 6.933
38	Training	5.4	5.51548	− 6.365
39	Test	4.6	5.32639	− 6.594
40	Test	4.4	4.21767	− 6.514
41	Training	5	4.66835	− 6.622
42	Training	< 4	4.06428	− 7.79
43A	Test	4	4.54857	− 7.687
43B	Training	4.2	4.54857	− 7.687
44	Test	4.2	5.68154	− 6.946
46	Training	< 4	3.88324	− 6.574
47	Training	< 4	4.06756	− 3.963
109	Training	7.1	6.64915	− 7.054
110	Training	6.1	6.58056	− 6.519
111	Test	7	6.99898	− 7.143
112	Training	6.9	6.50982	− 7.629
113	Training	7.3	7.23067	− 7.223
114	Test	7.4	5.83347	− 7.341
115	Test	6.5	6.55501	− 7.595
116	Test	7.3	5.54471	− 6.965
117	Test	7.1	6.11971	− 8.346
118	Training	6.6	6.35222	− 8.834
119	Training	5.6	6.07747	− 7.904
120	Training	6.8	6.89516	− 6.861
121	Training	4.4	4.58261	− 6.905
122	Test	< 4	4.74593	− 7.415
123	Training	4.5	4.98555	− 7.65
124	Test	5.7	6.91001	− 5.621
125	Training	6.5	6.39954	− 6.334
126	Training	6.7	6.4211	− 6.713
127	Training	6.9	6.97828	− 5.682
128	Training	< 4	3.549	− 6.512
129	Training	4.4	3.90246	− 6.992
130	Test	6.4	6.21595	− 6.941
131	Training	7.3	7.01925	− 6.966
132	Training	< 4	4.54339	− 7.083
133	Training	6.9	7.12085	− 6.897
134	Training	7.3	7.10961	− 7.862
135	Training	6.2	6.94287	− 6.858
136	Training	4.7	4.43187	− 7.492
137	Test	5.1	5.97086	− 8.354
138	Test	6.7	5.57084	− 6.194
139	Training	7	6.46065	− 7.932
140	Training	4.2	3.92229	− 7.097
141	Test	4.1	4.59635	− 7.758
142	Training	6	6.55797	− 6.779
143	Test	6.6	6.77677	− 6.987
149	Training	5.8	5.26483	− 5.503
151	Training	< 4	4.01458	− 9.068
155	Training	5.1	5.28465	− 6.982
156	Test	4.9	5.12907	− 6.462
157	Training	4.5	4.7725	− 6.158
160	Training	4.7	4.54468	− 6.271
161	Training	< 4	3.8964	− 6.602
163	Training	< 4	4.34517	− 6.056
180	Test	7.2	6.60346	− 6.723
181	Training	6.4	6.43155	− 5.107
182	Training	7.3	6.84464	− 6.489
183	Training	7.2	6.82754	− 6.761
184	Training	5.7	5.67255	− 6.809
185	Test	6.2	6.14353	− 5.899
186	Training	5.9	5.41442	− 6.545
187	Training	4.4	4.57332	− 6.458
188	Training	6.5	6.44496	− 6.571
189	Test	5.6	5.93	− 6.639
190	Training	6.3	6.56541	− 6.952
191	Training	5.7	5.76688	− 7.494
192	Training	6.7	6.24905	− 6.912
193	Test	7	6.0381	− 6.939
194	Test	6.8	6.35491	− 6.87
195	Training	5.7	5.97326	− 5.716
196	Training	6.7	6.59133	− 6.046
197	Test	5.7	5.936	− 6.577
198	Test	4.7	5.81499	− 5.668
199	Training	5.3	5.09867	− 6.331
200	Training	4.6	5.06595	− 6.339
201	Training	6.6	6.15047	− 6.596
202	Training	6.4	6.29976	− 6.61
203	Training	5.4	5.77936	− 7.34
204	Training	7	6.90883	− 7.227
205	Training	5	5.07395	− 7.003
206	Training	7	6.89124	− 5.151
207	Training	7.2	7.02906	− 6.485
208	Test	4.3	5.73972	− 7.181
209	Test	4.4	6.27557	− 6.083

^a^DprE1 pIC_50_ is the negative logarithm of the IC_50-_concentration expressed in molar (M) obtained in the DprE1-inhibition assay; values < 4 was treated as **≈** 4.0 for sake of QSAR developments.

**Table 5:** Experimental dataset^a^ employed for Field based 3D-QSAR study along with predicted/ actual pIC_50_ (µM) values (against) (PLS factor-3).

Ligand name	QSAR set	Actual activity	Predicted activity
1	Training	7	6.10082
4	Training	4.3	5.66211
7	Training	6.7	6.11888
24	Training	6.7	5.66211
25	Training	6.7	5.59165
26	Training	6	5.68028
27	Training	6	5.50716
28	Test	4.2	5.81369
29	Training	< 4	4.71645
30	Training	< 4	4.82689
31	Test	< 4	4.95305
32	Training	< 4	3.79922
33	Training	< 4	3.49725
34	Test	< 4	3.74747
35	Test	4.3	4.4435
36	Training	4.4	4.01246
37	Training	< 4	4.2946
38	Test	5.4	5.77377
39	Training	4.6	4.94864
40	Test	4.4	4.66993
41	Training	5	5.05224
42	Test	< 4	5.69583
43A	Training	4	4.55733
43B	Training	4.2	4.55733
44	Training	4.2	5.02306
46	Training	< 4	3.33966
47	Test	< 4	3.20449
109	Test	7.1	5.72532
110	Test	6.1	5.60888
111	Test	7	5.86738
112	Test	6.9	7.26893
113	Training	7.3	8.15042
114	Training	7.4	6.96995
115	Training	6.5	7.10337
116	Test	7.3	7.04264
117	Training	7.1	6.70553
118	Training	6.6	6.76209
119	Test	5.6	5.86526
120	Training	6.8	6.4697
121	Training	4.4	5.1949
122	Training	< 4	5.27201
123	Test	4.5	4.83226
124	Training	5.7	5.55523
125	Training	6.5	5.67364
126	Test	6.7	5.4366
127	Training	6.9	6.35027
128	Training	< 4	4.33974
129	Training	4.4	3.85812
130	Training	6.4	5.89607
131	Training	7.3	6.43156
132	Training	< 4	5.93841
133	Training	6.9	6.32088
134	Training	7.3	6.37863
135	Training	6.2	6.57981
136	Training	4.7	5.47606
137	Training	5.1	5.53093
138	Training	6.7	5.87643
139	Training	7	7.10182
140	Test	4.2	4.37926
141	Training	4.1	3.89458
142	Training	6	5.87527
143	Training	6.6	6.16728
149	Training	5.8	5.38632
151	Test	< 4	4.59198
155	Training	5.1	4.83155
156	Test	4.9	5.30381
157	Training	4.5	4.99565
160	Training	4.7	4.11717
161	Training	< 4	3.95283
163	Training	< 4	4.58974
180	Test	7.2	6.5701
181	Training	6.4	6.93056
182	Test	7.3	6.40699
183	Training	7.2	6.47744
184	Training	5.7	5.56804
185	Training	6.2	6.74523
186	Test	5.9	5.3185
187	Training	4.4	5.31995
188	Test	6.5	6.05776
189	Training	5.6	5.37319
190	Test	6.3	6.38775
191	Test	5.7	6.69532
192	Test	6.7	6.88175
193	Training	7	6.00603
194	Training	6.8	6.78083
195	Training	5.7	5.52751
196	Training	6.7	6.855
197	Training	5.7	5.56247
198	Training	4.7	4.3781
199	Training	5.3	5.798
200	Test	4.6	5.90879
201	Test	6.6	6.42449
202	Test	6.4	6.84693
203	Training	5.4	6.5968
204	Training	7	6.16027
205	Test	5	6.76156
206	Training	7	6.83376
207	Training	7.2	6.41214
208	Training	4.3	5.29981
209	Training	4.4	5.1808

^a^DprE1 pIC_50_ is the negative logarithm of the IC_50-_concentration expressed in molar (M) obtained in the DprE1-inhibition assay; values < 4 were treated as **≈** 4.0 for sake of QSAR developments.

**Table 6:** PLS parameters for developed field based 3D-QSAR models.

Statistical parameters	PLS statistics		
	Factor 1	Factor 2	Factor 3
Number of molecules in the training set	70	70	70
Number of molecules in the test set	30	30	30
**Regression coefficient**			
Training set, R^2^	0.4135	0.5906	0.691
Test set, Q^2^	0.4354	0.5194	0.5085
Standard deviation (SD)	0.9316	0.7842	0.6865
Root mean square error (RMSE)	0.9	0.83	0.84
Stability	0.867	0.704	0.577
Pearson correlation coefficient (Pearson-r)	0.6683	0.7208	0.7267
Variance ratio (F-value)	47.9	48.3	49.2
Significance level of variance ratio (P-value)	1.94E−09	1.02E−13	8.13E−17

**Table 7:** The field based 3D-QSAR statistics for field fractions.

# Factors	Gaussian steric	Gaussian electrostatic	Gaussian hydrophobic	Gaussian Hbond acceptor	Gaussian Hbond donor
1	0.429	0.081	0.2	0.211	0.079
2	0.4	0.088	0.231	0.196	0.084
3	0.382	0.097	0.235	0.184	0.102

### Molecular docking simulations, target predictions, binding energy calculations and ligand based-virtual screenings

**Figure 6:**
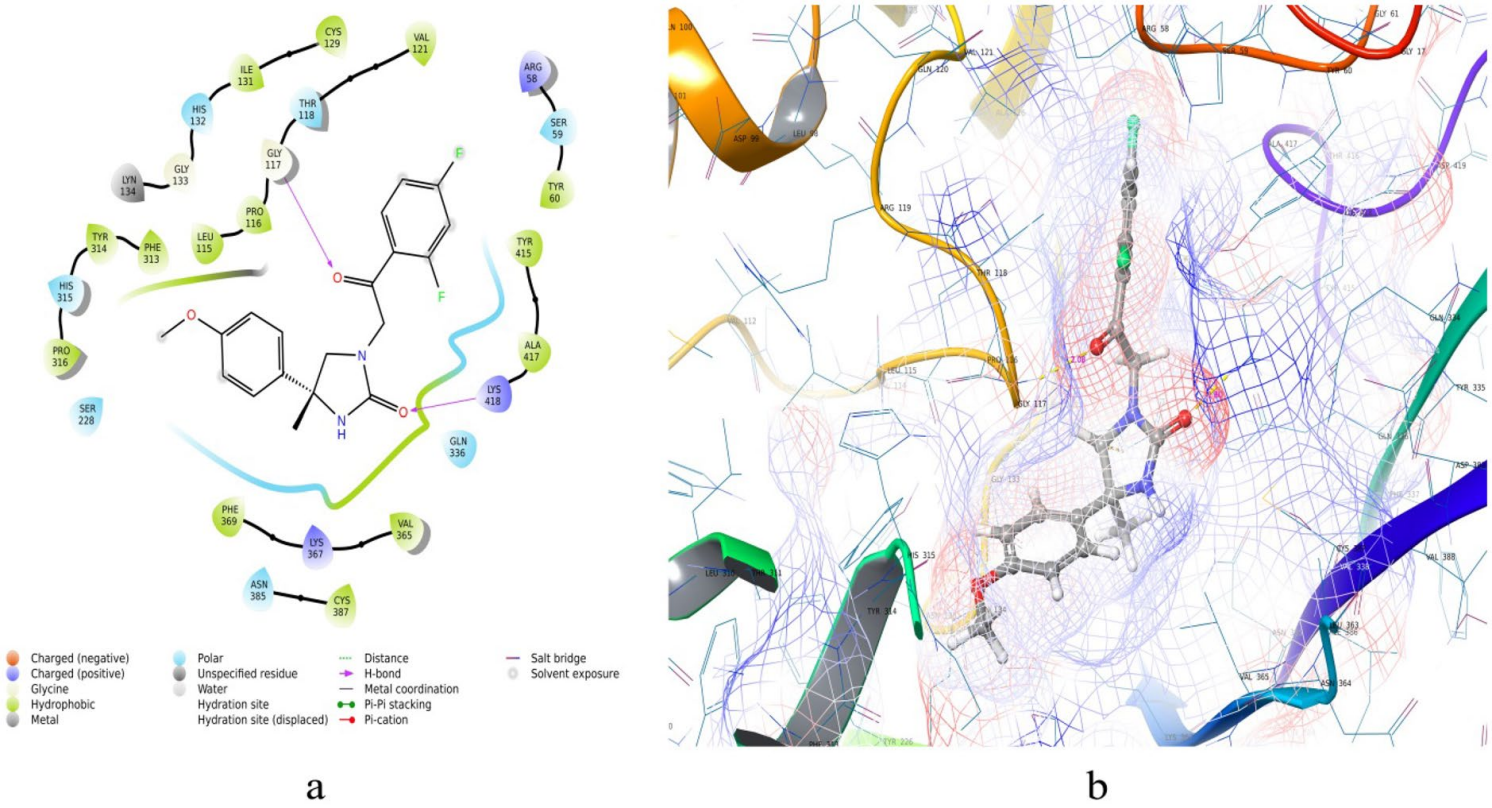
2D (**a**) and 3D (**b**) Ligand interaction diagram with the best docked molecule 151 (this figure has been visualized from ‘Glide module’ Schrodinger, LLC, NY, V.2020, Available at: https://www.schrodinger.com/).

**Figure 7:**
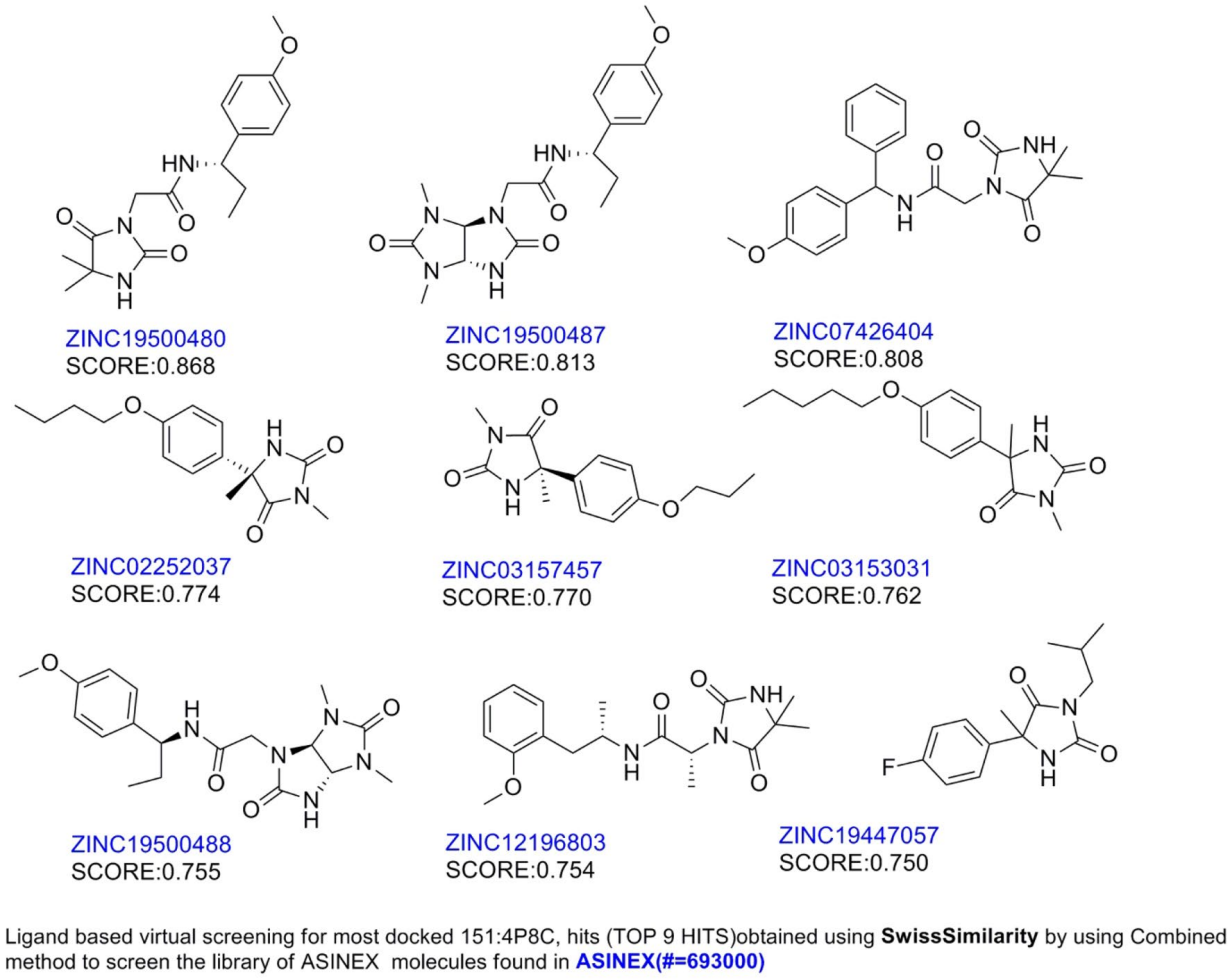
Structures of screened top 9 compound after virtual screening**.**

**Figure 8:**
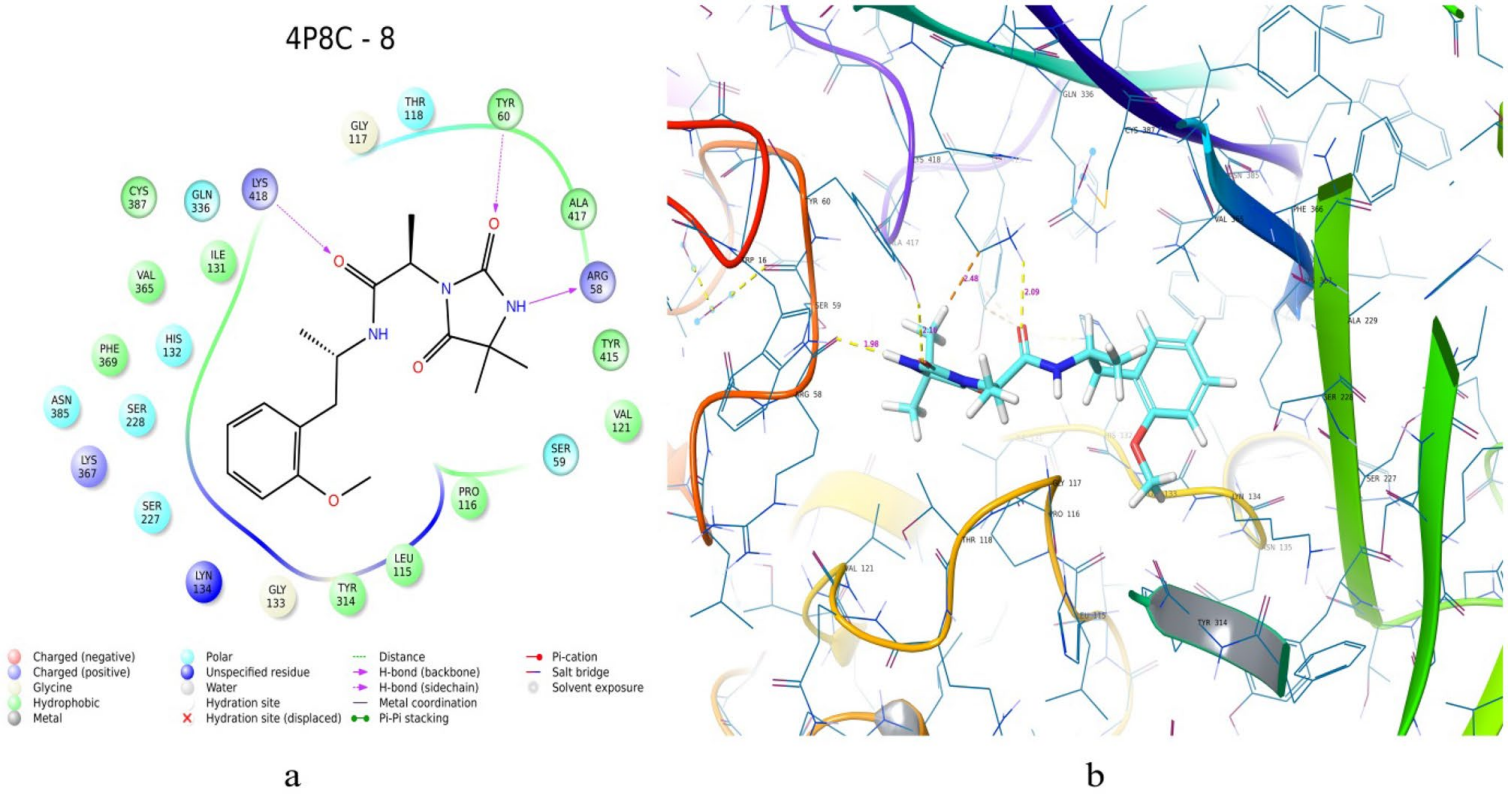
most docked ASINEX hit (XP docking): (**a**) 2D- and (**b**) 3D-representation of binding mode for ZINC12196803 (this figure has been visualized from ‘Glide module’ Schrodinger, LLC, NY, V.2020, Available at: https://www.schrodinger.com/).

**Figure 9:**
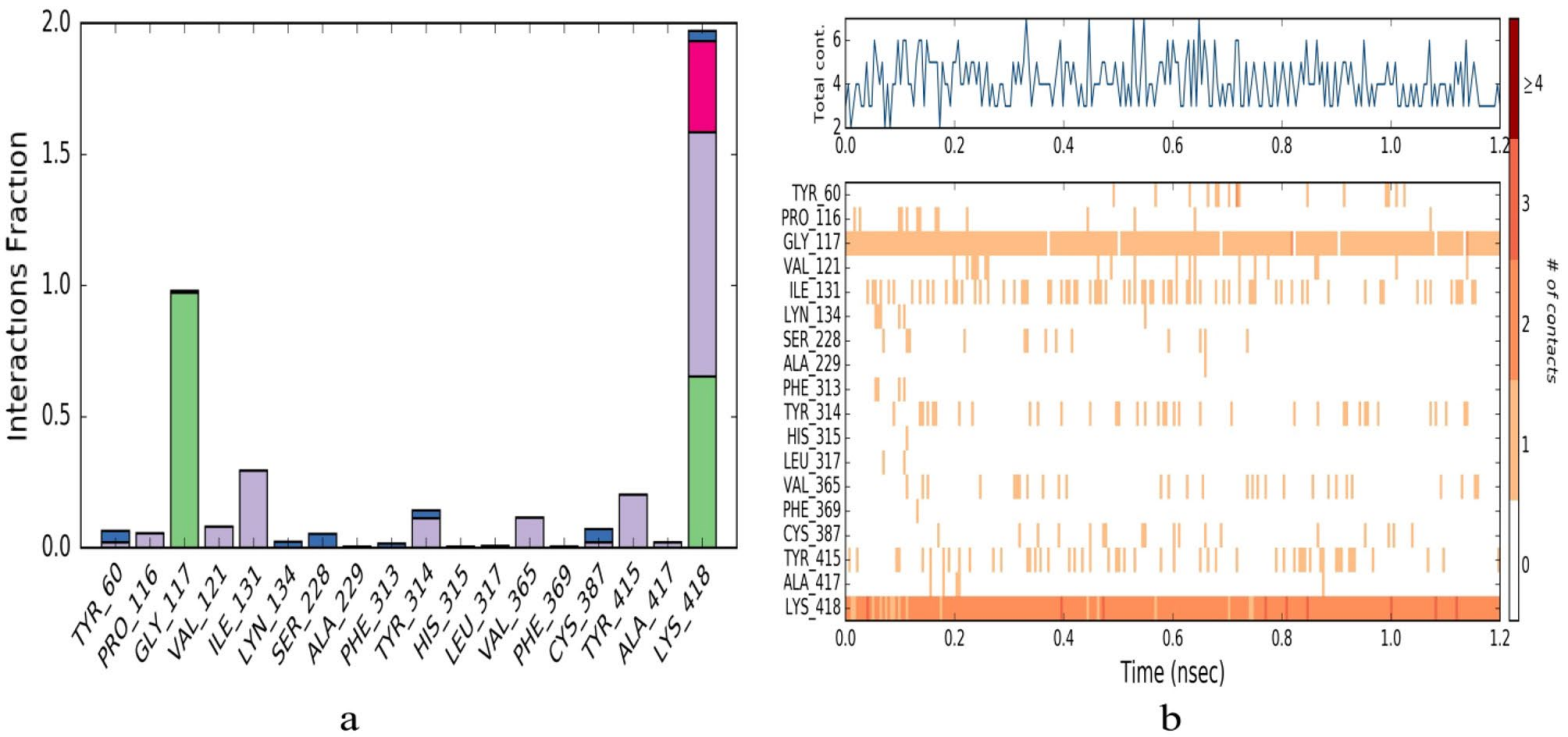
(**a**) Showing the protein ligand(4p8c:151) interaction throughout the simulation time of 1.2 ns (green-H-bond, purple-hydrophobic contacts, Pink-ionic contacts and Blue-Water bridges). Values more than 1 suggesting more contacts and corresponding to 100%. (**b**) A timeline representation of the interactions and contacts (H-bonds, Hydrophobic, Ionic, Water bridges).

**Figure 10:**
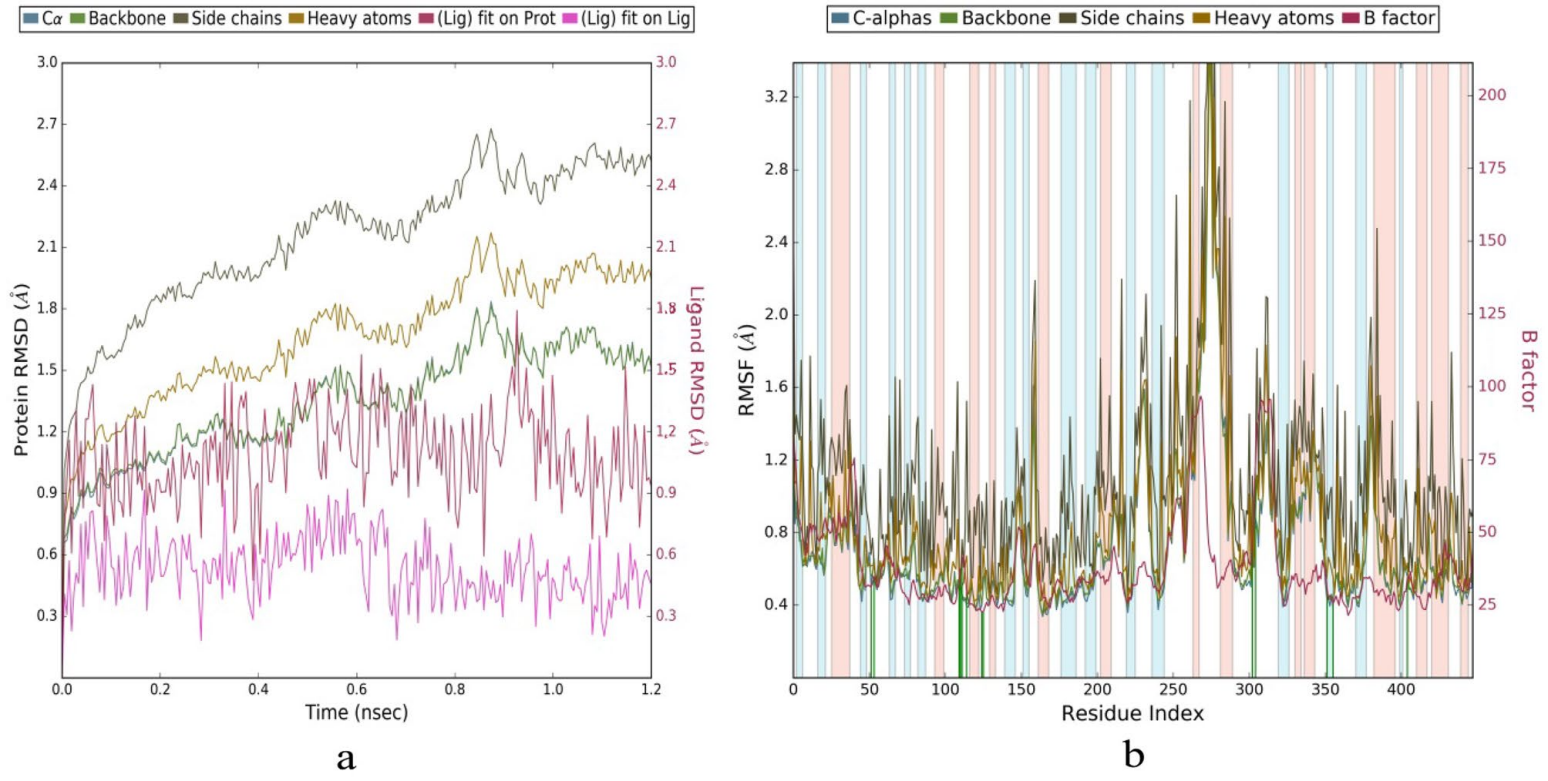
(**a**) RMSD Plot Showing the protein ligand interaction throughout the simulation time of 1.2 ns. (**b**) RMSF plot for the target protein selected (for 4P8C:ZINC12196803) [Simulation Graphs using GROMACS, has been attached in the SI, for 100 ns results].

All the 100 ligands were allowed to dock into active pocket of selected protein 4P8C^43^. We have employed Glide V 8.9 for molecular docking simulations (PDB ID: 4P8C) to analyse compound binding for dataset as well as hit molecules (Table 4) (online Supplementary Fig. S8). As DprE1 has two chains, chain A (448 residues) and chain B (417 residues). We had taken the precaution before docking that corresponding protein and ligands were processed from Protein preparation wizard and LigPrep respectively. We noticed that co-crystal ligand from protein DprE1 shows binding residues with its inbound ligand as His132, Gly133, Lys134, Leu317, Val365, Lys367, Cys387 and Lys418. So, it is essential to dock dataset molecules as well as hits in order to get correlation between docking results and ligands to serve as inhibitors of enzyme (Table 4). So, we prepared the crystal structures with the co-crystallized ligandY22 for docking simulations. After completion of docking, the best docked dataset molecule 151(Fig. 6) was employed further for predictions of molecular targets other than one which docked by using the free utility “SwissTargetPrediction”. Further, same molecule was allowed for ligand based-virtual screening of hits using the “SwissSimilarity” online tool (online Supplementary Figs. S4, S6; Figs. 7, 8). By using this utility, we screened almost ASINEX hits (# = 69,3000 molecules) using combined method as reported by search algorithm. After, screening of hits, top 9 hits were further visualized for their pharmacokinetics characteristics, water solubility, toxicity studies, etc. MDS studies were also performed for 151 as well as best ASINEX hit molecule (Desmond 6.4, release 2020) (Figs. 9 and 10). We employed 3 docking modes HTVS (High throughput virtual screening), SP (standard precision) and XP mode (extra precision) for analysis of docking simulations of hits (Table 8). We have also calculated binding energy calculations using Prime MM/GBSA module from Schrodinger suite (Table 8).

**Table 8:** Molecular docking and binding free energy analysis for studies ZINC hits.

Entry	PDB ID:4P8C			
	docking score (sp) kcal/mol	docking score (xp) kcal/mol	docking score (HTVS) kcal/mol	MMGBSA dG bind (sp complex) kcal/mol
ZINC19500480	− 7.076	− 6.58	− 5.95	− 61.073
ZINC19500487	− 7.465	− 6.157	− 6.369	− 63.63
ZINC07426404	− 7.274	− 7.288	− 6.337	− 58.203
ZINC02252037	− 6.173	− 6.03	− 6.438	− 45.513
ZINC03157457	− 5.74	− 5.08	− 5.357	− 44.357
ZINC03153031	− 5.61	− 6.274	− 5.86	− 46.604
ZINC19500488	− 7.595	− 6.63	− 5.558	− 55.149
ZINC12196803	**− 7.862**	**− 9.437**	**− 6.53**	**− 70.508**
ZINC19447057	− 6.531	− 5.842	− 5.901	− 52.235

Significant values are given in bold.

### Absorption, distribution, metabolism, excretion and toxicity predictions for dataset and newly identified ASINEX hits

We thoroughly carried out ADMET profiling studies using multiple commercial as well as free software tools including Schrödinger’s Qikprop, admetSAR, SwissADME and Pred-HERG (online Supplementary Tables S3–S10). We have calculated in-silico Drug-likeness and medicinal Chemistry for top 9 ASINEX molecules by SwissADME. Additionally, hERG blockage studies were also carried using popular “Pred-HERG” for new 9 hits. Boiled-egg model study was also incorporated for new hits to study the intestinal absorption profile using swissADME tool (online Supplementary Figs. S2, S7). Analysis of pharmacokinetics with CYP’s enzymes was done in silico with SwissADME (online Supplementary Table S5). QikProp calculations (Table 9) gave us ideas on pharmacokinetic parameters like rule of five, % human oral absorption, O/W coefficient, brain blood barrier permeability and caco cell permeability. For in-silico analysis of carcinogenicity and AMES test toxicity profiling were performed with popular online free utility called “admetSAR” V. 2.0 (http://lmmd.ecust.edu.cn/admetsar2/) (SI Table-S11)^32^.

**Table 9:** ADME predictions for computed for ZINC hits by QikProp^*^.

Entry	#stars	QPlogPo/w	QPPCaco	QPlogBB	#metab	QPlogKhsa	% Human oral absorption	Rule of five
ZINC19500480	0	1.795	366.379	− 0.92	3	− 0.302	83.342	0
ZINC19500487	0	2.311	342.159	− 1.249	3	− 0.087	85.834	0
ZINC07426404	0	2.955	422.838	− 1.154	4	0.07	91.253	0
ZINC02252037	0	2.902	1180.359	− 0.649	1	0.272	100	0
ZINC03157457	0	2.65	1181.999	− 0.546	1	0.158	100	0
ZINC03153031	0	3.447	1174.892	− 0.73	1	0.39	100	0
ZINC19500488	0	1.868	279.779	− 1.019	3	− 0.234	81.675	0
**ZINC12196803**	**0**	**2.203**	**1340.411**	**− 0.391**	**3**	**− 0.254**	**95.815**	**0**
ZINC19447057	1	3.08	1701.796	− 0.144	0	0.252	100	0

Significant values are given in bold.

^*^Recommended ranges are tabulated in supplementary information.

### Molecular dynamics simulation studies for best dock hits

We have carried out 1.2 ns and 5 ns molecular dynamics simulations (MDS) studies for our best dock hits (151:4P8C and ZINC12196803:4P8C complexes respectively) using Desmond 3.8 module (release 2017, Schrödinger, LLC, NY) incorporated into Schrodinger's interface in order to check the stability of the complexes (Figs. 9, 10) (online Supplementary Figs. S5, S9, S10). For MDS studies, we used the target protein 4P8C only by considering its importance with *mycobacteria*. We make use of OPLS-2005 MM force field for our initial steps during setup of MDS. For simulation of 151:4P8C complex, the counter ion/salt used was Na with concentration of 2.065 mM. Total of 59,725 atoms were simulated for 1.205 ns with NPT ensemble kept at temperature of 300.0 k (250 numbers of frames and 1.01325 bar pressure) using maestro version 11.1.011; 2017. For simulation of ZINC12196803:4P8C complex, we used same protocol as previous but total of 60,861 and 60,625 atoms were simulated for 1.205 (250 numbers of frames and 1.01325 bar pressure) and 5.008 ns respectively. The trajectory and number of frames kept at as obtained. The necessary MDS interactions were recorded from results obtained.

## Results and discussions

### Glide based molecular docking analysis

We had docked all the dataset as well as hits into binding cavity of protein 4P8C using Glide module V 8.9. We found that the dataset best docked molecule (Fig. 6) had docking score (XP mode) of − 9.068 kcal/mol; while that of standards isoniazid (INH = − 6.24 kcal/mol) and ethambutol (ETH = − 6.173 kcal/mol) had lesser docking scores (Table 4). We further used best docked dataset molecule 151 for ligand based virtual screening using “SwissSimilarity”. We also evaluated the docking score analysis for hits and found to be in range of − 5.61 to − 7.862 kcal/mol. The best docked hit ZINC12196803 (Fig. 8) had docking scores of − 9.437 kcal/mol (XP mode) and − 6.53 kcal/mol (HTVS mode) (Tables 8, 10). The molecule 151 had interactions with key amino acids in binding pockets of 4P8C. These were LYS418— –(N–C=O–NH) (hydrogen bond), GLY117—(–C=O–) (hydrogen bond) and non-polar interactions with THR118,PRO116,LEU115,PHE313 amino acid residues. The ASINEX best docked hit ZINC12196803 obtained with 2 side chains hydrogen bonding with TYR60, LYS418 and 1 backbone hydrogen bond with ARG58 amino acid (Fig. 8, Table 10). Other interactions includes hydrophobic interactions with ALA417, ARG58, ILE131, HIS132, etc. We had utilized same binding pocket as that of mentioned in materials and methods section. Figure 8 depicts 2D and 3D-interaction diagram for best dock molecules. We obtained RMSD values lesser than 2 Å indicating that docking was performed correctly.

**Table 10:** Comparative ligand–amino acid interactions carried with molecular docking for top 9 ASINEX HITS –for 4P8C(1–9), i.e. ZINC19500480,ZINC19500487,ZINC07426404,ZINC02252037,ZINC03157457,ZINC03153031, ZINC19500488,ZINC12196803, ZINC19447057 and standards (BTZ043, ETHAMBUTOL, INH) respectively.

Sr. no	Ligand/ASINEX hit	Amino acids involved during binding with target PDB ID: 4P8C
1	ZINC19500480	GLY 117 (H-bonding); LYS 418 (Pi-cation); TYR 60 (H-bonding); SER 58 (unspecified residue); SER59; TYR415; ALA417; VAL121; ILE131; CYS387; VAL365;PRO116;TYR314; LEU115; SER228; LYS367; HIS132; ASN 385; THR118; TRP16 (Hydrophobic)
2	ZINC19500487	GLY 117 (H-bonding); LYS 418 (Pi-cation); TYR 60 (H-bonding); SER 58 (unspecified residue); SER59; TYR415; ALA417; VAL121; ILE131; CYS387; VAL365; PRO116; TYR314; LEU115; SER228; LYS367; HIS132; ASN 385; THR118; TRP16 (Hydrophobic)
3	ZINC07426404	GLY 117 (H-bonding); HIS132(Hydrophobic; H-bonding); GLN334; CYS387; THR118; TYR60;ALA417;SER59; LYS418;VAL121;ILE131; PRO116;GLY133; PHE369;SER227; VAL365; ASN385; LEU317; LEU363
4	ZINC02252037	GLY 117 (H-bonding);ASN385(H-bonding); THR118; TYR60;ALA417;SER59; LYS418;VAL121;ILE131; PRO116;GLY133; PHE369;SER227; VAL365; ASN385; LEU317; LEU363
5	ZINC03157457	HIS132 (H-bonding); THR118; TYR60;ALA417;SER59; LYS418;VAL121;ILE131; PRO116;GLY133; PHE369;SER227; VAL365; ASN385; LEU317; LEU363
6	ZINC03153031	GLY 117 (H-bonding); TYR314(Hydrophobic); ASN385(H-bonding); VAL121;ILE131; PRO116;GLY133; PHE369;SER227; VAL365; ASN385; LEU317; LEU363
7	ZINC19500488	TYR60 (H-bonding); GLY 117 (H-bonding); PRO116;GLY133; PHE369;SER227; VAL121; ASN385; LEU317; LEU363; TYR415; ARG58; SER228;PRO316
8	ZINC12196803	LYS418(H-bonding); TYR60 (H-bonding);ARG58(H-bonding); VAL121;ILE131; PRO116;GLY133; PHE369;SER227; VAL365; ASN385; LEU317; LEU363
9	ZINC19447057	HIS132 (H-bonding); ILE131; PRO116; VAL121;ARG58;TYR415;VAL365
Std	BTZ043	TYR60 (H-bonding); GLY 117 (H-bonding); HIS132; VAL121;ILE131; PRO116; LEU363; TYR415; LEU317; ASN364; VAL365; LYS418
Std	ETHAMBUTOL	HIS315; GLY117; LEU317; SER246; TYR314 (Pi-Cation); ;ALA229; ALA244; VAL245; ASN364; PRO316; TRP230;VAL365; LYN134 (H-bonding); PHE313
Std	INH	GLY336; CYS387; GLY117; VAL365; LYN134; ASN385; GLY133; SER228; PHE369; LYS367;HIS132; TYR415; LYS418
Co-crystallized	Y22	GLY 117; LYS 418; TYR 60; SER 58; SER59; TYR415; ALA417; VAL121; ILE131; CYS387; VAL365;PRO116;TYR314; LEU115; SER228; LYS367; HIS132; ASN 385; THR118; TRP16

### MMGBSA (molecular mechanics generalized born surface area) binding energy calculations

We had carried out the evaluation of binding energies for all dataset as well as all hits with respect to standards such as isoniazid (INH), ethambutol (ETH). We found that best docked molecule 151:4P8C complex had MMGBSA dgBind energy of − 59.159 kcal/mol with respects to standards INH (− 30.214 kcal/mol) and ETH (− 39.290 kcal/mol). We also calculated binding free energy for ZINC12196803:4P8C complex and it was found to be − 70.508 kcal/mol. Our calculations suggested that hit molecule ZINC12196803 had better binding free energy and hence better stability rather than standards INH and ETH. Overall, results clarified the fact that our hits may have better stability (more negative binding energies) in terms of binding to protein 4P8C.

### Evaluation of MDS (molecular dynamics simulations) studies

During molecular docking simulations, the flexibility of protein may not involve in general. Studies pertaining to MDS were initially, obtained through Desmond 6.4 module (Schrödinger, LLC, NY, 2020) and finally with Groningen Machine for Chemical Simulations (GROMACS v5.1.5) with GROMACS 96-53a6 force fields^46–48^. We have analysed the molecular dynamics for our best dock hits 151:4P8C and ZINC12196803:4P8C complexes throughout the period of 1.2 ns, 5 ns (1200 ps to 5000 ps) and upto 100 ns (Figs. 9, 10) to confirm the exact modes of binding and to check stability of them. We have simulated both complexes with water molecules. These systems were set at particular temperature and pressure conditions. We observed that the atom-positional root-mean-square deviation (RMSD) plots for both complexes demonstrated stability of complexes throughout the simulation timing of 1200 ps to 5000 ps (1.2–5 ns) and 100 ns. Both MDS results retained exact interaction patterns as obtained from the molecular docking simulations. We noticed that there were fewer fluctuations among the RMSF (root mean square fluctuation) plots of 151:4P8C and ZINC12196803:4P8C complexes throughout the period of 1.2–100 ns (Fig. 10, SI. Fig. S11). Molecular dynamics simulations are representing closer connections to the physiological environmental conditions; and thus, will guide for better understanding of binding patterns. Figures 9, 10, depicts timeline representation plots as obtained from Desmond. Supplementary Fig. S11 analyses the stability of ZINC12196803:4P8C complex over a period of 100 ns.

### Evaluation of ADMET properties (absorption, distribution, metabolism, excretion and toxicity predictions)

We had calculated predictions of ADMET properties for our dataset as well as ASINEX hit molecules. For all docked dataset molecules, we obtained all QikProp (Schrödinger, LLC, NY, 2020) parameters within the standard range (Table 9). For our best docked hit molecule ZINC12196803, we obtained great QPPCaco cell permeability, 95.815% human oral absorption and 0 violations for Lipinski’s rule of five. The drug likeness and medicinal chemistry were also checked for both best docked hits i.e., 151 and ZINC12196803 using “SwissADME” online free software. Drug likeness studies showed us that both molecules had no violations for popular Lipinski’s rule, Ghose, Veber and Egan rules^49^. The bioactivity score for 151 was found to be 0.55, while synthetic accessibility as 2.93. SwissADME studies displayed that out dataset molecule 151 has solubility in water. As it falls in yellow region of boiled egg model, it may have probability to cross the blood–brain barrier (BBB). In order to explore other targets (for which these 151 molecules may act), we used “SwissSimilarity” tool. Our “SwissTargetPredition" analysis for 151 resulted into probable activities on 4 different classes (7% unclassified, 33% protease, 27% transporters and 33% on membrane receptors. Further, newly ligand based virtual screening hits were subjected for docking simulations. Among them, the best docked hit ZINC12196803 had same solubility (water soluble) and bioactivity score (0.55) as like 151. Our pharmacokinetic studies carried out using “SwissADME” suggested that all our newly searched hits had mixed profiling for BBB and p-gp substrate. All hits were found to be non-inhibitors for CYP1A2, CYP2D6 and CYP2C9 (except one). We have obtained mixed profiling for CYP2C19 inhibitions. Boiled egg model predictions were also depicted in mixed profiling of absorptions. We had also screened all hits for hERG blockade using online free tool called “pred-hERG” (online Supplementary Fig. S10). Our in-silico study for hERG blockade showed that ZINC12196803 molecule had no signs of cardio-toxicity as depicted in SI.

Finally, we have evaluated all new ASINEX hits for toxicity analysis using “admetSAR” (online Supplementary Table S11). With exception of 2 hits, all hits were found to be non-Ames toxic. All hits were also resulted in non-eye corrosion and non-eye irritation properties. We noticed that our predicted hit molecule ZINC12196803 may not have any kinds of toxicities as calculated from “admetSAR”. Acute oral toxicity for hit was also found to be 2.18 kg/mol.

### CPH (common pharmacophore) analysis

We have developed 14 CPH models, among them CPH AAAHR_1 (Fig. 3a) imparted good scores for modelling parameters like survival score of 5.93, phase hypo score of 1.36 and site score of 0.895. As top ranked CPH AAAHR_1 satisfied the standard criteria, we further employed this for 3D-QSAR studies. Figure shows how we aligned all ligands to top ranked CPH AAAHR_1 (Phase, 2020, Schrödinger, LLC, NY). Other CPH models with detailed scores are showed in Table. Our top ranked CPH AAAHR_1 shows 3Hydrogen bond acceptor (A), 1 hydrophobic group (H), and 1 aromatic ring (R) features as generated by Phase utility (Table 1).

### Evaluation of atom-based and field-based 3D-QSAR models with statistical parameters

**Figure 11:**
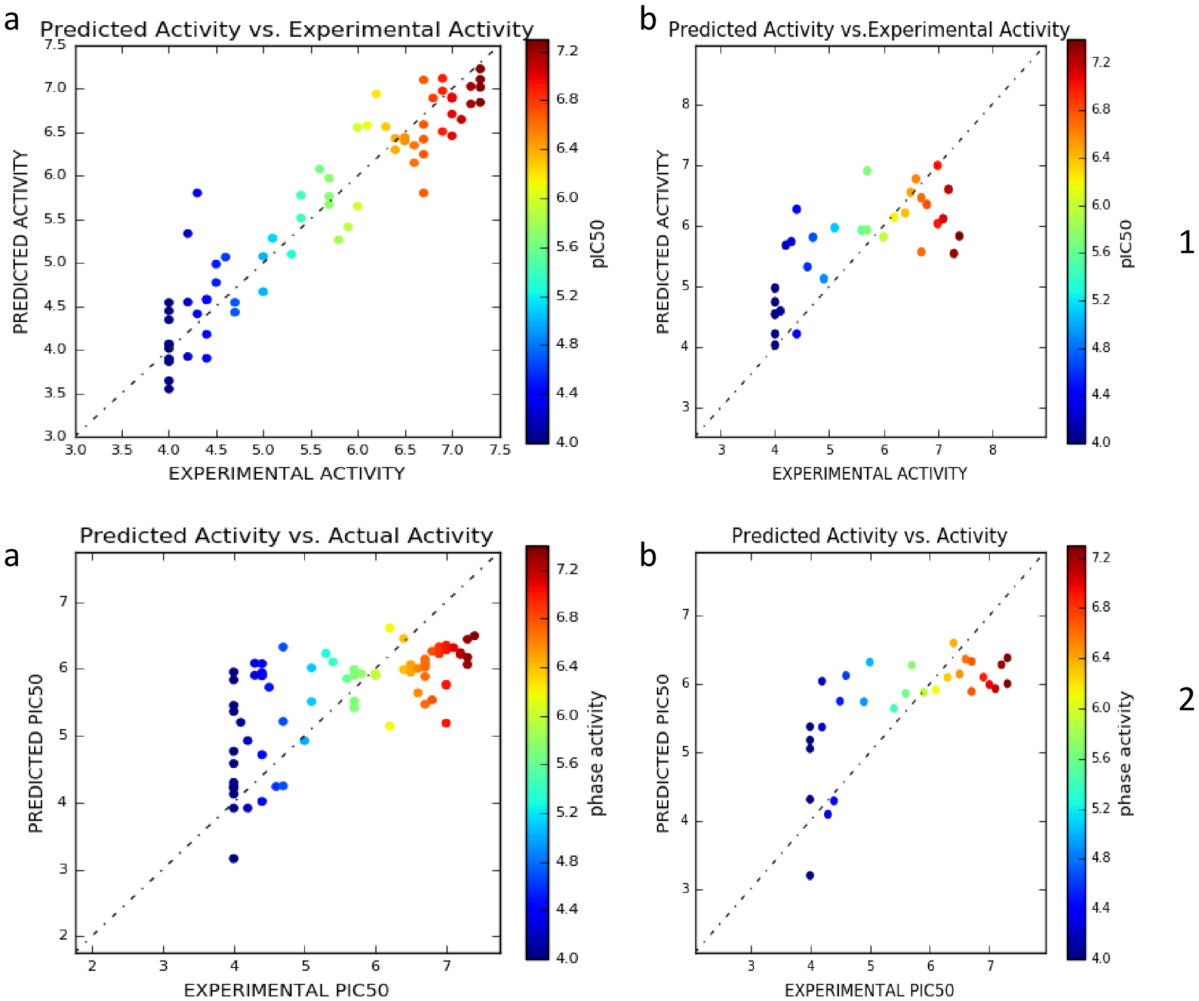
Graphical presentation of Actual versus (X-axis) Predicted pIC50 (Y-axis) of (**a**) training set molecules and (**b**) test set molecules for (1) Atom based, (2) Field based-3D QSAR models (this figure has been visualized from ‘Phase module’ Schrodinger, LLC, NY, V.2020, Available at: https://www.schrodinger.com/).

For effective reliability in generated atom- as well as field-based 3D-QSAR models, we utilized both internal as well as external validation parameters. Leave-one-out (LOO) cross-validation method was implemented in order to access the robustness, stability and predictive attributes of developed 3D-QSAR models. We analysed both models by using 32 and 30 test compounds for atom based- and field based-3D-QSAR models respectively (Tables 2 and 6). The atom based QSAR models were developed with PLS factor of 4, while in case of field based it was 3. The internal validation parameters for both atoms based- and field based-3D-QSAR models are depicted in tables. Both models were also evaluated with external validation parameters and for both they were obtained within standard criteria. For both the models, software generated scatters are shown in (Fig. 11). Finally, we noticed that our developed 3D-QSAR models had very high statistical significance.

### 3D-QSAR visualization

#### Visualization for atom-based 3D-QSAR

It can be seen that biological activities could be well correlated with structural patterns of the core moiety responsible for activity in the forms of occlusion maps. From the literature regarding atom based 3D-QSAR studies, blue occlusion maps/contours indicated incremental biological activity; while the red coloured occlusion maps/cubes corresponds to decrement (Schrodinger, LLC, NY, 2020). We have selected representative molecule 1 for aligning on top ranked 5 points CPH AAAHR_1 for effective visualization of QSAR occlusion maps. The occlusion maps surrounding 2,4-difluorobenzene motif showed blue occlusion maps indicating favoured region for electron withdrawing groups (Fig. 4a). While the entire remaining molecule represented mixed regions for electron withdrawing substitutions (EWS) (Fig. 4a). In case of occlusion maps corresponding to hydrogen bond donors (HBD); majority of red coloured maps (disfavoured regions) near to 2,4-difluorobenzene motif and imidazolidine-2,4-dione core were noticed (Fig. 4b). Figure 4c demonstrates majority of mixed regions for Hydrophobic substitutions (HS). We have observed red contours around methyl attached to imidazolidine-2,4-dione core indicating negative participations for negative ionic substitutions (Fig. 4d). The contours associated with other substitutions are depicted in Fig. 5. We have tabulated PLS parameters and atom type fractions in tables.

#### Analysis of field based-3D QSAR contour maps

For effective analysis of contour maps produced for 3D-QSAR, we selected molecule 1. We noticed that Gaussian Electrostatic field contour maps (Fig. 5a) surrounding (2,4-difluorobenzene –C=O–) attached to 2,4-dioxoimidazolidin core showed red contours indicating disfavoured electronegative regions. While, blue contour maps around 2,4-dioxoimidazolidin suggested favoured electropositive substitutions. Gaussian Hydrogen bond acceptor field contours (Fig. 5b) around 2,4-difluorobenzene showed disfavoured (magenta) regions. Red occlusion maps around (Ar–C=O-2,4-dioxoimidazolidin core depicted favoured Gaussian Hydrogen bond acceptor fields. Gaussian Hydrogen bond donor occlusion maps (Fig. 5c) near to 2,4-difluorobenzene demonstrated disfavoured (cyan) regions, while occlusion maps near to nitrile attached arene motif showed favoured purple coloured maps. Gaussian Steric field occlusion maps (Fig. 5d) at 2,4-difluorobenzene depicting both favored (green) unfavorable (yellow) regions for substitutions. White occlusion maps surrounding the entire (2,4-difluorobenzene –C=O–) attached to 2,4-dioxoimidazolidin core indicates disfavoured regions for Gaussian Hydrophobic fields (Fig. 5e). All software generated occlusion maps are shown in the figure. Tables, depicts field fractions and statistical parameters for generated field-based 3D-QSAR models.

#### Relevance of obtained validation parameters with reliability of 3D-QSAR models

The Modeling statistical parameters of the selected model have passed the acceptability criteria proposed by Golbraikh and Tropsha as depicted with Q^2^ > 0.5, R^2^_train_ > 0.6, R^2^_test_ > 0.6, ∣r^2^_0_ − r^2^_0_′∣∣ < 0.3, 0.85 < k < 1.15, 0.85 < k′ < 1.15, (r^2^ − r_0_^2^)/r^2^ < 0.1 and (r^2^ − r′_0_^2^)/r^2^^50^. r^2^ is the fraction of the total variation in the dependent variables that is explained by the regression equation. Higher r^2^ closer to 0.9 but 0.6, is considered as good indication of QSAR model. In case of atom-based 3D-QSAR model we obtained lower RMSE 0.86. It has been widely accepted that a low RMSE value indicates that the simulated and observed data are close to each other showing a better accuracy. Thus lower the RMSE better is model performance. Further, SD is the standard deviation about the regression line. This is a measure of how well the function derived by the QSAR analysis predicts the observed biological activity. The smaller the value of SD the better is the QSAR. In cases of our developed 3D-QSAR models, the best models retained with lower values of SD (SD for atom-based 3D-QSAR: 0.419; SD for field-based 3D-QSAR: 0.6865). The statistical significance of the regression model can be assessed by means of the Fisher statistic (F). The F-value or variance ratio is the ratio between explained and unexplained variance for a given number of degrees of freedom, respectively *p* and (n = p = 1), where *n* are the chemicals and *p* the model descriptors. For our set of developed models in atom-based 3D-QSAR, the one we selected as best model retained with F value of 119.7. Similarly, one can analyse the relevance of such statistical significances using the description attached in the *supporting information*.

### Evaluation of QSARINS based MLR models

The top most model with higher statistical significance, as demonstrated by internal and external validations calculations, was further analysed for its interpretations. The developed model-1 is represented by the below MLR equation:

#### Multivariate models

**Model 1** (70% training: 30% test set, 6 parametric)

1 $$\begin{aligned} pIC_{50} &= - 10.5590\left( { \pm 7.1202} \right) - 0.0005\left( { \pm 0.3267} \right)*ATSC7m \hfill \\ & \quad - 0.1252\left( { \pm 0.2216} \right)*AATSC4v - 4.4124\left( { \pm 0.2212} \right)*GATS4i \hfill \\ & \quad + 5.7006\left( { \pm 0.4025} \right)*SpMax5\_Bhe - 0.1334\left( { \pm 0.4632} \right)*SHBint6 \hfill \\ & \quad + 0.2850\left( { \pm 0.5919} \right)*minHBint5 \hfill \\ \end{aligned}$$

#### QSAR model interpretation

In currently developed 6 parametric **model-1 (1)** from QSARINS, the descriptor **ATSC7m** represents Centred Broto-Moreau autocorrelation of lag 7 weighted by mass. This descriptor has negative correlations with the biological activity (BA) as denoted by examples such as [**141** (pIC_50_ = 4.1), **142** (pIC_50_ = 6.0)] and [**183** (pIC_50_ = 7.2), **185** (pIC_50_ = 6.2)]. The descriptor, **AATSC4v** denotes Average centered Broto-Moreau autocorrelation—lag 4/weighted by van der Waals volumes and found to have negative correlations with BA, which can be seen with examples such as [**112** (pIC_50_ = 6.9), **118** (pIC_50_ = 6.6)], [**122** (pIC_50_ = 4), **123** (pIC_50_ = 4.5)], etc. Descriptors, Geary autocorrelation of lag 4 weighted by ionization potential (**GATS4i**) and Sum of E-State descriptors of strength for potential hydrogen bonds of path length 6 (**SHBint6**) were represented decreasing trends with the decrease in the values of descriptors. Further, we noticed that descriptors **SpMax5_Bhe** (Largest absolute eigenvalue of Burden modified matrix—n 5/weighted by relative Sanderson electronegativities) and **minHBint5** were positively correlated with the BA. The model also contained 2D atom type electro-topological state descriptor minHBint5 which is defined as Minimum E-State descriptors of strength for potential Hydrogen Bonds of path length 5 (minHBint5). These descriptors indicated the importance of hydrogen bonds of path length 5. Examples of compounds following this trend includes, [**114** (pIC_50_ = 7.4), **115** (pIC_50_ = 6.5)], [**116** (pIC_50_ = 7.3), **122** (pIC_50_ = 4)], etc.

Full details of statistical analysis, graphs of experimental vs predicted pIC_50_ values for model 1 (b) William’s plot for model 1; (c) Insubria plot for model 1 (d) Y-scrambling plot for model 1 have been enclosed in the supporting information (please refer online Supplementary Fig. S13). Furthermore, values for the various cross-validation properties supported statistical robustness of GA-MLR QSAR model with (R^2^_cv_, RMSE_cv_, MAE_cv_, CCC_cv_, and Q^2^_LMO_). Higher values for R^2^_ex_, Q^2^F^1^, Q^2^F^2^, Q^2^F^3^, Golbraikh and Tropsha criteria and CCC_ex_ depicted the external predictive power of the developed models **1** (please refer online Supplementary, Tables S12–14)^51^.

Therefore, creating QSAR models with various molecular descriptors and broad chemical spaces will undoubtedly offer insightful information on the causes of variations in the anti-DPRE1 activity of hydantoin-based inhibitors. We acknowledge the limits of the QSAR models that have been established so far, but better models would result from having more descriptor calculation data, accurate modelling, and fewer statistical interferences^36^. Consequently, each model generated here demonstrates the integration of all chosen chemical descriptors and thus forecasts potential pIC_50_ values for the aforementioned analogues.

## Conclusion

To conclusion, we have developed statistically robust QSARINS based GA-MLR, atom-based as well as field based-3D-QSAR models with robust training set, R^2^ > 0.69 and test set, Q^2^ > 0.50 parameters. Our generated top ranked 5 point hypothesis AAAHR_1, which had 3 hydrogen bond acceptors (A), 1 hydrophobic group (H), and 1 aromatic ring (R) features depicted best survival score among 14 developed hypotheses as generated by Phase utility. Our dataset best docked molecule, 151 had goo docking scores (XP mode) of -9.068 kcal/mol; while that of standards isoniazid (INH = − 6.24 kcal/mol) and ethambutol (ETH = − 6.173 kcal/mol) had lesser docking scores than it. The molecule 151 had interactions with key amino acids in binding pockets of enzyme DPRE1 (4P8C). These were found to be LYS418— –(N–C=O–NH) (hydrogen bond), GLY117—(–C=O–)(hydrogen bond) and non-polar interactions with THR118,PRO116,LEU115,PHE313 amino acid residues. Finally, our best docked 151was queried for identifications of new hits using “SiwssSimilarity”. Among the top 9 ASINEX hits, the hit molecule ZINC12196803 had best binding energies and docking score (docking score = − 9.437 kcal/mol, MMGBSA dgBind = − 70.508 kcal/mol). We observed that RMSD plots for 151:4P8C and ZINC12196803:4P8C complexes demonstrated good stabilities throughout the simulation timings of 1200 ps to 10,000 ps (1.2–100 ns). Our pharmacokinetic studies carried out using SwissADME, QikProp, pred-hERG and admetSAR demonstrated that all our newly searched hits had mixed profiling for BBB and p-gp substrate. All hits were found to be non-inhibitors for CYP1A2, CYP2D6 and CYP2C9 (except one). Finally, we can say that our different combinations of computational techniques for identifications of new DPRE1 inhibitors may pave new way towards future developments.

## Supplementary Information

Supplementary Information.

## Data Availability

The dataset used in the manuscript is publicly available from below repositories. (1) Repository Name: Protein Database Bank; Deposited Date by source authors: 2014-03-31; Released Date: 2014-12-10; Accession Number: 10.2210/pdb4P8C/pdb; Deposition by original Author(s): Neres, J., Pojer, F., Cole, S.T. Macromolecular structure: 4p8c [link to the repository: https://www.rcsb.org/structure/4p8c] and originally deposited from article, https://pubmed.ncbi.nlm.nih.gov/25427196/. (2) Other Data can be made available from authors with reasonable request.

## Abbreviations

3D-QSAR model: 3D-quantitative structure–activity relationship models
Prime/MM-GBSA: Prime/molecular mechanics generalized born surface area
CPH: Common pharmacophore hypothesis
R^2^: Correlation coefficient
Q^2^: Cross-validation correlation coefficient
RMSE: Root mean square error
PDB: Protein Databank
ADMET: Absorption, distribution, metabolism, excretion and toxicity properties
MIC: Minimum inhibitory concentration
IC_50_: Inhibitory concentration
PLS: Partial least squares
SP: Standard precision
#stars: Number of property or descriptor values that fall outside the 95% range of similar values for known drugs
QPlogPo/w: Predicted octanol/water partition coefficient
QPPCaco: Predicted apparent Caco-2 cell permeability in nm/sec.
Percent Human Oral Absorption: Predicted human oral absorption on a 0 to 100% scale.
Rule of Five: Number of violations of Lipinski’s rule of five
