# Supplementary material for: Identification of hydantoin based Decaprenylphosphoryl-β-d-Ribose Oxidase (DprE1) inhibitors as antimycobacterial agents using computational tools
Source: Sci Rep. 2022 Sep 30;12:16368. doi: 10.1038/s41598-022-20325-1 (PMC9525719; doi:10.1038/s41598-022-20325-1)
Supplement: Supplementary file 1 — Supplementary Information. [file 41598_2022_20325_MOESM1_ESM.docx]

**Supplementary information**

***Relevance of statistical parameters in QSAR Modeling***

***Internal validation***

The QSAR model generated was internally validated using cross-validation technique. In essence, this technique provides adequate information about the predictive reliability of the QSAR equation. The leave-one-out cross-validation technique was adopted in this research and the cross-validated Q^2^cv was evaluated based on expression.

**
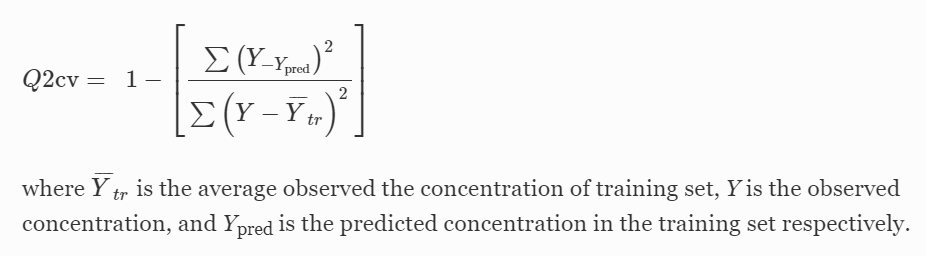
**

***External validation***

The following statistical features of the test set were proposed by Golbraikh and Tropsha for a robust QSAR model with good predictive potential.


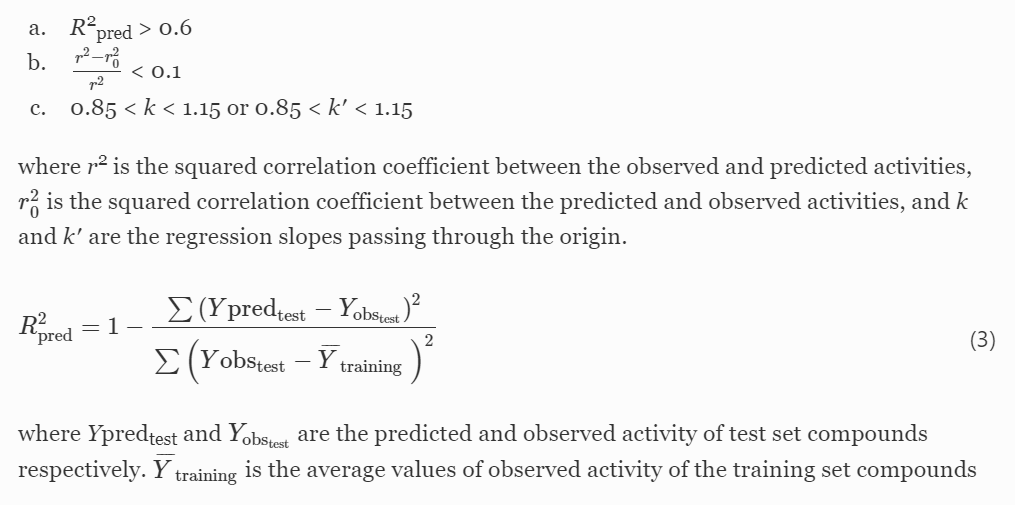


**Statistical parameters for used for validation of QSAR models**


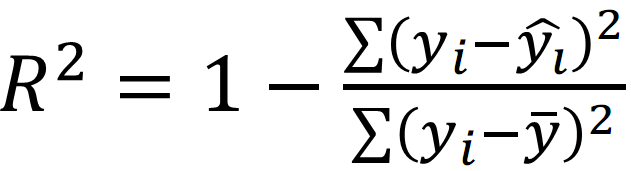


where y_i_ are the observed values of the response, ȳ the corresponding average, ŷ are the calculated values


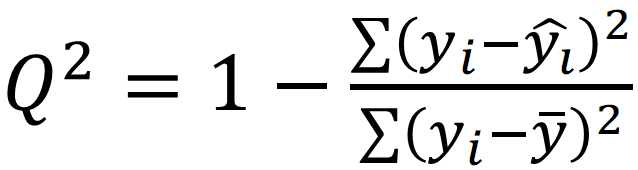


where y_i_ are the observed values of the response, ȳ the corresponding average, ŷ are the values predicted for each object when it is not in the training set.


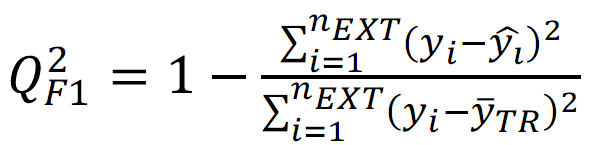


where y_i_ are the observed values of the response, ȳ the corresponding average, ŷ are the calculated values


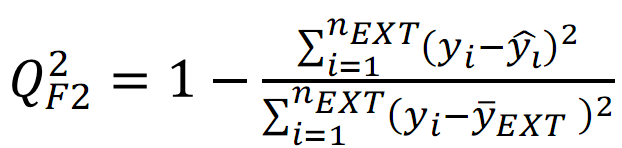


where y_i_ are the observed values of the response, ȳ the corresponding average, ŷ are the calculated values


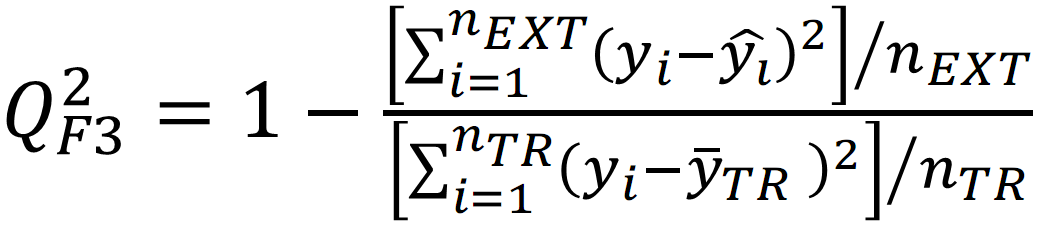


where y_i_ are the observed values of the response, ȳ the corresponding average, ŷ are the calculated values


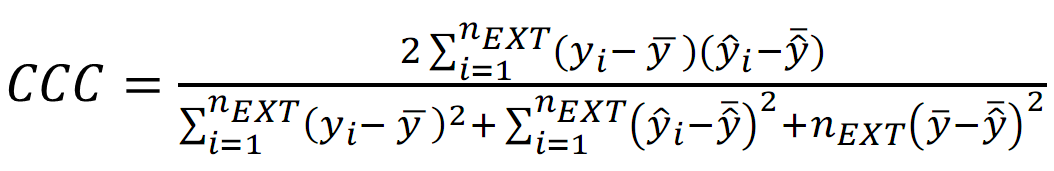


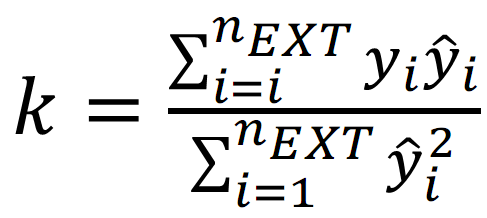


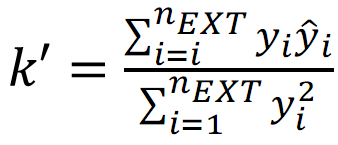


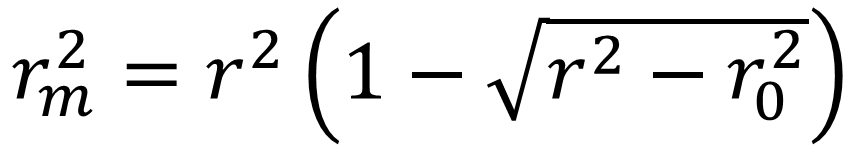


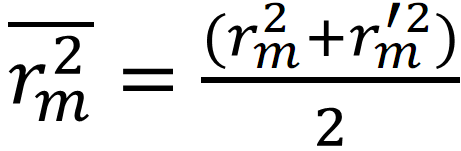


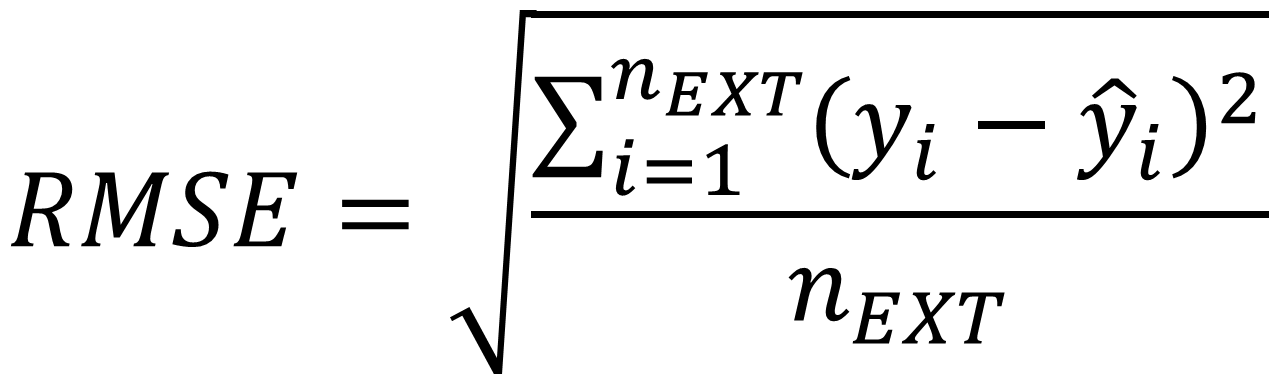


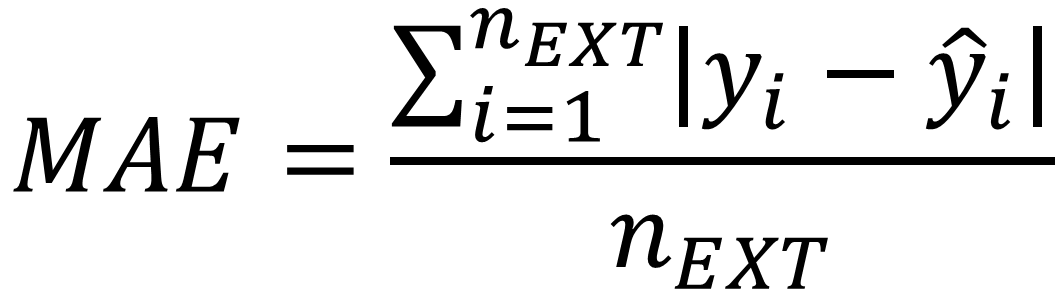


where y_i_ are the observed values of the response, ȳ the corresponding average, ŷ are the calculated values.

Table S1. Experimental dataset employed for 3D-QSAR study along with actual pIC_50_ (µM) values.

- Original dataset is available in the article: [M.K. Rogacki, E. Pitta, O. Balabon, S. Huss, E.M. Lopez-Roman, A. Argyrou, D. Blanco-Ruano, M. Cacho, C.M. Vande Velde, K. Augustyns, and L. Ballell, Identification and Profiling of Hydantoins—A Novel Class of Potent Antimycobacterial DprE1 Inhibitors, J. Med. Chem., 61(2018), pp.11221-11249].

| Ligand Name | Ligand Structures | Actual Activity |
| --- | --- | --- |
| 1 |  | 7 |
| 4 |  | 4.3 |
| 7 |  | 6.7 |
| 24 |  | 6.7 |
| 25 |  | 6.7 |
| 26 |  | 6 |
| 27 |  | 6 |
| 28 |  | 4.2 |
| 29 |  | 4 |
| 30 |  | 4 |
| 31 |  | 4 |
| 32 |  | 4 |
| 33 |  | 4 |
| 34 |  | 4 |
| 35 |  | 4.3 |
| 36 |  | 4.4 |
| 37 |  | 4 |
| 38 |  | 5.4 |
| 39 |  | 4.6 |
| 40 |  | 4.4 |
| 41 |  | 5 |
| 42 |  | 4 |
| 43A |  | 4 |
| 43B |  | 4.2 |
| 44 |  | 4.2 |
| 46 |  | 4 |
| 47 |  | 4 |
| 109 |  | 7.1 |
| 110 |  | 6.1 |
| 111 |  | 7 |
| 112 |  | 6.9 |
| 113 |  | 7.3 |
| 114 |  | 7.4 |
| 115 |  | 6.5 |
| 116 |  | 7.3 |
| 117 |  | 7.1 |
| 118 |  | 6.6 |
| 119 |  | 5.6 |
| 120 |  | 6.8 |
| 121 |  | 4.4 |
| 122 |  | 4 |
| 123 |  | 4.5 |
| 124 |  | 5.7 |
| 125 |  | 6.5 |
| 126 |  | 6.7 |
| 127 |  | 6.9 |
| 128 |  | 4 |
| 129 |  | 4.4 |
| 130 |  | 6.4 |
| 131 |  | 7.3 |
| 132 |  | 4 |
| 133 |  | 6.9 |
| 134 |  | 7.3 |
| 135 |  | 6.2 |
| 136 |  | 4.7 |
| 137 |  | 5.1 |
| 138 |  | 6.7 |
| 139 |  | 7 |
| 140 |  | 4.2 |
| 141 |  | 4.1 |
| 142 |  | 6 |
| 143 |  | 6.6 |
| 149 |  | 5.8 |
| **151** | **** | **4** |
| 155 |  | 5.1 |
| 156 |  | 4.9 |
| 157 |  | 4.5 |
| 160 |  | 4.7 |
| 161 |  | 4 |
| 163 |  | 4 |
| 180 |  | 7.2 |
| 181 |  | 6.4 |
| 182 |  | 7.3 |
| 183 |  | 7.2 |
| 184 |  | 5.7 |
| 185 |  | 6.2 |
| 186 |  | 5.9 |
| 187 |  | 4.4 |
| 188 |  | 6.5 |
| 189 |  | 5.6 |
| 190 |  | 6.3 |
| 191 |  | 5.7 |
| 192 |  | 6.7 |
| 193 |  | 7 |
| 194 |  | 6.8 |
| 195 |  | 5.7 |
| 196 |  | 6.7 |
| 197 |  | 5.7 |
| 198 |  | 4.7 |
| 199 |  | 5.3 |
| 200 |  | 4.6 |
| 201 |  | 6.6 |
| 202 |  | 6.4 |
| 203 |  | 5.4 |
| 204 |  | 7 |
| 205 |  | 5 |
| 206 |  | 7 |
| 207 |  | 7.2 |
| 208 |  | 4.3 |
| 209 |  | 4.4 |

Figure S1. Summary of Filed based 3D-QSAR for representative molecule 1.

**Table S2:** External validation parameters for 3D-QSAR developed for Hypothesis ***AAAHR_1.***

| External Validation | Parameter Found | Limitation |
| --- | --- | --- |
| r^2^_CV_ | **>0.5** | **r^2^_CV_ >0.5** |
| R | **Close to 1** | **Close to 1** |
| r^2^ | **>0.6** | **r^2^ >0.6** |
| k value | **0.85<k<1.15** | **0.85<k<1.15** |
| k’ value | **0.85<k’<1.15** | **0.85<k’<1.15** |
| R^2^_0_ | **Close to r^2^** | **Close to r^2^** |
| R’^2^_0_ | **Close to r^2^** | **Close to r^2^** |
| r^2^_m_ | **>0.5** | **r^2^_m_ >0.5** |
| r^2^_pred_ | **>0.5** | **r^2^_pred_>0.5** |

r^2^_CV_ is a cross validated coefficient. R (or r^2^) is a correlation coefficient between actual and predicted values. k and k’ are the slope values of regression lines. R^2^_0_ and R’^2^_0_ are correlation coefficient between regression lines through origin. r^2^_m_ is a modified squared correlation coefficient using LOO method. r^2^_pred =_ Predictive correlation coefficient value.

151

Table S3- In-silico calculation of Drug-likeness and medicinal Chemistry for the most docked molecule 151 by SwissADME.

| COMP.ID | Lipinski | Ghose | Veber | Egan | Muegge | Bioavailability Score |
| --- | --- | --- | --- | --- | --- | --- |
| 151 | Yes; 0 violation | Yes; 0 violation | Yes; 0 violation | Yes; 0 violation | Yes; 0 violation | 0.55 |
| Medicinal Chemistry | | | | | | |
| COMP.ID | PAINS | Brenk | Leadlikeness | Synthetic accessibility | | |
| 151 | 0 alert | 0 alert | No; 1 violation: MW>350 | 2.93 | | |

Table S4-In-silico calculation of water solubility for 151 by SwissADME.

| COMP.ID | Log S (ESOL) | Solubility | Class | Log S (Ali) | Solubility | Class | Log S (SILICOS-IT) | Solubility | Class |
| --- | --- | --- | --- | --- | --- | --- | --- | --- | --- |
| 151 | -3.74 | 6.52e-02 mg/ml ; 1.81e-04 mol/l | Soluble | -3.51 | 1.11e-01 mg/ml ; 3.08e-04 mol/l | Soluble | -6.39 | 1.47e-04 mg/ml ; 4.07e-07 mol/l | Poorly soluble |


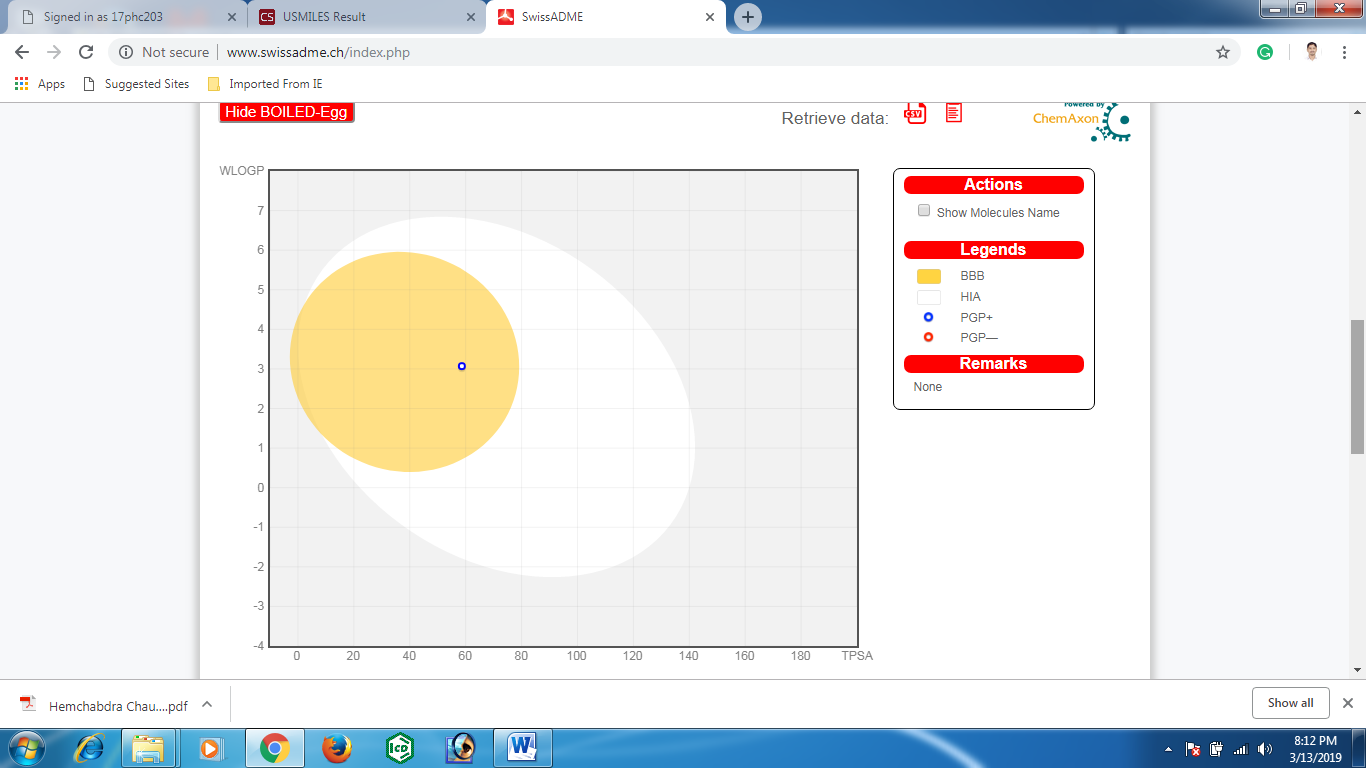


Figure S2-Predicted Boiled egg model for intestinal passive absorption of 151 by SwissADME:


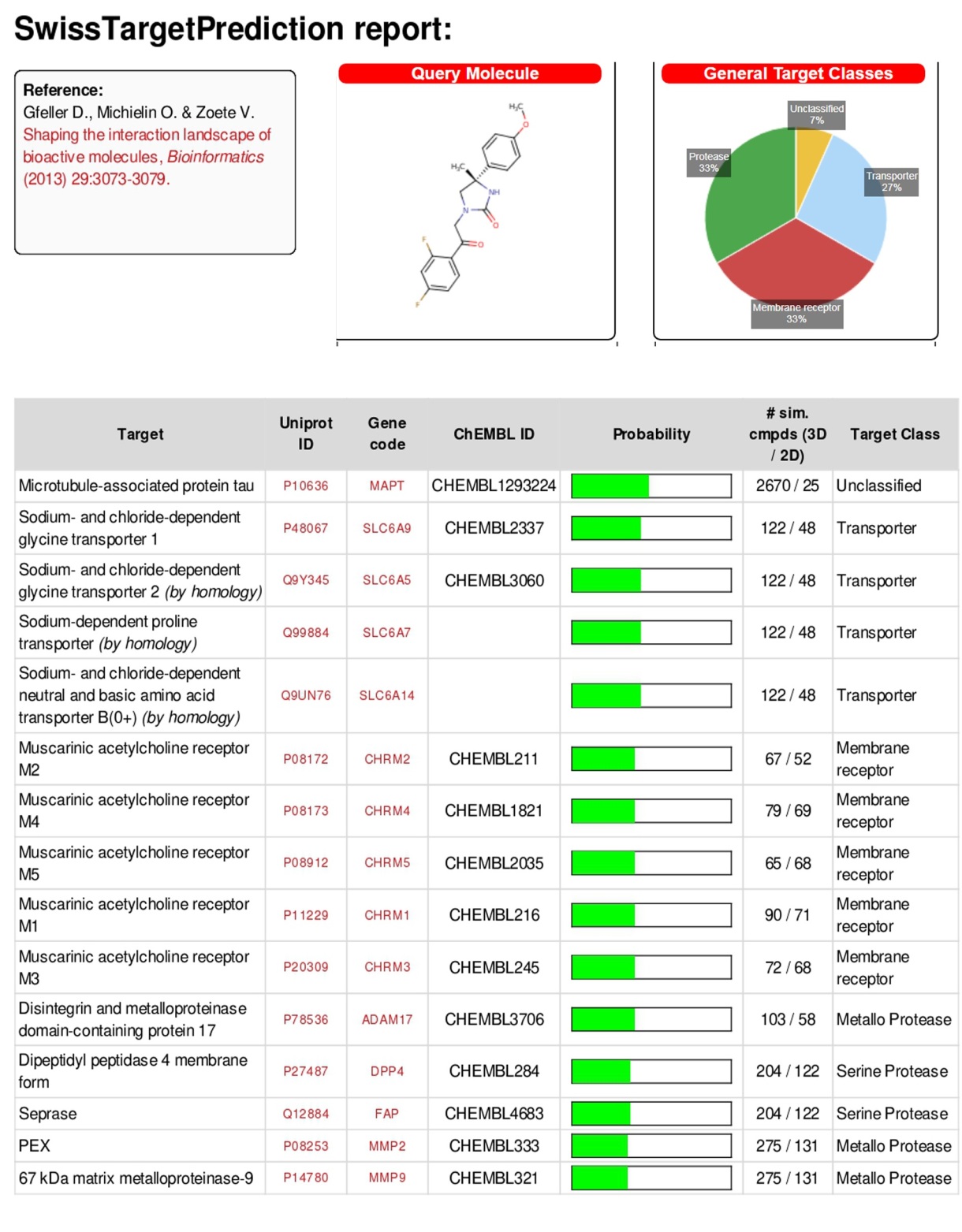


Figure S3- Target prediction for most docked molecule 151 by using SwissTargetprediction tool:


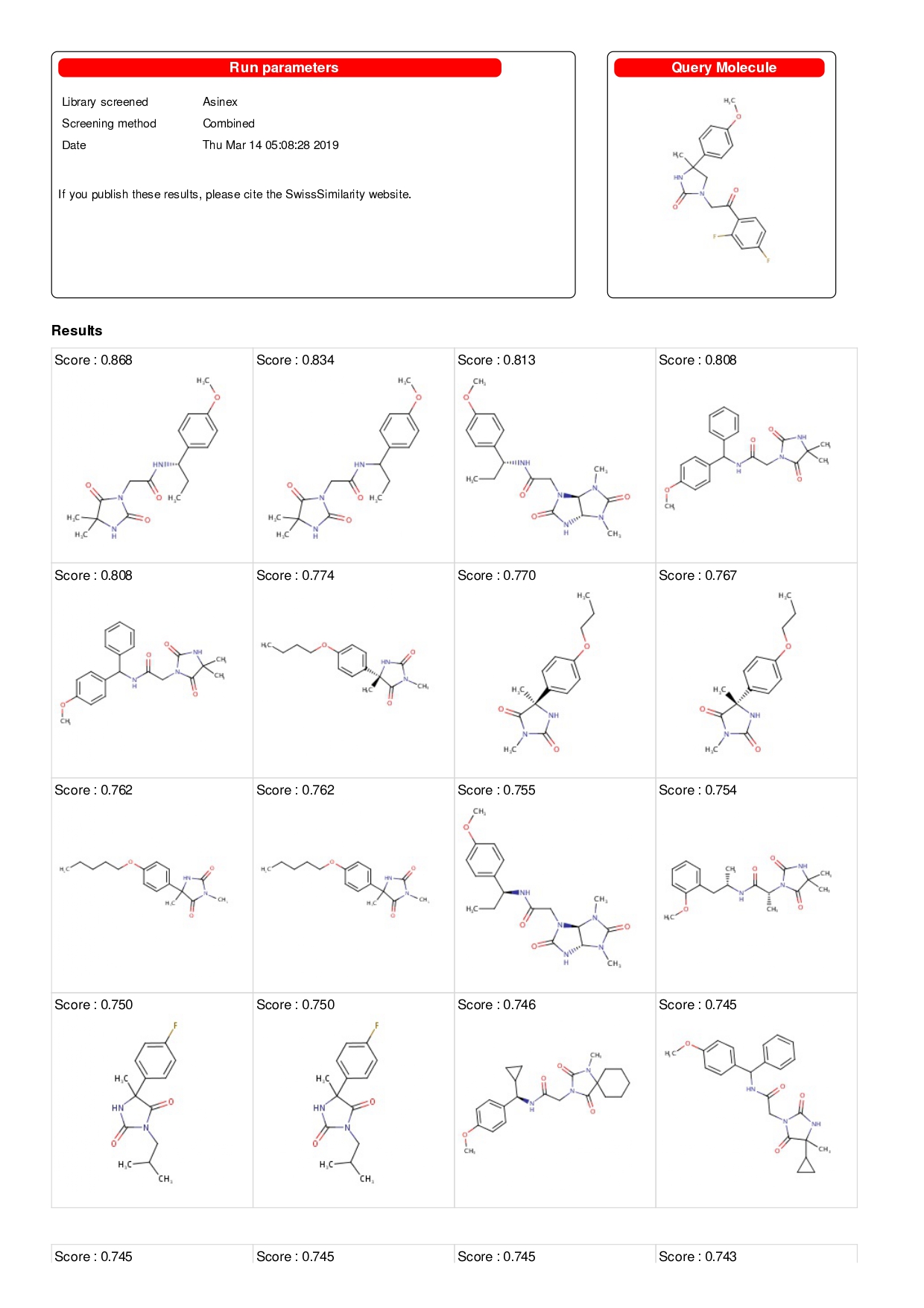

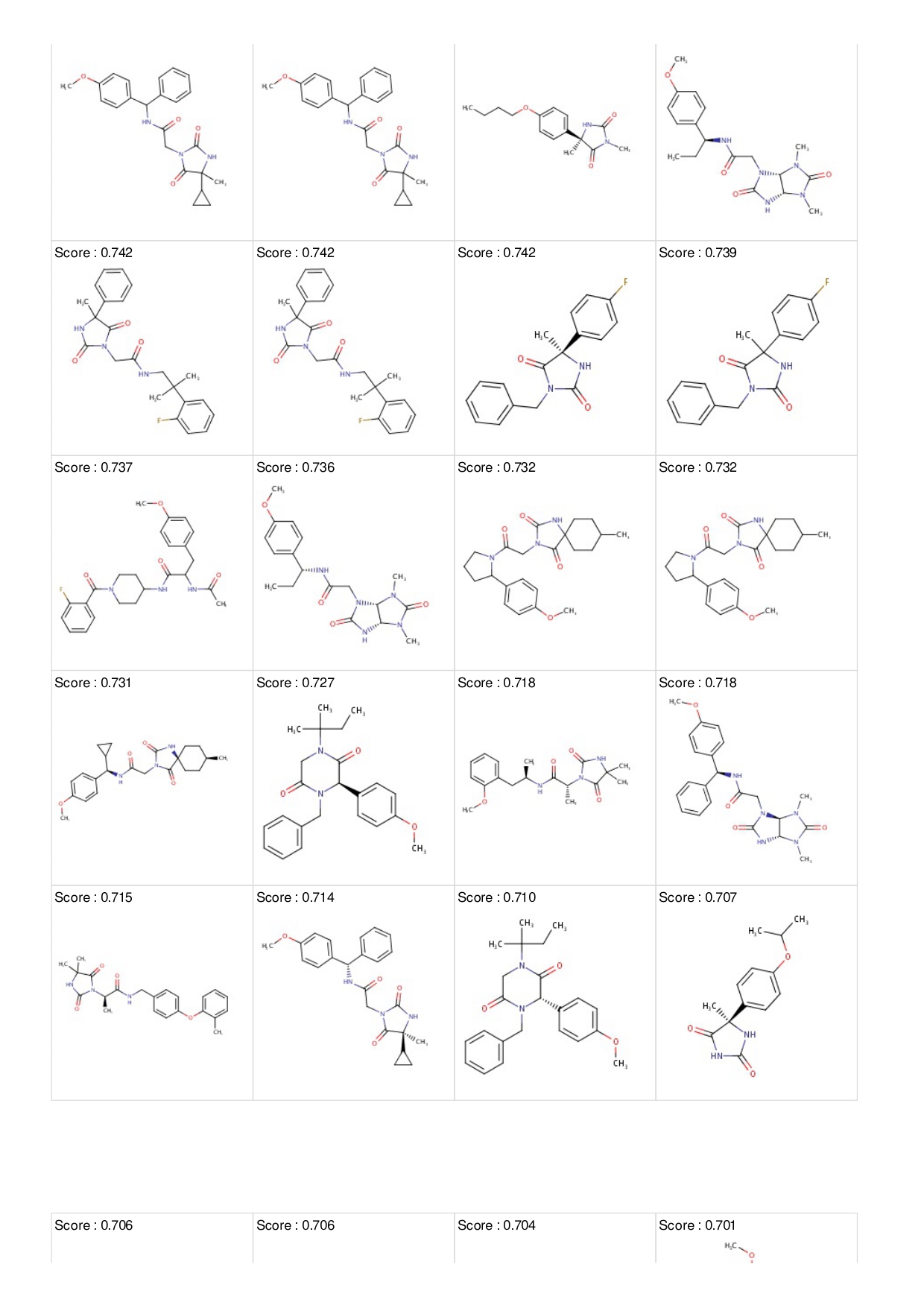


Figure S4 – Ligand based screening for most docked molecule151 by using SwissSimilarity tool:

Table S5- *In-silico* Pharmacokinetics profile of the compounds as obtained from SwissADME server.

| Entry | GI absorption | BBB permeant | P-gp substrate | CYP1A2 inhibitor | CYP2C19 inhibitor | CYP2C9 inhibitor | CYP2D6 inhibitor | CYP3A4 inhibitor | Log K_p_ (skin permeation) |
| --- | --- | --- | --- | --- | --- | --- | --- | --- | --- |
| 1 | High | NO | NO | NO | YES | YES | NO | YES | -6.88 cm/s |
| 4 | High | YES | NO | NO | YES | YES | NO | YES | -6.73 cm/s |
| 7 | High | NO | NO | NO | YES | YES | NO | YES | -6.88 cm/s |
| 24 | High | YES | NO | NO | YES | YES | NO | YES | -6.73 cm/s |
| 25 | High | YES | NO | NO | YES | YES | NO | YES | -6.30 cm/s |
| 26 | High | NO | NO | NO | YES | YES | NO | NO | -6.85 cm/s |
| 27 | High | YES | NO | NO | YES | YES | NO | YES | -6.70 cm/s |
| 28 | High | YES | NO | NO | YES | YES | NO | YES | -6.26 cm/s |
| 29 | High | YES | NO | NO | YES | YES | NO | YES | -6.37 cm/s |
| 30 | High | YES | NO | NO | YES | YES | YES | YES | -6.22 cm/s |
| 31 | High | YES | NO | NO | YES | YES | NO | YES | -5.78 cm/s |
| 32 | High | YES | NO | NO | YES | YES | NO | NO | -6.61 cm/s |
| 33 | High | YES | NO | NO | YES | YES | YES | YES | -6.46 cm/s |
| 34 | High | YES | NO | NO | YES | YES | NO | YES | -6.03 cm/s |
| 35 | High | NO | NO | NO | YES | YES | NO | YES | -7.00 cm/s |
| 36 | High | YES | NO | NO | YES | YES | NO | YES | -6.85 cm/s |
| 37 | High | YES | NO | NO | YES | YES | NO | YES | -6.41 cm/s |
| 38 | High | NO | NO | NO | YES | YES | NO | YES | -6.69 cm/s |
| 39 | High | NO | NO | NO | YES | YES | NO | YES | -6.84 cm/s |
| 40 | High | NO | NO | NO | YES | YES | NO | YES | -6.71 cm/s |
| 41 | High | YES | NO | NO | YES | YES | NO | YES | -6.50 cm/s |
| 42 | High | NO | YES | NO | YES | YES | YES | YES | -7.07 cm/s |
| 43A | High | NO | NO | NO | NO | YES | NO | NO | -6.79 cm/s |
| 43B | High | NO | NO | NO | NO | YES | NO | NO | -6.79 cm/s |
| 44 | High | NO | YES | NO | NO | NO | YES | YES | -7.15 cm/s |
| 46 | High | YES | NO | NO | YES | NO | NO | NO | -6.63 cm/s |
| 47 | High | YES | NO | NO | YES | YES | NO | NO | -6.19 cm/s |
| 109 | High | YES | NO | NO | YES | YES | NO | YES | -6.52 cm/s |
| 110 | High | YES | NO | NO | YES | YES | NO | YES | -6.36 cm/s |
| 111 | High | YES | NO | NO | YES | YES | NO | YES | -6.56 cm/s |
| 112 | High | YES | NO | NO | YES | YES | NO | YES | -6.32 cm/s |
| 113 | High | NO | NO | NO | YES | YES | NO | YES | -6.20 cm/s |
| 114 | High | YES | NO | NO | YES | YES | NO | YES | -6.28 cm/s |
| 115 | High | NO | NO | NO | YES | YES | NO | YES | -7.11 cm/s |
| 116 | High | NO | NO | NO | YES | NO | NO | NO | -7.44 cm/s |
| 117 | High | NO | YES | NO | YES | YES | YES | YES | -7.23 cm/s |
| 118 | High | NO | YES | NO | YES | YES | NO | YES | -7.38 cm/s |
| 119 | High | NO | YES | NO | NO | NO | NO | YES | -7.46 cm/s |
| 120 | High | NO | NO | NO | YES | NO | NO | NO | -7.55 cm/s |
| 121 | High | NO | NO | NO | YES | YES | NO | YES | -6.88 cm/s |
| 122 | High | YES | NO | NO | YES | YES | NO | YES | -6.73 cm/s |
| 123 | High | YES | NO | NO | YES | YES | NO | YES | -6.52 cm/s |
| 124 | High | YES | NO | NO | YES | YES | NO | YES | -6.77 cm/s |
| 125 | High | YES | NO | NO | YES | YES | NO | YES | -6.77 cm/s |
| 126 | High | YES | NO | NO | YES | YES | NO | YES | -6.56 cm/s |
| 127 | High | NO | NO | NO | YES | YES | NO | YES | -6.48 cm/s |
| 128 | High | NO | NO | NO | YES | YES | NO | YES | -7.14 cm/s |
| 129 | High | NO | NO | NO | YES | NO | NO | NO | -7.30 cm/s |
| 130 | High | NO | NO | NO | YES | NO | NO | YES | -7.05 cm/s |
| 131 | High | NO | NO | NO | YES | NO | NO | YES | -7.26 cm/s |
| 132 | High | NO | NO | NO | YES | YES | NO | YES | -6.89 cm/s |
| 133 | High | YES | NO | NO | YES | YES | NO | YES | -6.23 cm/s |
| 134 | High | NO | NO | NO | YES | YES | NO | YES | -6.59 cm/s |
| 135 | High | YES | NO | NO | YES | YES | NO | YES | -6.06 cm/s |
| 136 | High | YES | NO | NO | YES | YES | NO | YES | -6.01 cm/s |
| 137 | High | NO | NO | NO | YES | YES | NO | YES | -6.32 cm/s |
| 138 | High | YES | NO | YES | YES | YES | NO | YES | -6.58 cm/s |
| 139 | High | NO | YES | NO | NO | NO | NO | NO | -7.83 cm/s |
| 140 | High | NO | YES | NO | NO | NO | NO | NO | -7.83 cm/s |
| 141 | High | NO | YES | NO | NO | NO | NO | NO | -7.11 cm/s |
| 142 | High | NO | NO | NO | YES | NO | NO | NO | -7.41 cm/s |
| 143 | High | NO | NO | NO | YES | NO | NO | NO | -7.62 cm/s |
| 149 | High | YES | NO | NO | YES | YES | NO | YES | -6.39 cm/s |
| 151 | High | YES | YES | NO | YES | YES | YES | YES | -6.63 cm/s |
| 155 | High | YES | NO | NO | YES | YES | YES | YES | -5.84 cm/s |
| 156 | High | NO | NO | NO | YES | YES | NO | YES | -6.84 cm/s |
| 157 | High | NO | NO | NO | YES | YES | NO | YES | -7.16 cm/s |
| 160 | High | YES | NO | YES | YES | YES | NO | YES | -6.06 cm/s |
| 161 | High | YES | NO | YES | YES | YES | YES | YES | -5.92 cm/s |
| 163 | High | YES | NO | YES | YES | YES | NO | NO | -5.94 cm/s |
| 180 | High | YES | NO | NO | YES | YES | NO | YES | -6.24 cm/s |
| 181 | High | YES | NO | NO | YES | YES | NO | YES | -6.24 cm/s |
| 182 | High | YES | NO | NO | YES | YES | NO | YES | -6.24 cm/s |
| 183 | High | YES | NO | NO | YES | YES | NO | YES | -6.20 cm/s |
| 184 | High | NO | NO | NO | YES | YES | NO | YES | -7.01 cm/s |
| 185 | High | NO | NO | NO | YES | YES | NO | YES | -6.40 cm/s |
| 186 | High | NO | NO | NO | YES | YES | NO | YES | -7.01 cm/s |
| 187 | High | NO | NO | NO | YES | YES | NO | YES | -7.01 cm/s |
| 188 | High | NO | NO | NO | YES | YES | NO | YES | -6.59 cm/s |
| 189 | High | NO | NO | NO | YES | YES | NO | NO | -7.20 cm/s |
| 190 | High | NO | NO | NO | YES | YES | NO | NO | -6.97 cm/s |
| 191 | High | NO | NO | NO | YES | YES | NO | NO | -6.97 cm/s |
| 192 | High | NO | NO | NO | YES | YES | NO | NO | -6.73 cm/s |
| 193 | High | NO | NO | NO | YES | NO | NO | YES | -7.41 cm/s |
| 194 | High | NO | NO | YES | YES | YES | NO | YES | -6.23 cm/s |
| 195 | High | NO | NO | NO | YES | YES | NO | NO | -7.30 cm/s |
| 196 | High | NO | NO | NO | YES | YES | NO | NO | -6.70 cm/s |
| 197 | High | NO | NO | NO | YES | YES | NO | NO | -6.55 cm/s |
| 198 | High | NO | YES | NO | YES | YES | YES | YES | -6.51 cm/s |
| 199 | High | NO | YES | NO | YES | NO | NO | NO | -6.98 cm/s |
| 200 | High | NO | YES | NO | YES | NO | NO | NO | -7.15 cm/s |
| 201 | High | NO | NO | NO | YES | YES | NO | YES | -6.60 cm/s |
| 202 | High | NO | NO | NO | YES | YES | NO | YES | -6.44 cm/s |
| 203 | High | NO | NO | NO | YES | YES | NO | YES | -6.44 cm/s |
| 204 | High | YES | NO | NO | YES | YES | NO | YES | -5.86 cm/s |
| 205 | High | YES | NO | NO | YES | YES | NO | YES | -5.68 cm/s |
| 206 | High | NO | NO | YES | YES | YES | NO | YES | -6.26 cm/s |
| 207 | High | NO | NO | YES | YES | YES | NO | YES | -5.99 cm/s |
| 208 | High | NO | YES | NO | YES | NO | NO | NO | -7.00 cm/s |
| 209 | High | NO | YES | NO | YES | YES | NO | YES | -6.60 cm/s |

Table S6- ADME predictions for componds by QikProp

| Entry | #stars | QPlogPo/w | QPlogS | QPPCaco | QPlogBB | #metab | QPlogKhsa | Percent Human  OralAbsorption | Rule Of  Five |
| --- | --- | --- | --- | --- | --- | --- | --- | --- | --- |
| 1 | 0 | 2.558 | -5.894 | 173.29 | -1.354 | 1 | 0.053 | 81.995 | 0 |
| 4 | 0 | 3.255 | -5.053 | 824.416 | -0.628 | 2 | 0.222 | 100 | 0 |
| 7 | 0 | 2.587 | -5.964 | 174.039 | -1.341 | 1 | 0.054 | 82.199 | 0 |
| 24 | 0 | 3.255 | -5.053 | 824.416 | -0.628 | 2 | 0.222 | 100 | 0 |
| 25 | 0 | 3.786 | -5.673 | 802.437 | -0.399 | 1 | 0.336 | 100 | 0 |
| 26 | 0 | 2.383 | -5.7 | 162.668 | -1.455 | 1 | 0.023 | 80.479 | 0 |
| 27 | 0 | 3.031 | -4.636 | 788.183 | -0.71 | 2 | 0.199 | 96.54 | 0 |
| 28 | 0 | 3.636 | -5.484 | 785.398 | -0.473 | 1 | 0.311 | 100 | 0 |
| 29 | 0 | 3.253 | -6.136 | 328.078 | -1.142 | 1 | 0.416 | 91.024 | 0 |
| 30 | 0 | 4.123 | -5.683 | 1519.519 | -0.42 | 2 | 0.576 | 100 | 0 |
| 31 | 0 | 4.622 | -6.202 | 1494.188 | -0.189 | 1 | 0.683 | 100 | 0 |
| 32 | 0 | 2.957 | -5.537 | 368.186 | -0.965 | 1 | 0.283 | 90.19 | 0 |
| 33 | 0 | 3.684 | -5.099 | 1752.41 | -0.254 | 2 | 0.443 | 100 | 0 |
| 34 | 0 | 4.272 | -5.692 | 1767.835 | -0.017 | 1 | 0.561 | 100 | 0 |
| 35 | 0 | 2.583 | -5.957 | 127.997 | -1.662 | 1 | 0.103 | 79.786 | 0 |
| 36 | 0 | 3.435 | -5.22 | 622.34 | -0.903 | 2 | 0.281 | 100 | 0 |
| 37 | 0 | 3.839 | -5.746 | 616.327 | -0.666 | 1 | 0.393 | 100 | 0 |
| 38 | 1 | 2.944 | -6.823 | 204.886 | -1.156 | 1 | 0.34 | 85.554 | 0 |
| 39 | 0 | 2.602 | -6.363 | 176.591 | -1.105 | 2 | 0.203 | 82.395 | 0 |
| 40 | 0 | 3.445 | -6.46 | 357.648 | -1.059 | 1 | 0.292 | 92.816 | 0 |
| 41 | 2 | 3.833 | -6.854 | 355.248 | -0.917 | 0 | 0.479 | 95.036 | 0 |
| 42 | 1 | 3.066 | -6.046 | 345.589 | -0.895 | 0 | 0.236 | 90.333 | 0 |
| 43A | 0 | 2.098 | -5.555 | 105.395 | -1.757 | 1 | -0.087 | 75.431 | 0 |
| 43B | 0 | 2.098 | -5.555 | 105.395 | -1.757 | 1 | -0.087 | 75.431 | 0 |
| 44 | 0 | 3.483 | -4.821 | 1051.985 | -0.53 | 2 | 0.255 | 100 | 0 |
| 46 | 0 | 3.782 | -4.74 | 853.891 | -0.337 | 1 | 0.438 | 100 | 0 |
| 47 | 1 | 4.232 | -5.353 | 861.109 | -0.09 | 0 | 0.592 | 100 | 0 |
| 109 | 0 | 3.861 | -5.709 | 827.093 | -0.363 | 1 | 0.357 | 100 | 0 |
| 110 | 0 | 3.497 | -5.426 | 825.346 | -0.556 | 2 | 0.375 | 100 | 0 |
| 111 | 0 | 3.653 | -5.205 | 866.72 | -0.625 | 2 | 0.288 | 100 | 0 |
| 112 | 1 | 4.247 | -6.26 | 824.516 | -0.292 | 1 | 0.467 | 100 | 0 |
| 113 | 1 | 4.486 | -6.391 | 904.52 | -0.283 | 2 | 0.49 | 100 | 0 |
| 114 | 0 | 4.223 | -6.047 | 849.221 | -0.397 | 2 | 0.445 | 100 | 0 |
| 115 | 0 | 3.619 | -5.958 | 400.053 | -0.962 | 1 | 0.384 | 94.708 | 0 |
| 116 | 0 | 2.258 | -5.145 | 91.879 | -1.598 | 1 | 0.004 | 75.303 | 0 |
| 117 | 0 | 3.501 | -5.735 | 837.94 | -0.58 | 2 | 0.269 | 100 | 0 |
| 118 | 0 | 2.542 | -5.202 | 243.868 | -1.142 | 1 | 0.002 | 84.557 | 0 |
| 119 | 0 | 2.778 | -5.614 | 243.544 | -1.271 | 1 | 0.132 | 85.928 | 0 |
| 120 | 0 | 2.11 | -4.702 | 179.683 | -1.36 | 1 | -0.184 | 79.653 | 0 |
| 121 | 0 | 2.524 | -5.691 | 169.038 | -1.375 | 2 | 0.045 | 81.6 | 0 |
| 122 | 0 | 3.385 | -5.073 | 853.935 | -0.602 | 3 | 0.22 | 100 | 0 |
| 123 | 0 | 3.917 | -5.843 | 876.712 | -0.332 | 2 | 0.361 | 100 | 0 |
| 124 | 0 | 3.608 | -5.464 | 826.379 | -0.526 | 2 | 0.266 | 100 | 0 |
| 125 | 0 | 3.533 | -5.281 | 859.726 | -0.538 | 2 | 0.249 | 100 | 0 |
| 126 | 1 | 4.079 | -5.945 | 965.044 | -0.209 | 1 | 0.384 | 100 | 0 |
| 127 | 2 | 4.428 | -6.383 | 957.404 | -0.398 | 3 | 0.456 | 100 | 0 |
| 128 | 0 | 3.546 | -5.18 | 977.431 | -0.676 | 4 | 0.2 | 100 | 0 |
| 129 | 0 | 2.265 | -4.155 | 516.562 | -0.689 | 3 | -0.134 | 88.766 | 0 |
| 130 | 0 | 3.227 | -5.303 | 531.453 | -0.551 | 2 | 0.137 | 94.624 | 0 |
| 131 | 0 | 3.348 | -5.354 | 666.723 | -0.457 | 2 | 0.154 | 100 | 0 |
| 132 | 0 | 2.487 | -5.835 | 179.391 | -1.244 | 2 | -0.013 | 81.846 | 0 |
| 133 | 0 | 4.301 | -5.91 | 1228.209 | -0.226 | 1 | 0.438 | 100 | 0 |
| 134 | 0 | 2.707 | -5.754 | 191.226 | -1.34 | 1 | 0.075 | 83.633 | 0 |
| 135 | 0 | 4.624 | -6.293 | 1125.231 | -0.381 | 1 | 0.554 | 100 | 0 |
| 136 | 0 | 4.591 | -6.443 | 1114.74 | -0.328 | 1 | 0.587 | 100 | 0 |
| 137 | 1 | 4.491 | -6.448 | 932.656 | -0.078 | 1 | 0.454 | 100 | 0 |
| 138 | 0 | 3.957 | -5.899 | 769.415 | -0.378 | 2 | 0.407 | 100 | 0 |
| 139 | 0 | 2.034 | -5.149 | 126.229 | -1.672 | 1 | -0.166 | 76.46 | 0 |
| 140 | 0 | 2.018 | -4.922 | 148.261 | -1.575 | 3 | -0.179 | 77.617 | 0 |
| 141 | 0 | 2.415 | -4.723 | 206.53 | -1.228 | 2 | 0.055 | 82.521 | 0 |
| 142 | 0 | 1.877 | -5.244 | 104.324 | -1.589 | 2 | -0.175 | 74.062 | 0 |
| 143 | 0 | 2.008 | -5.51 | 134.782 | -1.486 | 2 | -0.162 | 76.817 | 0 |
| 149 | 1 | 4.141 | -5.329 | 1269.875 | -0.15 | 2 | 0.294 | 100 | 0 |
| 151 | 0 | 4.208 | -5.694 | 1515.167 | -0.34 | 2 | 0.518 | 100 | 0 |
| 155 | 0 | 3.813 | -4.506 | 1837.6 | 0.231 | 1 | -0.022 | 100 | 0 |
| 156 | 0 | 2.847 | -5.445 | 309.198 | -1.07 | 1 | -0.104 | 88.187 | 0 |
| 157 | 0 | 2.849 | -5.68 | 214.335 | -1.266 | 1 | -0.076 | 85.349 | 0 |
| 160 | 1 | 3.046 | -5.318 | 638.336 | -0.745 | 1 | -0.072 | 94.984 | 0 |
| 161 | 0 | 4.046 | -4.876 | 3174.857 | 0.007 | 2 | 0.162 | 100 | 0 |
| 163 | 0 | 3.253 | -5.665 | 567.786 | -0.791 | 1 | 0.053 | 95.288 | 0 |
| 180 | 0 | 4.09 | -5.893 | 802.307 | -0.49 | 2 | 0.432 | 100 | 0 |
| 181 | 0 | 4.06 | -5.849 | 799.707 | -0.485 | 2 | 0.418 | 100 | 0 |
| 182 | 0 | 4 | -5.69 | 827.049 | -0.513 | 2 | 0.415 | 100 | 0 |
| 183 | 0 | 3.749 | -5.524 | 789.364 | -0.602 | 2 | 0.389 | 100 | 0 |
| 184 | 0 | 2.23 | -5.533 | 163.33 | -1.66 | 2 | -0.021 | 79.613 | 0 |
| 185 | 0 | 3.926 | -5.724 | 807.911 | -0.679 | 3 | 0.383 | 100 | 0 |
| 186 | 0 | 2.221 | -5.525 | 161.786 | -1.664 | 2 | -0.023 | 79.488 | 0 |
| 187 | 0 | 2.304 | -5.608 | 179.41 | -1.629 | 2 | -0.008 | 80.777 | 0 |
| 188 | 1 | 3.109 | -6.741 | 160.077 | -1.357 | 2 | 0.227 | 84.605 | 0 |
| 189 | 0 | 1.43 | -5.445 | 19.81 | -2.662 | 2 | -0.082 | 58.531 | 0 |
| 190 | 0 | 2.902 | -4.861 | 424.989 | -0.875 | 4 | 0.058 | 90.983 | 0 |
| 191 | 0 | 2.876 | -4.787 | 433.199 | -0.858 | 4 | 0.047 | 90.977 | 0 |
| 192 | 0 | 3.271 | -5.088 | 644.212 | -0.692 | 3 | 0.156 | 96.377 | 0 |
| 193 | 0 | 2.041 | -5.561 | 142.888 | -1.437 | 2 | -0.165 | 77.468 | 0 |
| 194 | 0 | 3.888 | -5.415 | 782.522 | -0.477 | 3 | 0.303 | 100 | 0 |
| 195 | 0 | 2.065 | -4.689 | 112.934 | -1.588 | 2 | -0.404 | 75.78 | 0 |
| 196 | 0 | 2.938 | -4.885 | 540.814 | -0.644 | 3 | -0.012 | 93.065 | 0 |
| 197 | 0 | 2.222 | -5.406 | 179.391 | -1.493 | 2 | 0.097 | 80.297 | 0 |
| 198 | 0 | 2.999 | -6.264 | 241.309 | -1.354 | 1 | 0.385 | 87.151 | 0 |
| 199 | 0 | 2.852 | -4.68 | 602.34 | -0.519 | 2 | 0.011 | 93.396 | 0 |
| 200 | 0 | 2.532 | -4.558 | 521.061 | -0.564 | 2 | -0.105 | 90.395 | 0 |
| 201 | 0 | 3.394 | -5.133 | 786.613 | -0.573 | 2 | 0.193 | 100 | 0 |
| 202 | 0 | 4.181 | -6.233 | 785.971 | -0.607 | 3 | 0.429 | 100 | 0 |
| 203 | 0 | 4.084 | -5.936 | 801.304 | -0.626 | 3 | 0.418 | 100 | 0 |
| 204 | 0 | 4.475 | -6.488 | 1072.686 | -0.507 | 4 | 0.655 | 100 | 0 |
| 205 | 1 | 4.718 | -6.801 | 984.038 | -0.54 | 5 | 0.784 | 100 | 0 |
| 206 | 0 | 4.061 | -6.116 | 762.006 | -0.532 | 4 | 0.461 | 100 | 0 |
| 207 | 2 | 4.271 | -6.214 | 761.485 | -0.335 | 3 | 0.426 | 100 | 0 |
| 208 | 0 | 3.354 | -5.315 | 380.987 | -0.918 | 2 | 0.356 | 92.779 | 0 |
| 209 | 1 | 4.427 | -6.613 | 630.844 | -0.76 | 4 | 0.757 | 100 | 0 |

*Recommended ranges are tabulated in supplementary information.


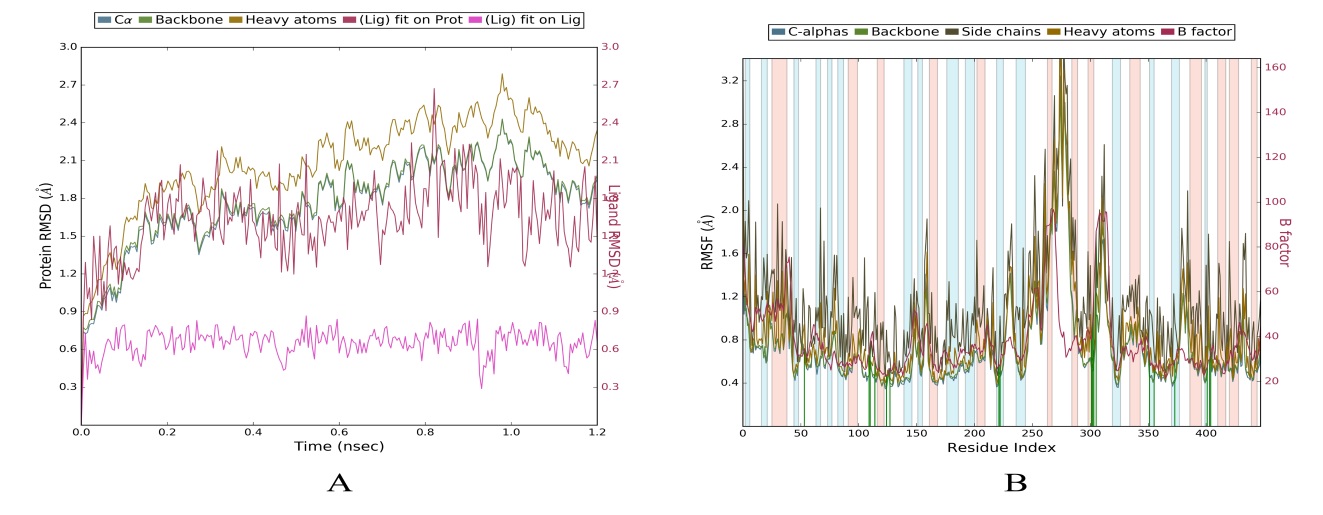


Figure S5- A] Protein-Ligand RMSD and B] Protein-RMSF plot indicating local changes along protein chains (Complex with 151).

Table S7- Recommended ranges of QikProp properties.

| Property or  Descriptor | Description | Range or  recommended  values |
| --- | --- | --- |
| #stars | Number of property or descriptor values that fall outside the 95% range of similar values for known drugs. Outlying  descriptors and predicted properties are denoted with asterisks (*) in the .out file. A large number of stars suggests that a molecule is less drug-like than molecules with few stars. The following properties and descriptors are included in the determination of #stars: MW, dipole, IP, EA, SASA, FOSA, FISA, PISA, WPSA, PSA, volume, #rotor, donorHB, accptHB, glob, QPpolrz, QPlogPC16, QPlogPoct, QPlogPw, QPlogPo/w,  logS, QPLogKhsa, QPlogBB, #metabol | 0 – 5 |
| QPlogPo/w | Predicted octanol/water partition coefficient | –2.0 – 6.5 |
| QPlogS | Predicted aqueous solubility, log S. S in mol dm–3 is the concentration of the solute in a saturated solution that is in equilibrium with the crystalline solid. | –6.5 – 0.5 |
| QPPCaco | Predicted apparent Caco-2 cell permeability in nm/sec. Caco-  2 cells are a model for the gut-blood barrier. QikProp predictions are for non-active transport. | <25 poor,  >500 great |
| QPlogBB | Predicted brain/blood partition coefficient. Note: QikProp predictions are for orally delivered drugs so, for example, dopamine and serotonin are CNS negative because they are too polar to cross the blood-brain barrier | –3.0 – 1.2 |
| #metab | Number of likely metabolic reactions | 1 – 8 |
| QPlogKhsa | Prediction of binding to human serum albumin. | –1.5 – 1.5 |
| PercentHuman-  OralAbsorption | Predicted human oral absorption on 0 to 100% scale. The prediction is based on a quantitative multiple linear regression  model. This property usually correlates well with HumanOral-  Absorption, as both measure the same property. | >80% is high  <25% is poor |
| RuleOfFive | Number of violations of Lipinski’s rule of five . The rules  are: mol_MW < 500, QPlogPo/w < 5, donorHB ≤ 5, accptHB  ≤ 10. Compounds that satisfy these rules are considered druglike.  (The “five” refers to the limits, which are multiples of 5.) | maximum is 4 |





Figure S6-Ligand based virtual screening using SwissSimilarity.

Table S8- In-silico calculation of Drug-likeness and medicinal Chemistry for top 9 ASINEX molecules by SwissADME.

| COMP.ID | Lipinski | Ghose | Veber | Egan | Muegge | Bioavailability Score |
| --- | --- | --- | --- | --- | --- | --- |
| ZINC19500480 | Yes; 0 violation | Yes; 0 violation | Yes; 0 violation | Yes; 0 violation | Yes; 0 violation | 0.55 |
| ZINC19500487 | Yes; 0 violation | No; 1 violation: WLOGP<-0.4 | Yes; 0 violation | Yes; 0 violation | Yes; 0 violation | 0.55 |
| ZINC07426404 | Yes; 0 violation | Yes; 0 violation | Yes; 0 violation | Yes; 0 violation | Yes; 0 violation | 0.55 |
| ZINC02252037 | Yes; 0 violation | Yes; 0 violation | Yes; 0 violation | Yes; 0 violation | Yes; 0 violation | 0.55 |
| ZINC19500488 | Yes; 0 violation | No; 1 violation: WLOGP<-0.4 | Yes; 0 violation | Yes; 0 violation | Yes; 0 violation | 0.55 |
| ZINC12196803 | Yes; 0 violation | Yes; 0 violation | Yes; 0 violation | Yes; 0 violation | Yes; 0 violation | 0.55 |
| ZINC19447057 | Yes; 0 violation | Yes; 0 violation | Yes; 0 violation | Yes; 0 violation | Yes; 0 violation | 0.55 |
| ZINC03157457 | Yes; 0 violation | Yes; 0 violation | Yes; 0 violation | Yes; 0 violation | Yes; 0 violation | 0.55 |
| ZINC03153031 | Yes; 0 violation | Yes; 0 violation | Yes; 0 violation | Yes; 0 violation | Yes; 0 violation | 0.55 |
| Medicinal Chemistry | | | | | | |
| COMP.ID | PAINS | Brenk | Leadlikeness | Synthetic accessibility | | |
| ZINC19500480 | 0 alert | 1 alert: hydantoin | Yes | 2.75 | | |
| ZINC19500487 | 0 alert | 0 alert | No; 1 violation: MW>350 | 3.60 | | |
| ZINC07426404 | 0 alert | 1 alert: hydantoin | No; 1 violation: MW>350 | 2.99 | | |
| ZINC02252037 | 0 alert | 1 alert: hydantoin | Yes | 2.58 | | |
| ZINC19500488 | 0 alert | 0 alert | No; 1 violation: MW>350 | 3.60 | | |
| ZINC12196803 | 0 alert | 1 alert: hydantoin | Yes | 3.31 | | |
| ZINC19447057 | 0 alert | 1 alert: hydantoin | Yes | 2.56 | | |
| ZINC03157457 | 0 alert | 1 alert: hydantoin | Yes | 2.42 | | |
| ZINC03153031 | 0 alert | 1 alert: hydantoin | Yes | 2.72 | | |

Table S9- In-silico calculation of water solubility for top 9 ASINEX molecules by SwissADME.

| COMP.ID | Log S (ESOL) | Solubility | Class | Log S (Ali) | Solubility | Class | Log S (SILICOS-IT) | Solubility | Class |
| --- | --- | --- | --- | --- | --- | --- | --- | --- | --- |
| ZINC19500480 | -2.56 | 9.27e-01 mg/ml ; 2.78e-03 mol/l | Soluble | -2.92 | 4.02e-01 mg/ml ; 1.20e-03 mol/l | Soluble | -4.50 | 1.05e-02 mg/ml ; 3.16e-05 mol/l | Moderately soluble |
| ZINC19500487 | -2.29 | 1.92e+00 mg/ml ; 5.10e-03 mol/l | Soluble | -2.23 | 2.24e+00 mg/ml ; 5.96e-03 mol/l | Soluble | -2.97 | 4.05e-01 mg/ml ; 1.08e-03 mol/l | Soluble |
| ZINC07426404 | -3.46 | 1.31e-01 mg/ml ; 3.43e-04 mol/l | Soluble | -3.71 | 7.47e-02 mg/ml ; 1.96e-04 mol/l | Soluble | -6.19 | 2.45e-04 mg/ml ; 6.43e-07 mol/l | Poorly soluble |
| ZINC02252037 | -2.84 | 4.02e-01 mg/ml ; 1.45e-03 mol/l | Soluble | -3.08 | 2.32e-01 mg/ml ; 8.40e-04 mol/l | Soluble | -4.50 | 8.80e-03 mg/ml ; 3.18e-05 mol/l | Moderately soluble |
| ZINC19500488 | -2.29 | 1.92e+00 mg/ml ; 5.10e-03 mol/l | Soluble | -2.23 | 2.24e+00 mg/ml ; 5.96e-03 mol/l | Soluble | -2.97 | 4.05e-01 mg/ml ; 1.08e-03 mol/l | Soluble |
| ZINC12196803 | -2.87 | 4.70e-01 mg/ml ; 1.35e-03 mol/l | Soluble | -3.30 | 1.73e-01 mg/ml ; 4.98e-04 mol/l | Soluble | -4.52 | 1.04e-02 mg/ml ; 3.00e-05 mol/l | Moderately soluble |
| ZINC19447057 | -3.06 | 2.31e-01 mg/ml ; 8.75e-04 mol/l | Soluble | -3.13 | 1.95e-01 mg/ml ; 7.39e-04 mol/l | Soluble | -4.28 | 1.39e-02 mg/ml ; 5.27e-05 mol/l | Moderately soluble |
| ZINC03157457 | -2.60 | 6.57e-01 mg/ml ; 2.50e-03 mol/l | Soluble | -2.70 | 5.20e-01 mg/ml ; 1.98e-03 mol/l | Soluble | -4.10 | 2.10e-02 mg/ml ; 8.02e-05 mol/l | Moderately soluble |
| ZINC03153031 | -3.19 | 1.88e-01 mg/ml ; 6.48e-04 mol/l | Soluble | -3.64 | 6.71e-02 mg/ml ; 2.31e-04 mol/l | Soluble | -4.90 | 3.69e-03 mg/ml ; 1.27e-05 mol/l | Moderately soluble |

Table S10- In-silico pharmacokinetic profile of top 9 ASINEX molecules as obtained from SwissADME server.

| COMP.ID | BBB permeant | P-gp substrate | CYP1A2 inhibitor | CYP2C19 inhibitor | CYP2C9 inhibitor | CYP2D6 inhibitor | CYP3A4 inhibitor |
| --- | --- | --- | --- | --- | --- | --- | --- |
| ZINC19500480 | No | yes | No | No | No | No | No |
| ZINC19500487 | No | yes | No | No | No | No | No |
| ZINC07426404 | No | yes | No | No | yes | No | yes |
| ZINC02252037 | yes | No | No | yes | No | No | No |
| ZINC19500488 | No | yes | No | No | No | No | No |
| ZINC12196803 | No | yes | No | No | No | No | No |
| ZINC19447057 | yes | No | No | yes | No | No | No |
| ZINC03157457 | yes | No | No | yes | No | No | No |
| ZINC03153031 | yes | No | No | yes | No | No | No |


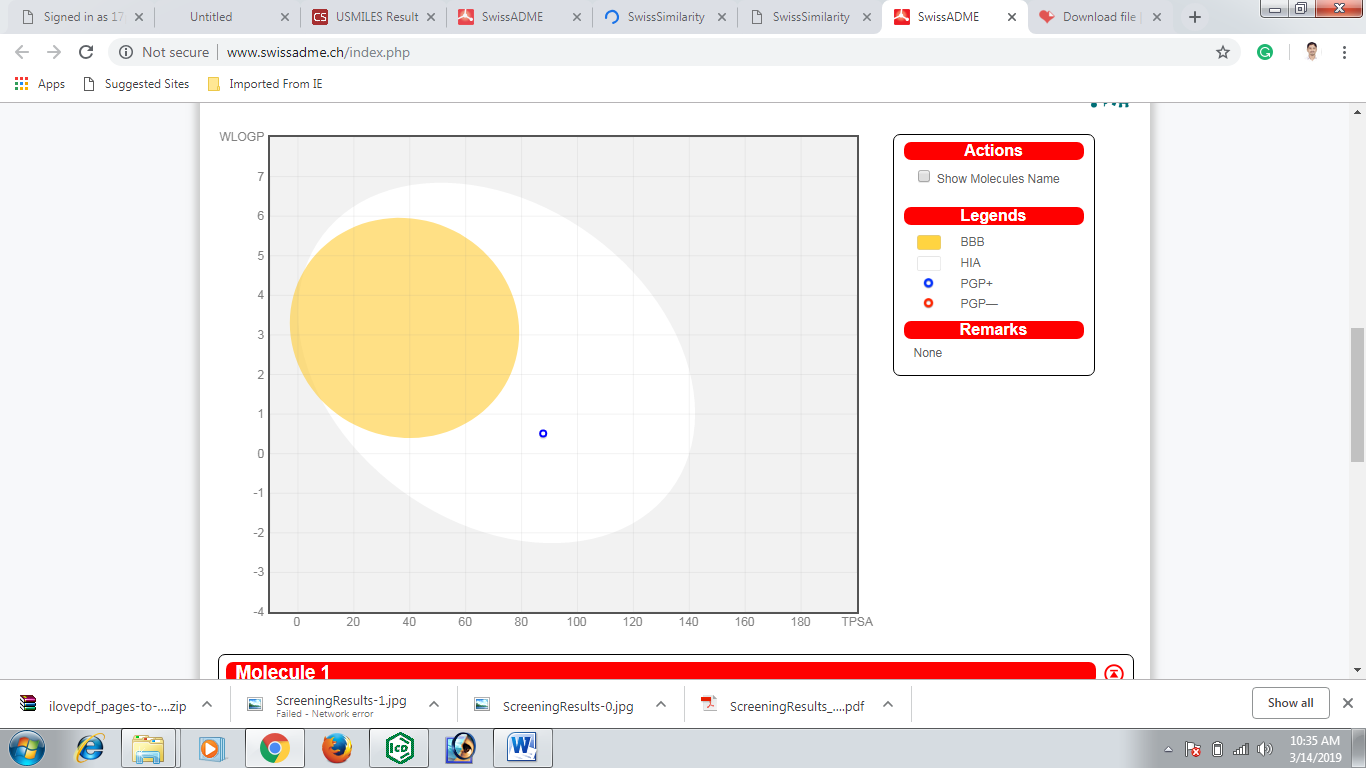

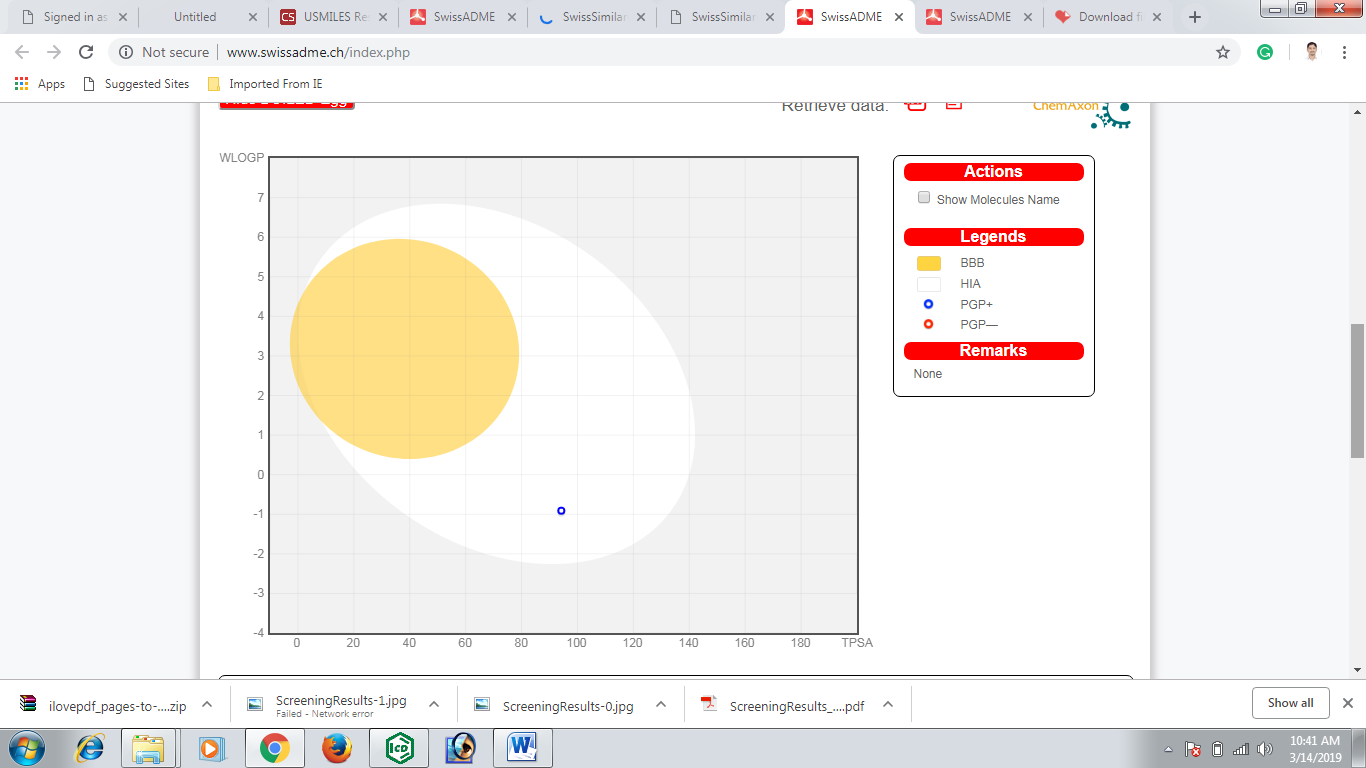

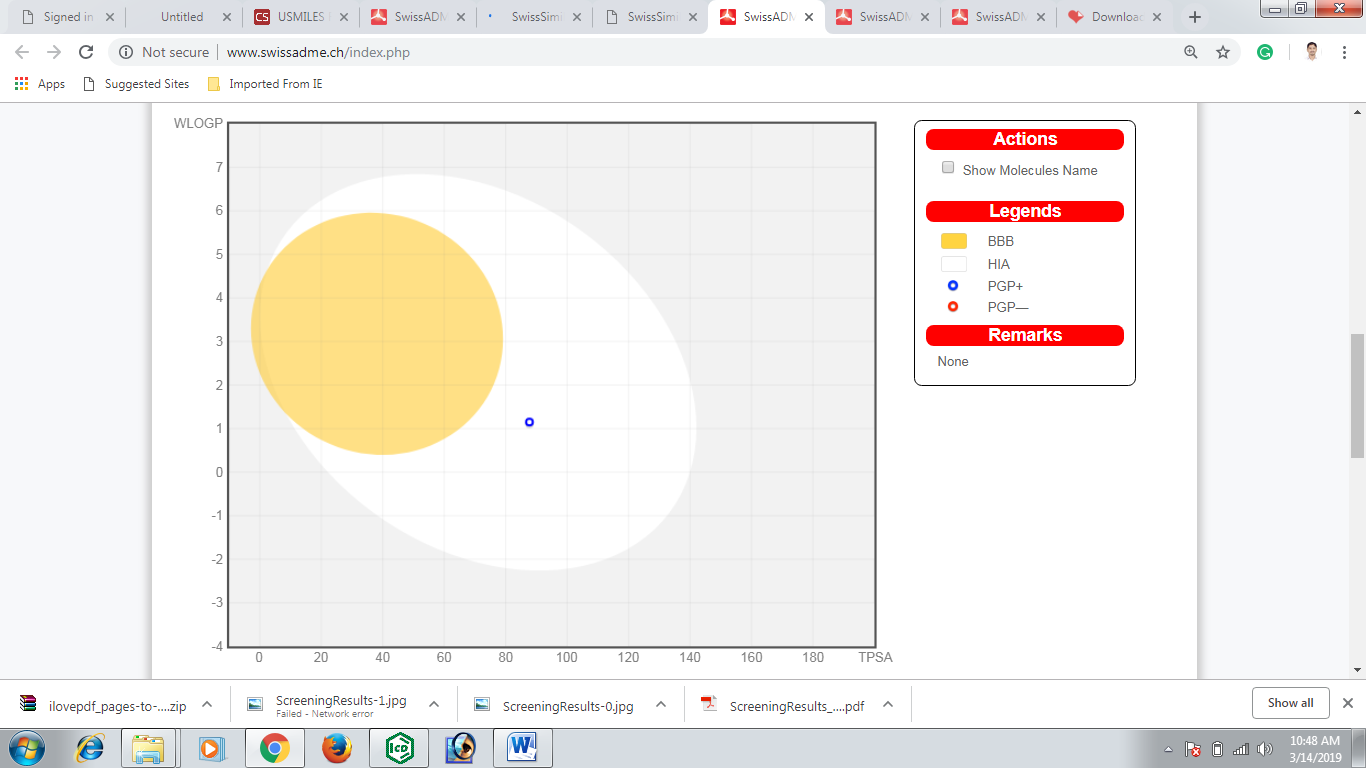


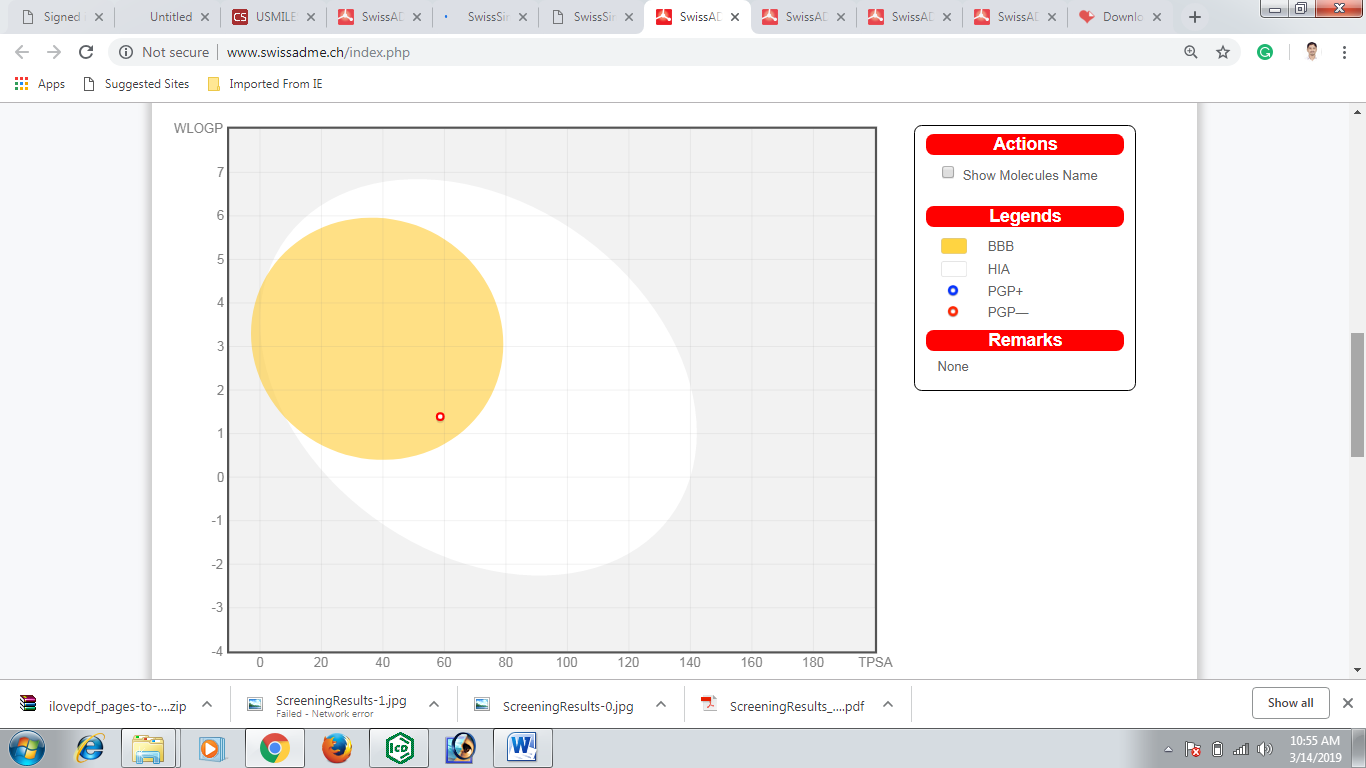

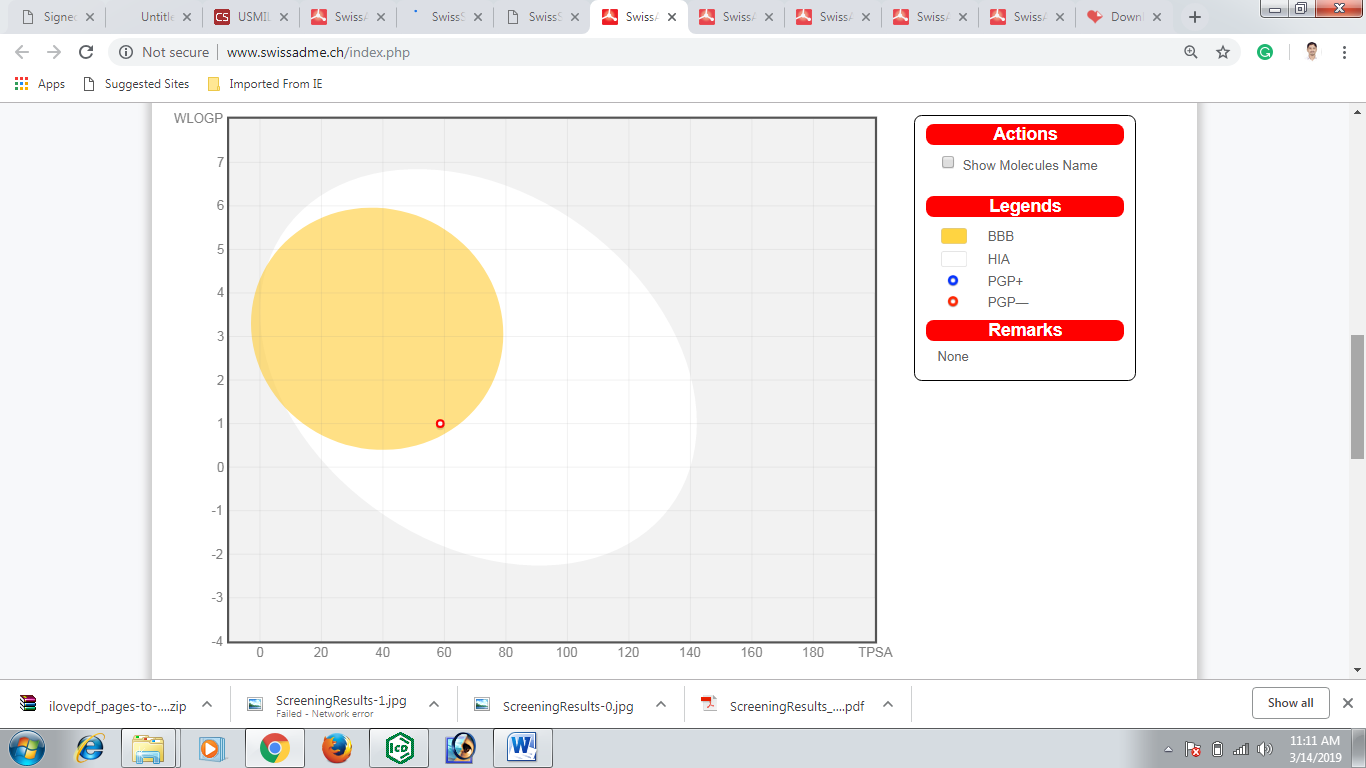

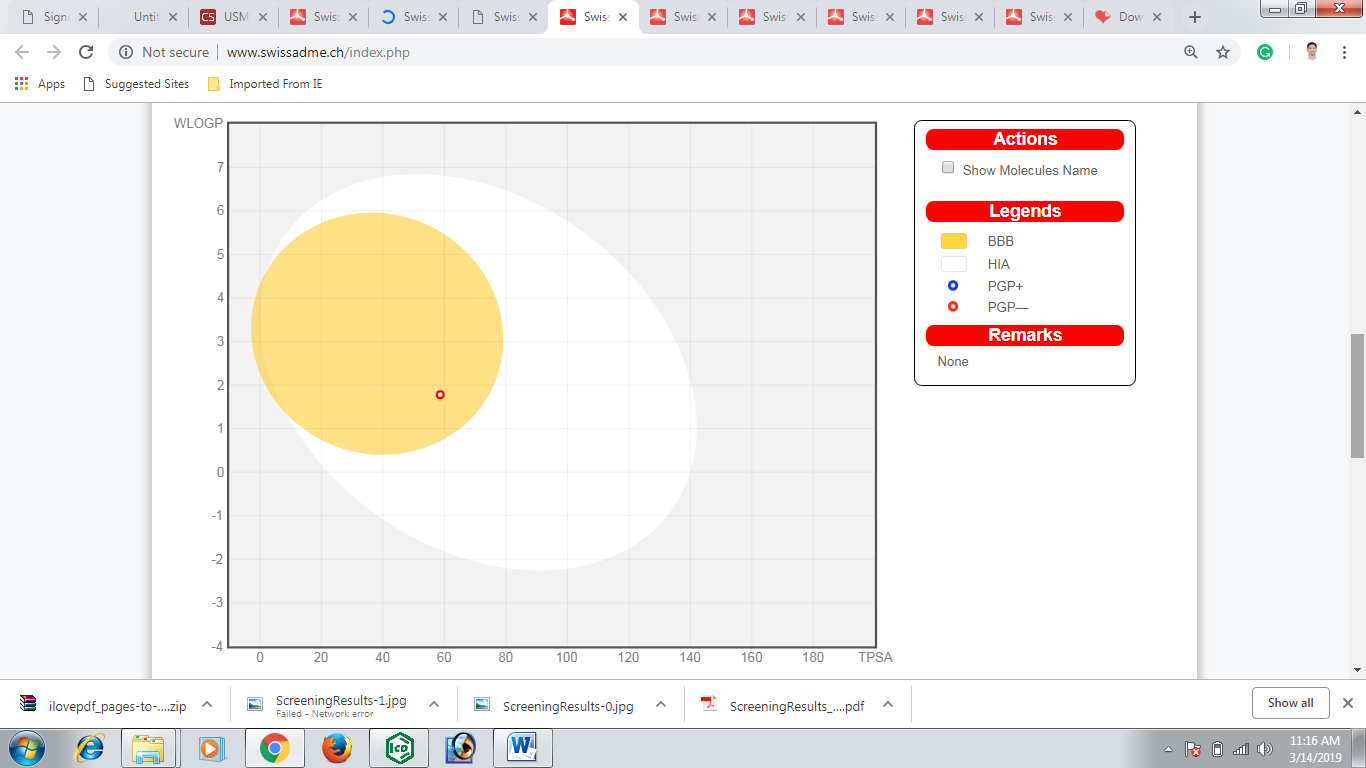


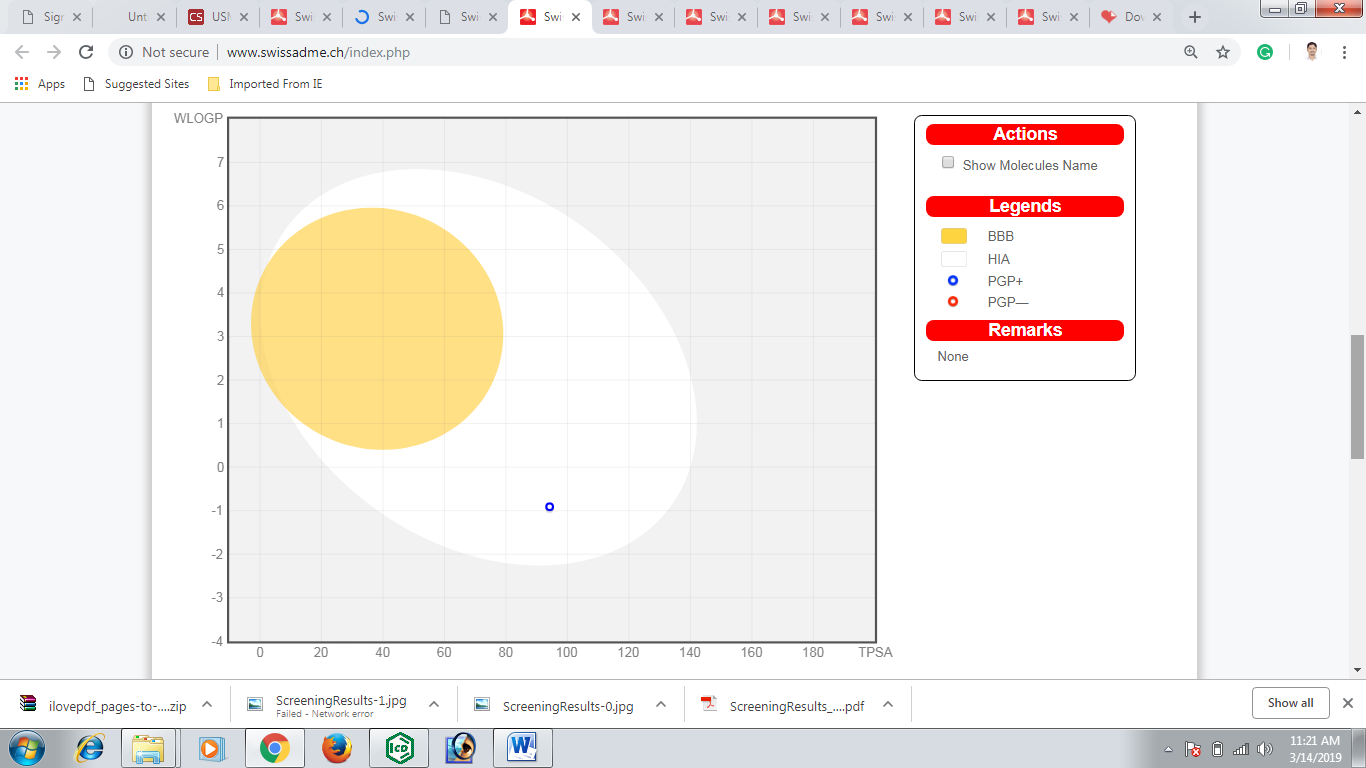

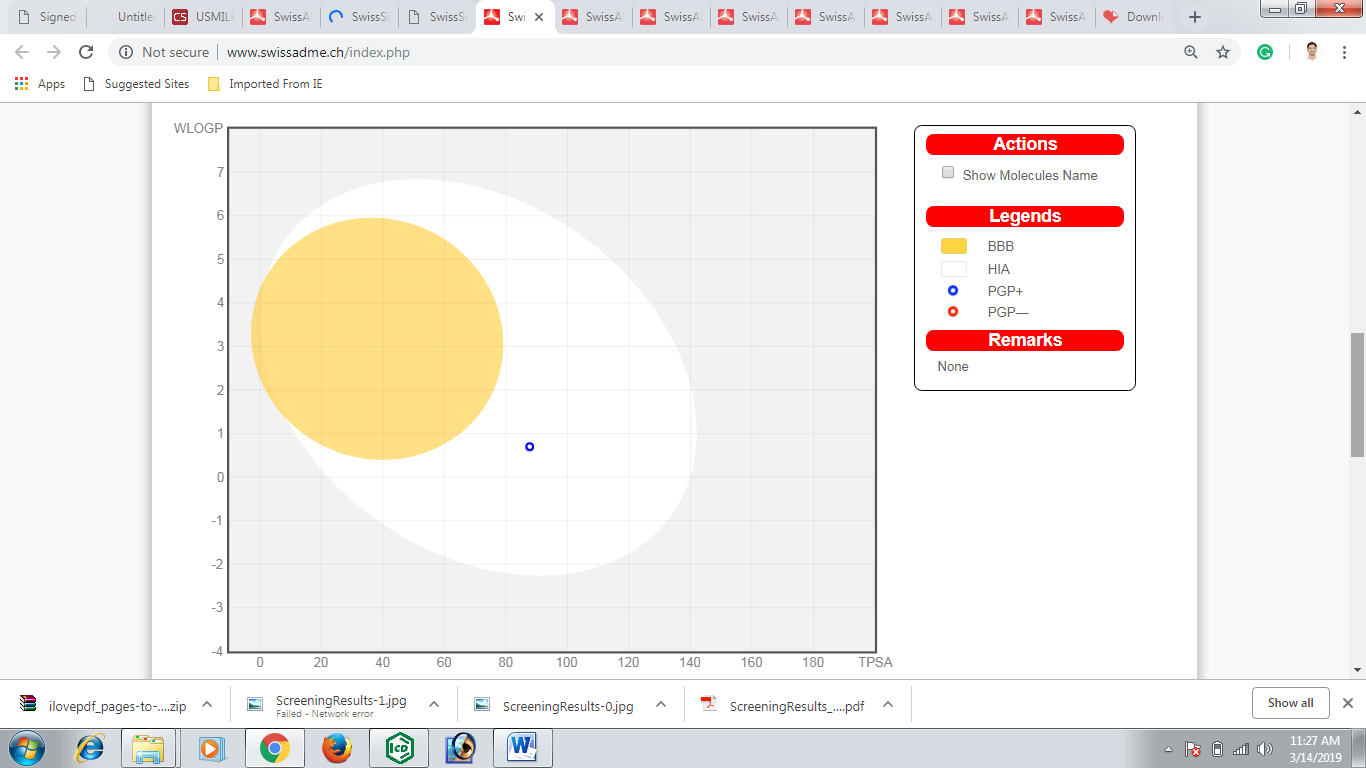

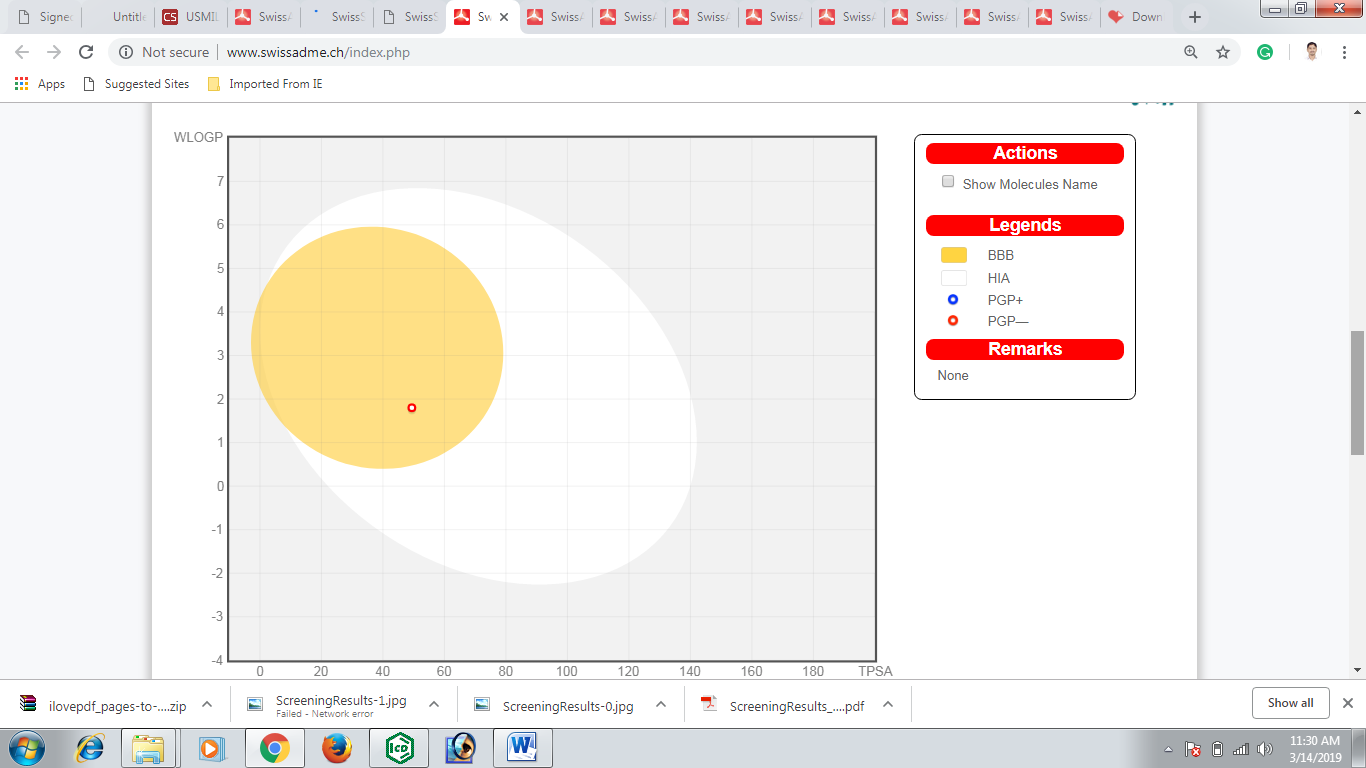


Figure S7-Boiled egg models for top 9 ASINEX HITS obtained for most docked molecule 151,(A---I) i.e. ZINC19500480,ZINC19500487,ZINC07426404,ZINC02252037,ZINC03157457,ZINC03153031, ZINC19500488,ZINC12196803 and ZINC19447057 respectively.


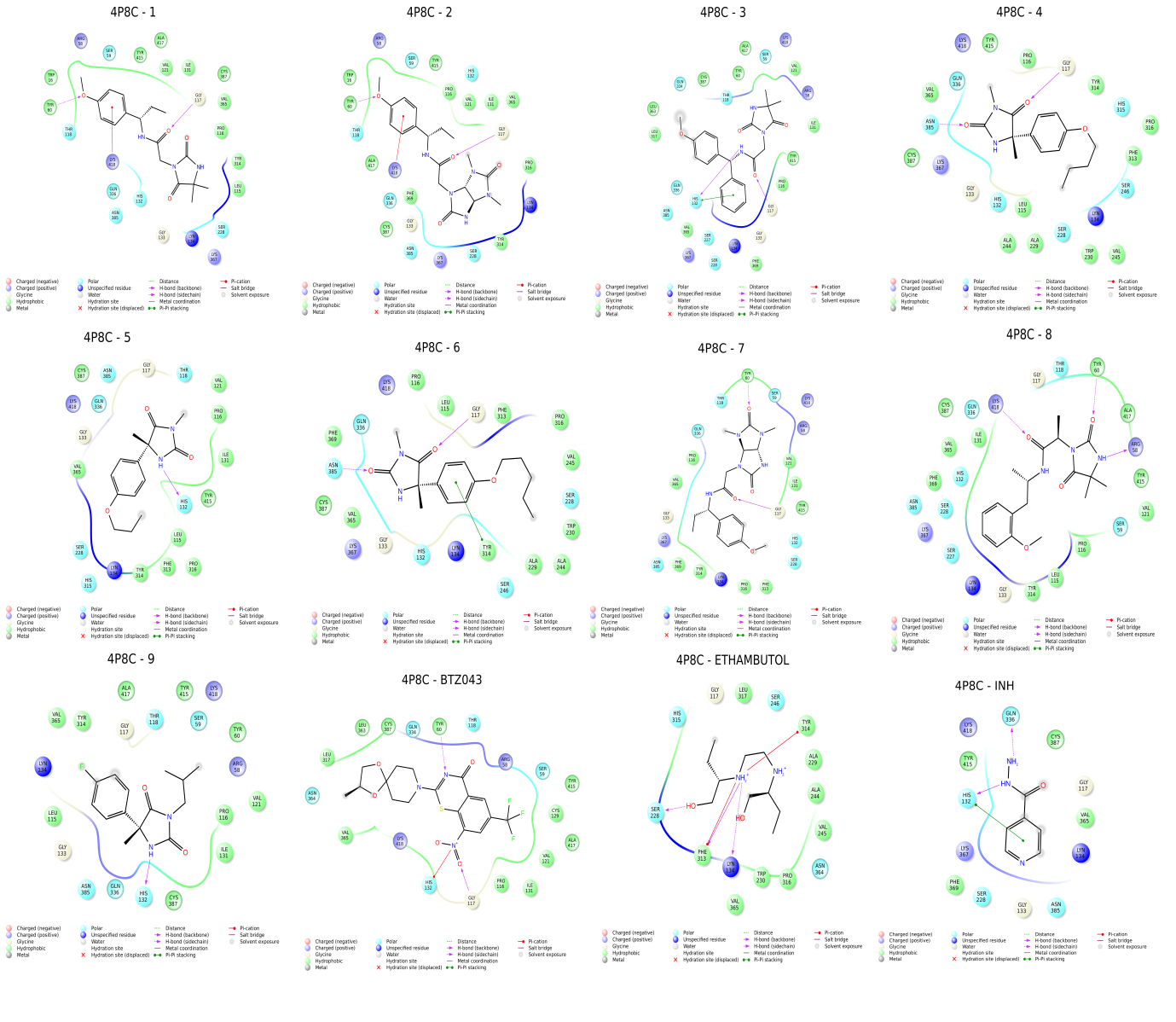


Figure S8- Molecular docking for top 9 ASINEX HITS –for 4P8C(1---9), i.e. ZINC19500480,ZINC19500487,ZINC07426404,ZINC02252037,ZINC03157457,ZINC03153031, ZINC19500488,ZINC12196803, ZINC19447057 and (BTZ043, ETHAMBUTOL, INH) respectively. [Figure 8 has been visulized using the ‘Glide’ Schrodinger, LLC, NY. V. 2020. Available at: <https://www.schrodinger.com/> ]


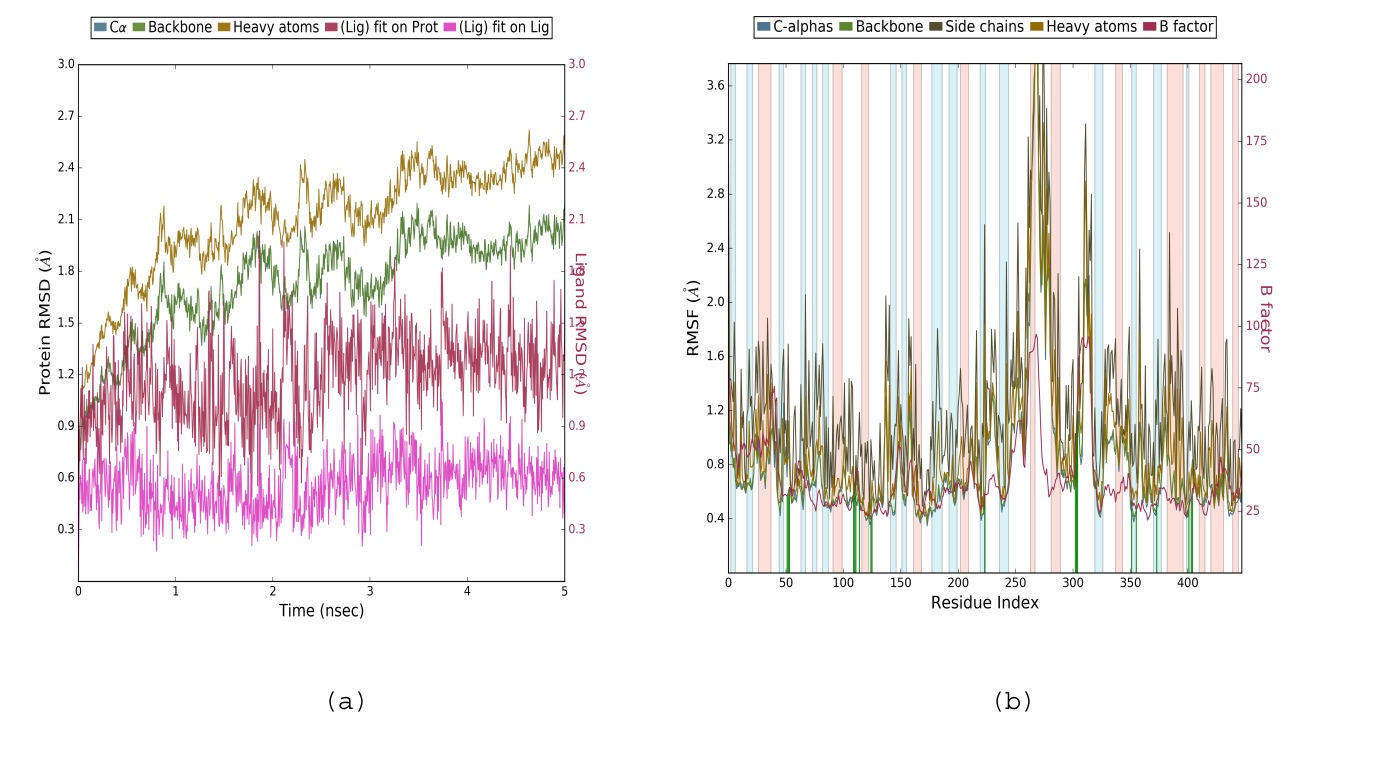


Figure S9- A] Protein-Ligand RMSD (5000ps) and B] Protein-RMSF plot indicating local changes along protein chains (best dock hit with 4p8c).


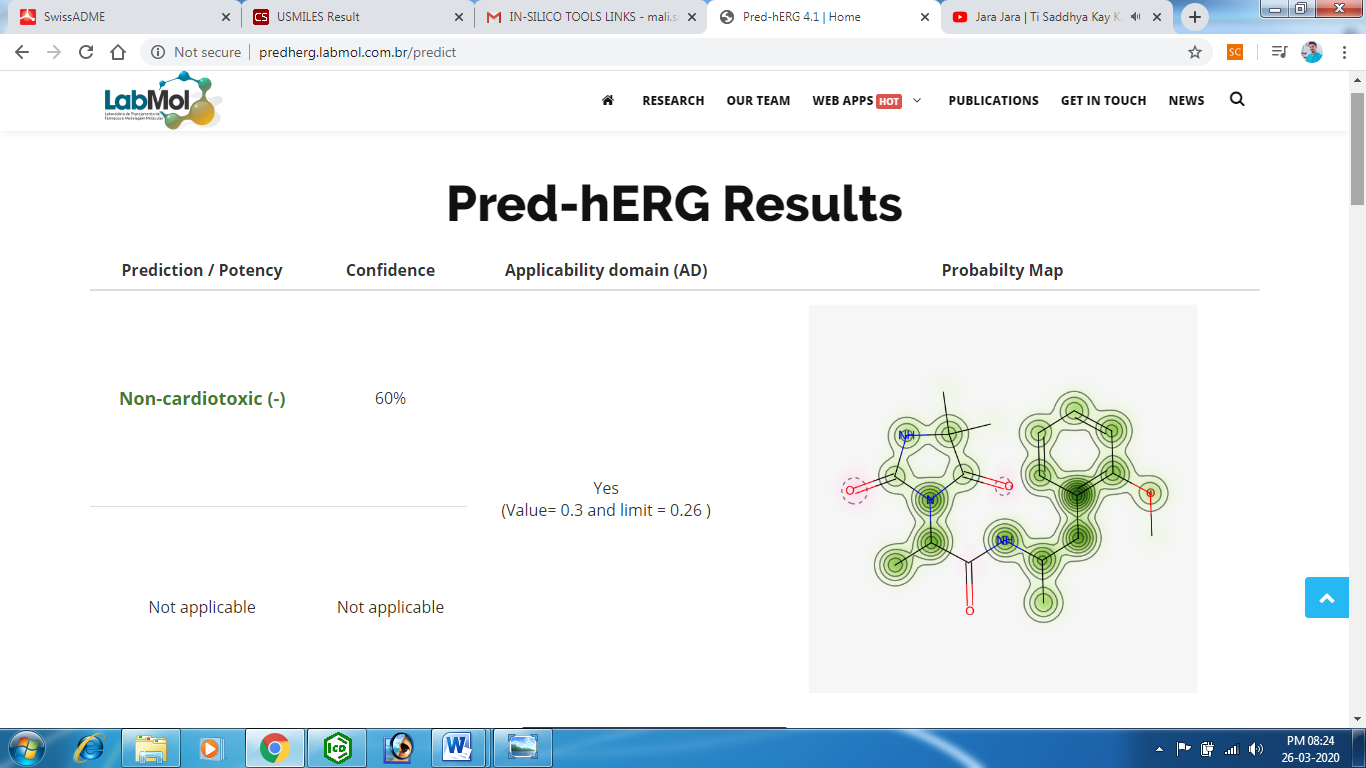


Figure S10- Pred-hERG results for best docked hit ZINC12196803.

Table S11. admetSAR toxicity predictions for top 9 ASINEX HITS –for (1---9), i.e. ZINC19500480,ZINC19500487,ZINC07426404,ZINC02252037,ZINC03157457,ZINC03153031, ZINC19500488,ZINC12196803, ZINC19447057

| Property/Comp. | 1 | 2 | 3 | 4 | 5 | 6 | 7 | 8 | 9 |
| --- | --- | --- | --- | --- | --- | --- | --- | --- | --- |
| Eye corrosion | - | - | - | - | - | - | - | - | - |
| Eye irritation | - | - | - | - | - | - | - | - | - |
| Ames mutagenesis | - | + | - | - | - | - | + | - | - |
| crustacea aquatic toxicity | - | - | - | + | - | + | - | - | - |
| Fish aquatic toxicity | - | - | + | + | - | + | - | - | + |
| Acute Oral Toxicity | 1.858  kg/mol | 2.345  kg/mol | 2.027  kg/mol | 2.007  kg/mol | 1.913  kg/mol | 2.029  kg/mol | 2.345  kg/mol | 2.18  kg/mol | 2.218  kg/mol |

**Molecular Dynamics**

The stability and binding flexibility of the selected protein-ligand docking complexes were studied in real-time using molecular dynamics simulations. To carry out MD simulations, we used Groningen Machine for Chemical Simulations (GROMACS v5.1.5) and GROMACS 96-53a6 force fields to examine the structural stability of complexes, residue, and atom behaviour. The Dundee PRODRG3.0 server was used to generate all ligand topology files. Using an explicit simple point charge (SPC) water model, the triclinic box type was constructed to overcome these MDS models. Further, the counter ions were used to neutralize the simulation box. Consequently, energy minimization equilibration using NVT and NPT was performed (parameters: Temperature (K)=300, Pressure = 1 bar, and Simulation Time = 100 ns). The molecule, best docked ZINC hit was simulated with proteins pdb id:4p8c for a period of 100 ns each.


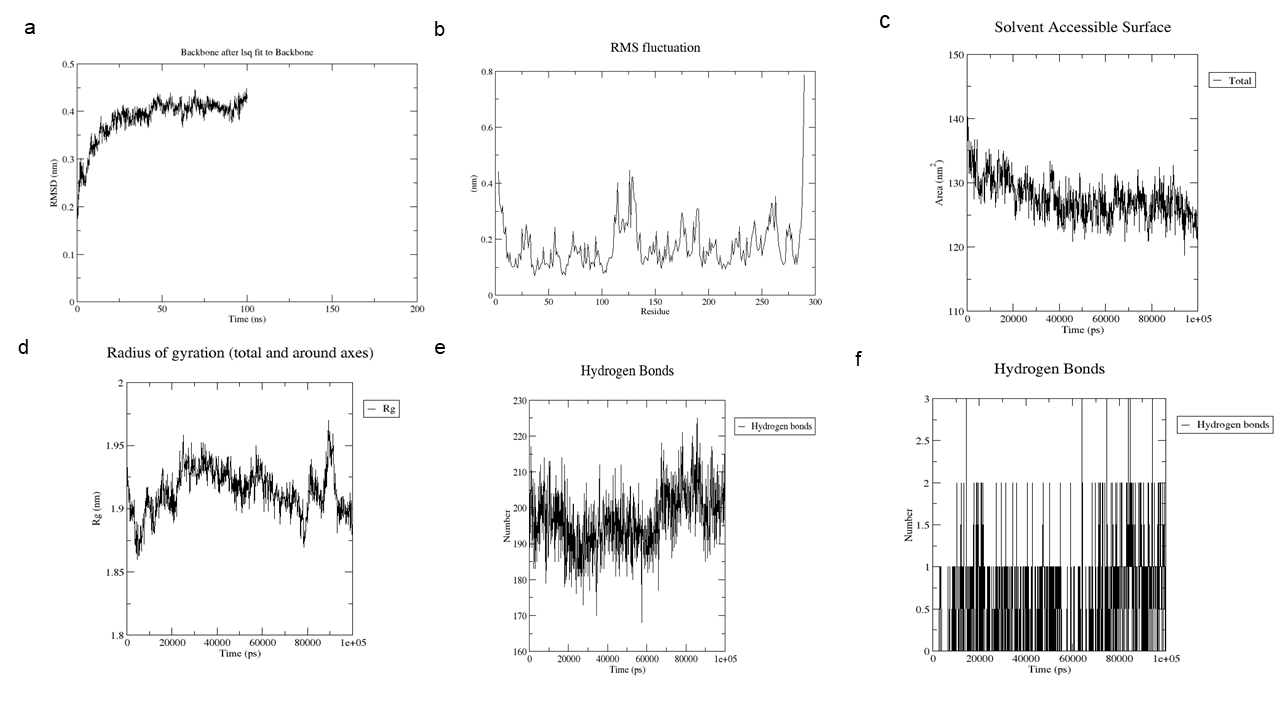


**Figure S11**- Analysis of the molecular dynamic’s simulations (a-f). (a) The root means square deviation (protein) (RMSD) for complex (best dock ZINC hit with 4p8c), (b) The root mean square fluctuations (RMSF); (c) Solvent accessible surface area ; (d) Radius of gyration; ( e) Hydrogen bonds and (f) number of H-bonds throughout simulation period of 100 ns.


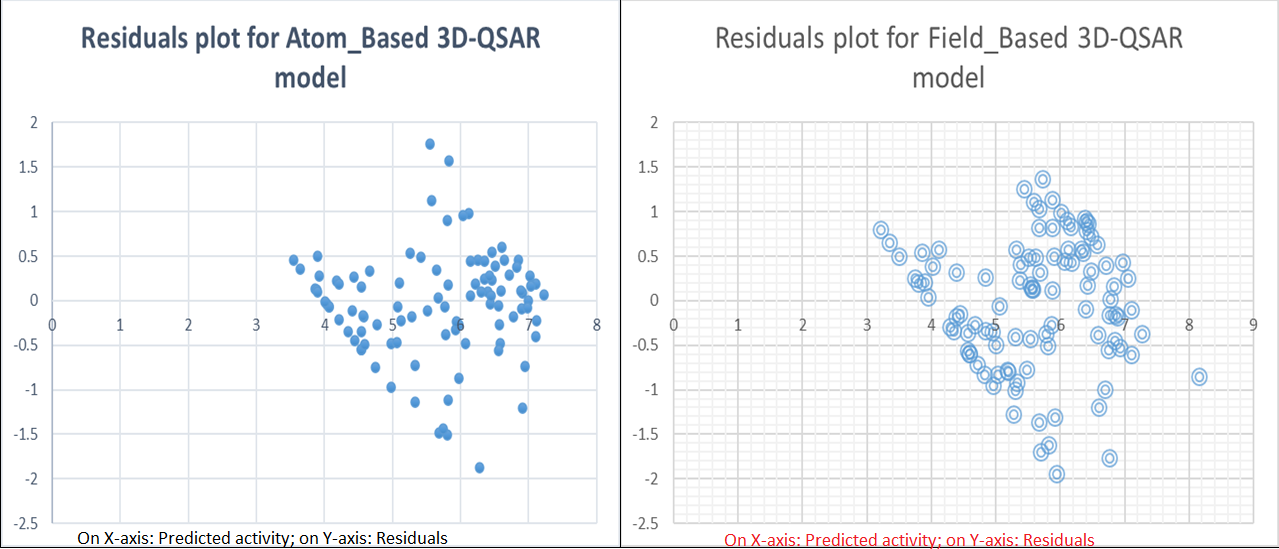


**Figure S12: Residual plots for [a] Atom based-QSAR and [b] Field-based 3D-QSAR models for best top model.**

***QSARINS GA-MLR based QSAR modelling details^1-5^***

***Structure drawing, optimization and calculations of molecular descriptors***

All structures of substituted hydantoins analogues were drawn using ChemBioDraw V.12.1. Furthermore, these 2D- structures were then converted to 3D- forms using the Chem3Dpro tool. Then, the MMFF94 force field was used for the optimization of all structures using TINKER. Open3DAlign was incorporated for alignments of the dataset molecules. Finally, PaDEL and ChemDes (a free web-based platform) were used for the calculations of molecular descriptors.

***Pruning of molecular descriptors***

It is important to note that pruning of molecular descriptors is a key step while developing QSAR models as PaDEL and ChemDes would provide more than >20,000 molecular descriptors including 1D- to 3D- for each molecule. Thus, the objective feature selection module from QSARINS ver. 2.2.2 was employed to exclude descriptors with high co-linearity (|r|>0.90) and nearly constant (> 95%) values. This avoided the inclusion of multi-collinear and spurious variables in GA-MLR models (the genetic algorithm–multi-linear regression models). At the end of the descriptor pruning step, we retained a dataset with 552 molecular descriptors covering mono-dimensional (1D-), bi-dimensional (2D-) and three-dimensional (3D-) descriptor spaces (including but not limited to fingerprint, atom-pair and other molecular descriptors). Moreover, to avoid difficulty in QSAR model interpretations, we have removed various esoteric descriptors (the descriptors for which an exact explanation is not available) in the very next step. For the QSAR model developments, we first divided our dataset randomly using the random splitting option of QSARINS into training and test sets, i.e., prediction set. The splitting of datasets into a training and test set were carried out using well-established approaches of 70%, training set :30%, prediction set. Then, these training sets were used for the building of QSAR models, while prediction sets were employed for the external validation's parameters. We had also carried out the multiple splitting of dataset molecules in such a way that, every molecule would become part of training or test set during QSAR model developments. This technique ensured that maximum number and information was gained through molecular descriptors impacting or affecting biological potential/ activity. Dataset splitting was done with 70:30 % training : test sets respectively as per known accepted procedures.

***QSAR model validation***

All our developed models were thoroughly validated using OECD principles of internal and external validations, Y-randomization and AD analysis. The statistical robustness and strength of QSAR model is based on (1) using external validation; (2) Y-randomization (or Y-scrambling); (3) internal validation based on leave-one-out (LOO) and leave-many-out (LMO) procedure (i.e., cross-validation (CV)); and (4) fulfilling of respective threshold values for the statistical parameters as follows:

r^2^_ex_ ≥ 0.6, RMSE_tr_ < RMSE_cv_, ΔK ≥ 0.05, Q^2^-F^n^ ≥ 0.60, r^2^_tr_ ≥ 0.6, Q^2^_loo_ ≥ 0.5, Q^2^_LMO_ ≥ 0.6, r^2^ > Q^2^, r^2^_m_ ≥ 0.6, CCC ≥ 0.80, (1-r^2^/r_o_^2^) < 0.1, 0.9 ≤ k ≤ 1.1 or (1-r^2^/r’_o_^2^) < 0.1, 0.9 ≤ k’ ≤ 1.1, | r_o_^2^− r’_o_^2^| < 0.3, RMSE and MAE closer to zero. It is thus, noted that any of models unable to satisfy these criteria should be omitted.

In the **Fig.S13**, yellow circles represent the predicted pIC_50_ values calculated by the models **1**. Thus, we can establish a visual trend among the experimental and predicted pIC_50_ values.

**
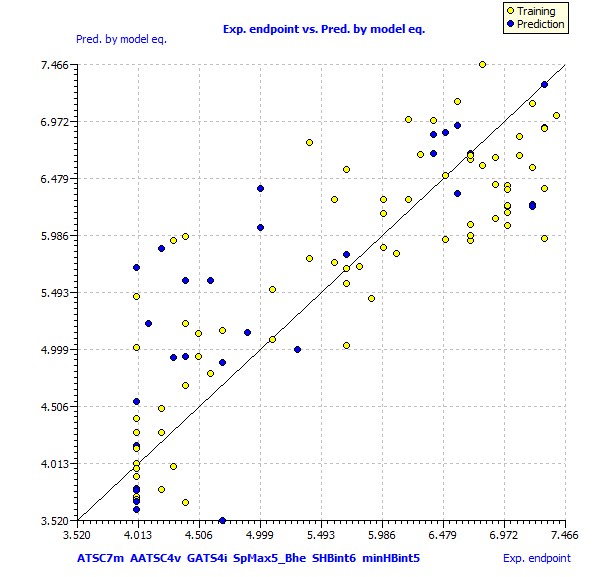

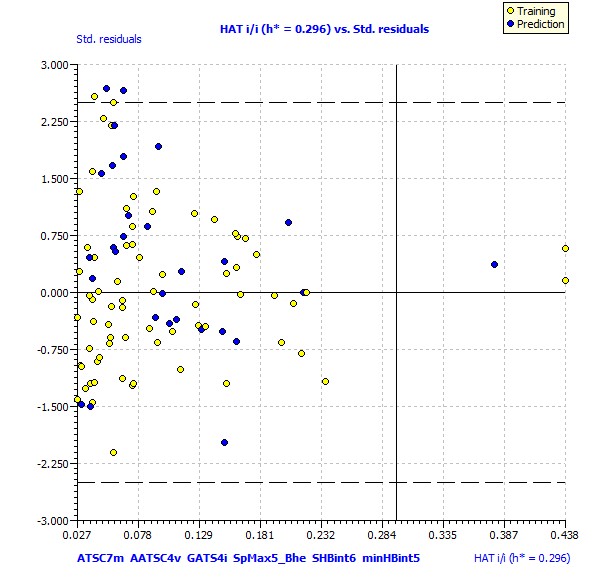
**

**
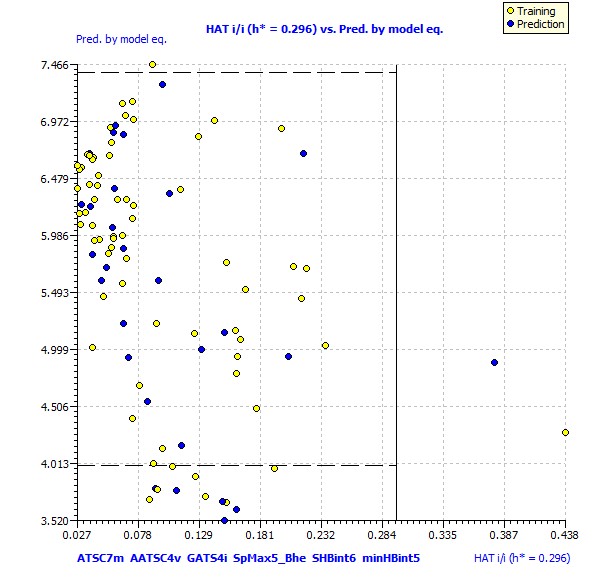

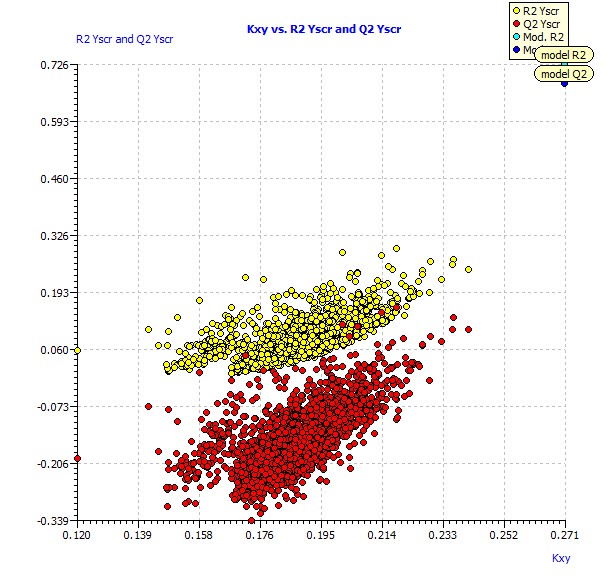
**

**Figure S13.** (a) Graph of experimental vs Predicted **pIC_50_** values for model **1** (b) William’s plot for model 1; (c) Insubria plot for model **1** (d) Y-scrambling plot for model **1.**

**Table S12:** Statistical parameters for developed QSAR models*

| **Statistical Parameter** | **Model-1** |
| --- | --- |
| **Fitting** |  |
| *R^2^_tr_* | **0.7259** |
| *R^2^_adj_.* | 0.7002 |
| *R^2^_tr_* *- R^2^_adj._* | 0.0257 |
| *LOF* | 0.5566 |
| *Kxx* | 0.1989 |
| *ΔK* | 0.0724 |
| *RMSE_tr_* | 0.6200 |
| *MAE_tr_* | 0.4883 |
| *RSS_tr_* | 27.2889 |
| *CCC_tr_* | 0.8412 |
| *s* | 0.6530 |
| *F* | 58.2538 |
| **Internal validation** | |
| *R^2^_cv_* *(Q^2^_loo_)* | **0.6819** |
| *R^2^-R^2^_cv_* | 0.0441 |
| *RMSE_cv_* | 0.6680 |
| *MAE_cv_* | 0.5336 |
| *PRESS_cv_* | 31.6777 |
| *CCC_cv_* | 0.8158 |
| *Q^2^_LMO_* | 0.6574 |
| *R^2^_Yscr_* | 0.0842 |
| *Q^2^_Yscr_* | -0.1402 |
| **External validation** | |
| *RMSE_ex_* | 0.7870 |
| *MAE_ex_* | 0.6168 |
| *PRESS_ext_* | 17.9620 |
| *R^2^_ex_* | 0.6287 |
| *Q^2^-F^1^* | 0.6254 |
| *Q^2^-F^2^* | 0.5372 |
| *Q^2^-F^3^* | 0.5583 |
| *CCC_ex_* | 0.7672 |
| *Calc. external data regr. angle from diagonal* | -7.6696° |
| *R^2^-ExPy* ***(Predictions by LOO)*** | 0.6826 |
| *R’o^2^* | 0.5669 |
| *k’* | **0.9884** |
| *r’^2^_m_* | 0.5504 |
| *R_o_^2^* | 0.6819 |
| *k* | **0.9986** |
| *r^2^_m_* | 0.6651 |

*The statistical quality and strength of a GA-MLR based QSAR model was determined on the basis of: (a) internal validation based on leave-one-out (LOO) and leave-many-out (LMO) procedure (i.e. cross-validation (CV)); (b) using External validation; (c) Y-randomization (or Y-scrambling) and (d) fulfilling of respective threshold value for the statistical parameters: ***R*^2^_tr_ ​≥ ​0.6, *Q*^2^_loo_ ​≥ ​0.5**, *Q*^2^_LMO_ ​≥ ​0.6, *R*^2^ ​> ​*Q*^2^, *R*^2^_ex_ ​≥ ​0.6, *RMSE_tr_* ​< ​*RMSE_cv_*, ΔK ​≥ ​0.05, *CCC* ​≥ ​0.80,  *r*^2^_m_ ​≥ ​0.6, (1-*r*^2^/*r*_o_^2^) ​< ​0.1, 0.9 ​≤ ​*k* ​≤ ​1.1 or (1-*r*^2^/*r*’_o_^2^) ​< ​0.1, 0.9 ​≤ ​*k*’ ​≤ ​1.1,| *r*_o_^2^− *r*’_o_^2^| ​< ​0.3 with *RMSE* and *MAE* close to zero.

Ref.

1. Mali, S.N. and Pandey, A., 2021. Molecular modeling studies on 2, 4-disubstituted imidazopyridines as anti-malarials: atom-based 3D-QSAR, molecular docking, virtual screening, in-silico ADMET and theoretical analysis. Journal of Computational Biophysics and Chemistry, 20(03), pp.267-282.

2. Kapale, S.S., Mali, S.N. and Chaudhari, H.K., 2019. Molecular modelling studies for 4-oxo-1, 4-dihydroquinoline-3-carboxamide derivatives as anticancer agents. Medicine in Drug Discovery, 2, p.100008.

3. Mali, S.N. and Pandey, A., 2022. Balanced QSAR and molecular modeling to identify structural requirements of imidazopyridine analogues as anti-infective agents against trypanosomiases. Journal of Computational Biophysics and Chemistry, 21(01), pp.83-114.

4. Gramatica, P., Chirico, N., Papa, E., Cassani, S. and Kovarich, S., 2013. QSARINS: A new software for the development, analysis, and validation of QSAR MLR models.

5. Canizares-Carmenate, Y., Mena-Ulecia, K., Perera-Sardina, Y., Torrens, F. and Castillo-Garit, J.A., 2019. An approach to identify new antihypertensive agents using Thermolysin as model: In silico study based on QSARINS and docking. Arabian Journal of Chemistry, 12(8), pp.4861-4877.

DATA IMPORTED: 13-08-2022 - 01:08:12

Source file: C:\Users\suraj\Desktop\QSARINS\Padel - QSARINS_in_CSV.csv

nAcid

apol

naAromAtom

nAromBond

nAtom

nHeavyAtom

nB

nO

nS

nP

nF

nCl

nBr

nI

ATS0m

ATS0v

ATS1v

ATS2v

ATS3v

ATS4v

ATS5v

ATS6v

ATS7v

ATS0e

ATS1e

ATS2e

ATS3e

ATS4e

ATS5e

ATS6e

ATS7e

ATS8e

ATS1p

ATS2p

ATS4p

ATS5p

ATS6p

ATS8p

ATS0i

ATS1i

ATS2i

ATS5i

ATS0s

AATS0m

AATS0v

AATS1v

AATS2v

AATS0e

AATS1p

AATS0s

ATSC4c

ATSC5c

ATSC6c

ATSC8c

ATSC1m

ATSC5m

ATSC8m

ATSC0v

ATSC1v

ATSC5v

ATSC6v

ATSC8v

ATSC0e

ATSC1e

ATSC2e

ATSC3e

ATSC4e

ATSC6e

ATSC7e

ATSC8e

ATSC1p

ATSC2p

ATSC5p

ATSC6p

ATSC7p

ATSC8p

ATSC0i

ATSC1i

ATSC4i

ATSC5i

ATSC6i

ATSC8i

ATSC0s

ATSC1s

ATSC3s

ATSC4s

ATSC5s

ATSC6s

ATSC7s

ATSC8s

AATSC2c

AATSC3c

AATSC4c

AATSC6c

AATSC7c

AATSC8c

AATSC0m

AATSC1m

AATSC2m

AATSC3m

AATSC4m

AATSC6m

AATSC7m

AATSC1v

AATSC2v

AATSC3v

AATSC6v

AATSC7v

AATSC2e

AATSC4e

AATSC5e

AATSC6e

AATSC8e

AATSC2p

AATSC6p

AATSC7p

AATSC8p

AATSC0i

AATSC2i

AATSC4i

AATSC5i

AATSC6i

AATSC7i

AATSC8i

AATSC2s

AATSC3s

AATSC4s

AATSC6s

AATSC7s

AATSC8s

MATS3c

MATS5c

MATS7c

MATS2v

MATS4v

MATS5v

MATS7v

MATS8v

MATS2e

MATS3e

MATS6e

MATS7e

MATS1p

MATS5p

MATS2i

MATS7i

MATS2s

MATS4s

MATS5s

GATS3e

GATS4s

GATS8s

SpAbs_DzZ

SpMax_DzZ

SpDiam_DzZ

SpAD_DzZ

SpMAD_DzZ

EE_DzZ

SM1_DzZ

VE1_DzZ

VE2_DzZ

VR2_DzZ

SpAbs_Dzm

SpMax_Dzm

SpDiam_Dzm

SpAD_Dzm

SpMAD_Dzm

EE_Dzm

VE1_Dzm

VE3_Dzm

VR1_Dzm

VR2_Dzm

VR3_Dzm

SpAbs_Dzv

SpMax_Dzv

SpDiam_Dzv

SpAD_Dzv

SpMAD_Dzv

EE_Dzv

VE1_Dzv

VR2_Dzv

SpAbs_Dze

SpMax_Dze

SpDiam_Dze

SpAD_Dze

SpMAD_Dze

EE_Dze

SM1_Dze

VE1_Dze

VR1_Dze

SpMax_Dzp

SpDiam_Dzp

SpAD_Dzp

SpMAD_Dzp

EE_Dzp

SM1_Dzp

VE1_Dzp

VR2_Dzp

SpAbs_Dzi

SpMax_Dzi

SpDiam_Dzi

SpAD_Dzi

SpMAD_Dzi

EE_Dzi

SM1_Dzi

VE1_Dzi

VR1_Dzi

SpMax_Dzs

SpDiam_Dzs

SpAD_Dzs

EE_Dzs

VE2_Dzs

VR2_Dzs

nBase

BCUTw-1h

nBonds

nBonds2

nBondsS

nBondsD2

nBondsT

nBondsQ

SpMax1_Bhm

SpMin2_Bhm

SpMax4_Bhv

SpMax5_Bhv

SpMin2_Bhv

SpMin6_Bhv

SpMin7_Bhv

SpMax1_Bhe

SpMax4_Bhe

SpMax6_Bhe

SpMax7_Bhe

SpMin2_Bhe

SpMin3_Bhe

SpMin6_Bhe

SpMin7_Bhe

SpMin8_Bhe

SpMax6_Bhp

SpMax7_Bhp

SpMin2_Bhp

SpMax2_Bhi

SpMax3_Bhi

SpMax4_Bhi

SpMax5_Bhi

SpMin1_Bhi

SpMin4_Bhi

SpMin5_Bhi

SpMax2_Bhs

C1SP1

C2SP1

C3SP3

C4SP3

SCH-3

SCH-4

VCH-3

VCH-4

SPC-4

SPC-5

SPC-6

VPC-4

VPC-5

SP-0

SP-1

SP-2

SP-3

SP-4

SP-5

SP-6

SP-7

VP-3

VP-4

VP-5

VP-6

AVP-1

AVP-2

AVP-3

AVP-4

Sv

Sse

Spe

Sare

Sp

Si

Mv

Mse

Mpe

Mare

Mp

Mi

SpMax_Dt

SpAD_Dt

EE_Dt

VE2_Dt

VR2_Dt

ECCEN

nHBd

nwHBd

nHBint2

nHBint3

nHBint4

nHBint9

nHBint10

nHsOH

nHdNH

nHsSH

nHsNH2

nHssNH

nHaaNH

nHsNH3p

nHssNH2p

nHsssNHp

nHtCH

nHdCH2

nHdsCH

nHCHnX

nHCsatu

nHAvin

nHother

nHmisc

nsLi

nssBe

nssssBem

nsBH2

nssBH

nsssB

nssssBm

nsCH3

ndCH2

ntCH

ndsCH

naaCH

nddC

ntsC

ndssC

naaaC

nssssC

nsNH3p

nsNH2

nssNH2p

ndNH

nssNH

naaNH

ntN

nsssNHp

ndsN

naaN

nsssN

nddsN

naasN

nssssNp

nsOH

nssO

naaO

naOm

nsOm

nsF

nsSiH3

nssSiH2

nsssSiH

nssssSi

nsPH2

nssPH

nsssP

ndsssP

nddsP

nsssssP

nsSH

ndS

nssS

naaS

ndssS

nddssS

nssssssS

nSm

nsCl

nsGeH3

nssGeH2

nsssGeH

nssssGe

nsAsH2

nssAsH

nsssAs

ndsssAs

nddsAs

nsssssAs

nsSeH

ndSe

nssSe

naaSe

ndssSe

nssssssSe

nddssSe

nsBr

nsSnH3

nssSnH2

nsssSnH

nssssSn

nsI

nsPbH3

nssPbH2

nsssPbH

nssssPb

SHBint4

SHBint8

SHBint10

SHsOH

SHdNH

SHsSH

SHsNH2

SHaaNH

SHsNH3p

SHssNH2p

SHsssNHp

SHtCH

SHdCH2

SHdsCH

SHCHnX

SHAvin

SHother

SHmisc

SsLi

SssBe

SssssBem

SsBH2

SssBH

SsssB

SssssBm

SdCH2

StCH

SdsCH

SsssCH

SddC

SaaaC

SssssC

SsNH3p

SsNH2

SssNH2p

SdNH

SaaNH

StN

SsssNHp

SdsN

SaaN

SddsN

SaasN

SssssNp

SsOH

SdO

SssO

SaaO

SaOm

SsOm

SsSiH3

SssSiH2

SsssSiH

SssssSi

SsPH2

SssPH

SsssP

SdsssP

SddsP

SsssssP

SsSH

SdS

SssS

SaaS

SdssS

SddssS

SssssssS

SSm

SsCl

SsGeH3

SssGeH2

SsssGeH

SssssGe

SsAsH2

SssAsH

SsssAs

SdsssAs

SddsAs

SsssssAs

SsSeH

SdSe

SssSe

SaaSe

SdssSe

SssssssSe

SddssSe

SsBr

SsSnH3

SssSnH2

SsssSnH

SssssSn

SsI

SsPbH3

SssPbH2

SsssPbH

SssssPb

minHBd

minwHBd

minHBint4

minHBint10

minHsOH

minHdNH

minHsSH

minHsNH2

minHssNH

minHaaNH

minHsNH3p

minHssNH2p

minHsssNHp

minHtCH

minHdCH2

minHdsCH

minHCHnX

minHAvin

minHother

minHmisc

minsLi

minssBe

minssssBem

minsBH2

minssBH

minsssB

minssssBm

mindCH2

mintCH

mindsCH

minaaCH

minsssCH

minddC

mintsC

minaaaC

minsNH3p

minsNH2

minssNH2p

mindNH

minaaNH

mintN

minsssNHp

mindsN

minaaN

minddsN

minaasN

minssssNp

minsOH

minaaO

minaOm

minsOm

minsF

minsSiH3

minssSiH2

minsssSiH

minssssSi

minsPH2

minssPH

minsssP

mindsssP

minddsP

minsssssP

minsSH

mindS

minssS

minaaS

mindssS

minddssS

minssssssS

minSm

minsCl

minsGeH3

minssGeH2

minsssGeH

minssssGe

minsAsH2

minssAsH

minsssAs

mindsssAs

minddsAs

minsssssAs

minsSeH

mindSe

minssSe

minaaSe

mindssSe

minssssssSe

minddssSe

minsBr

minsSnH3

minssSnH2

minsssSnH

minssssSn

minsI

minsPbH3

minssPbH2

minsssPbH

minssssPb

maxwHBd

maxHBint2

maxHBint4

maxHBint8

maxHBint9

maxHBint10

maxHsOH

maxHdNH

maxHsSH

maxHsNH2

maxHssNH

maxHaaNH

maxHsNH3p

maxHssNH2p

maxHsssNHp

maxHtCH

maxHdCH2

maxHdsCH

maxHaaCH

maxHCHnX

maxHCsatu

maxHAvin

maxHother

maxHmisc

maxsLi

maxssBe

maxssssBem

maxsBH2

maxssBH

maxsssB

maxssssBm

maxdCH2

maxtCH

maxdsCH

maxsssCH

maxddC

maxtsC

maxdssC

maxaaaC

maxssssC

maxsNH3p

maxsNH2

maxssNH2p

maxdNH

maxssNH

maxaaNH

maxtN

maxsssNHp

maxdsN

maxaaN

maxddsN

maxaasN

maxssssNp

maxsOH

maxssO

maxaaO

maxaOm

maxsOm

maxsSiH3

maxssSiH2

maxsssSiH

maxssssSi

maxsPH2

maxssPH

maxsssP

maxdsssP

maxddsP

maxsssssP

maxsSH

maxdS

maxssS

maxaaS

maxdssS

maxddssS

maxssssssS

maxSm

maxsCl

maxsGeH3

maxssGeH2

maxsssGeH

maxssssGe

maxsAsH2

maxssAsH

maxsssAs

maxdsssAs

maxddsAs

maxsssssAs

maxsSeH

maxdSe

maxssSe

maxaaSe

maxdssSe

maxssssssSe

maxddssSe

maxsBr

maxsSnH3

maxssSnH2

maxsssSnH

maxssssSn

maxsI

maxsPbH3

maxssPbH2

maxsssPbH

maxssssPb

sumI

meanI

gmax

MAXDN

MAXDP2

DELS2

ETA_AlphaP

ETA_dAlpha_A

ETA_Epsilon_1

ETA_Epsilon_2

ETA_Epsilon_4

ETA_Epsilon_5

ETA_Psi_1

ETA_dPsi_B

ETA_BetaP

ETA_Beta_s

ETA_BetaP_ns

ETA_dBeta

ETA_BetaP_ns_d

ETA_Eta_R_L

ETA_EtaP_F_L

ETA_EtaP_B

ETA_Eta_B_RC

nHBAcc2

nHBAcc3

nHBDon

nHBDon_Lipinski

IC0

TIC1

TIC3

TIC4

TIC5

SIC1

SIC2

SIC3

SIC4

SIC5

CIC5

BIC0

BIC1

BIC2

BIC3

MIC2

MIC3

MIC4

MIC5

ZMIC5

Kier1

nAtomLAC

McGowan_Volume

MDEC-44

MDEO-22

MDEN-11

MDEN-33

MLFER_BO

MPC2

MPC3

MPC4

MPC5

MPC6

MPC7

MPC8

MPC9

TPC

piPC3

piPC8

piPC9

TpiPC

PetitjeanNumber

nRing

n3Ring

n4Ring

n5Ring

n6Ring

n7Ring

n8Ring

n9Ring

n10Ring

n11Ring

n12Ring

nG12Ring

nFRing

nF4Ring

nF5Ring

nF6Ring

nF7Ring

nF8Ring

nF9Ring

nF10Ring

nF11Ring

nF12Ring

nFG12Ring

nTRing

nT4Ring

nT5Ring

nT6Ring

nT7Ring

nT8Ring

nT9Ring

nT10Ring

nT11Ring

nT12Ring

nTG12Ring

n3HeteroRing

n4HeteroRing

n5HeteroRing

n6HeteroRing

n7HeteroRing

n8HeteroRing

n9HeteroRing

n10HeteroRing

n11HeteroRing

n12HeteroRing

nG12HeteroRing

nFHeteroRing

nF4HeteroRing

nF5HeteroRing

nF6HeteroRing

nF7HeteroRing

nF8HeteroRing

nF9HeteroRing

nF10HeteroRing

nF11HeteroRing

nF12HeteroRing

nFG12HeteroRing

nTHeteroRing

nT4HeteroRing

nT5HeteroRing

nT6HeteroRing

nT7HeteroRing

nT8HeteroRing

nT9HeteroRing

nT10HeteroRing

nT11HeteroRing

nT12HeteroRing

nTG12HeteroRing

RotBFrac

RotBtFrac

LipinskiFailures

GGI1

SpMax_D

SpDiam_D

SpAD_D

EE_D

VE1_D

VR2_D

VABC

VAdjMat

MWC2

MWC3

MWC4

MWC5

MWC6

MWC7

MWC8

MWC9

MWC10

TWC

SRW2

SRW3

SRW4

SRW5

SRW6

SRW8

SRW9

SRW10

TSRW

MW

AMW

WTPT-1

WTPT-5

Zagreb

Total excluded descriptors: 872 DATA SAVED: 13-08-2022 - 01:08:27

Destination file: C:\Users\suraj\Desktop\QSARINS\Excluded datasets

572 descriptors selected for modelling automatically using QSARINS criteria. Default.

Training: 71 Prediction: 29 Variables: 572

Excluded: 0 Missing: 0 Unknown: 0

**Table S13**: Modelling results for all QSARINS models with Var. 1 to 6 along with their statistical validations.


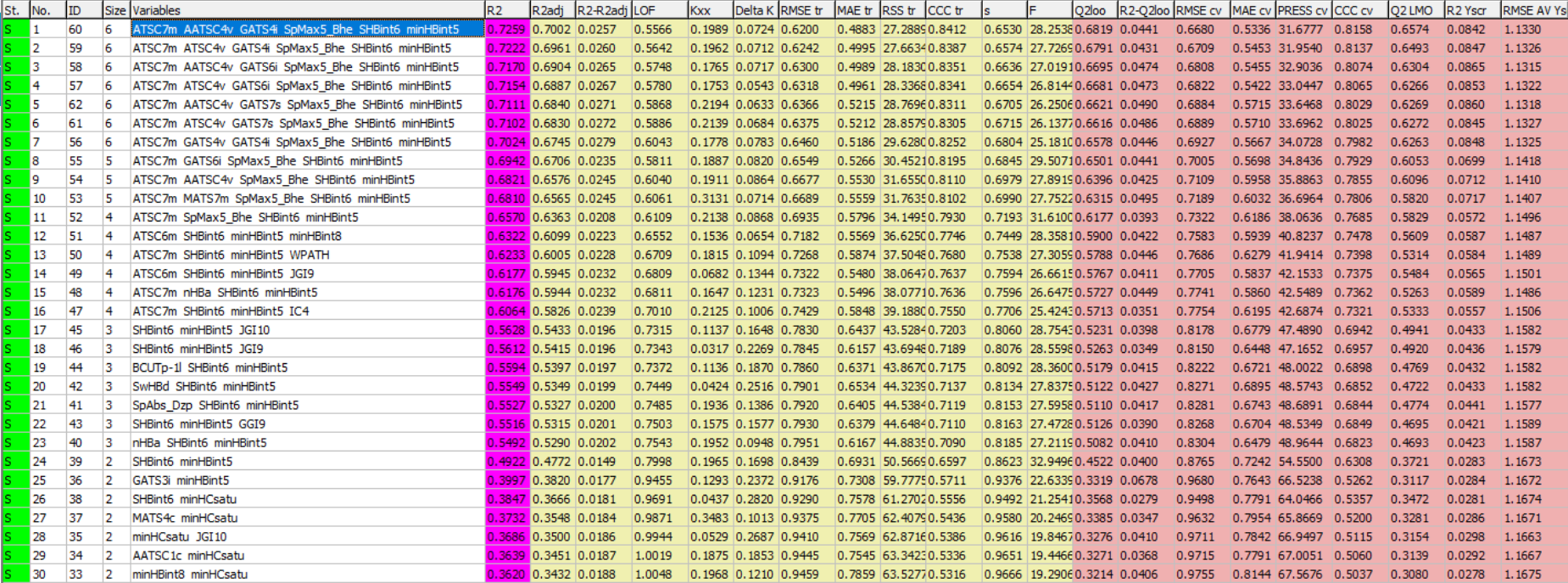


**Table S14**: Correlation matrix for QSARINS model_1_best scored.


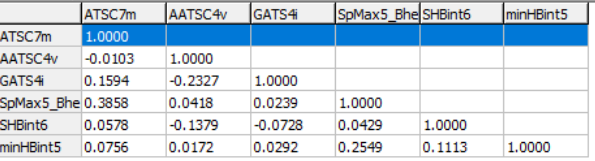


***Validation of docking using binding mode of co-crystallized ligand, Y22, i.e., 6-(trifluoromethyl)-3-{[4-(trifluoromethyl)benzyl]amino}quinoxaline-2-carboxylic acid with target protein PDB ID: 4P8C***

To access the reliability of the molecular docking protocols followed, we re-docked the native, co-crystallized ligand, ***Y22, i.e., 6-(trifluoromethyl)-3-{[4-(trifluoromethyl)benzyl]amino}quinoxaline-2-carboxylic acid*** into an active binding pocket of an enzyme, i.e., M. tuberculosis DprE1 in complex with the non-covalent inhibitor QN127 (PDB: 4p8c). As a result of re-docking, we retained similar binding amino acid residues as reported for Y22 along with the root mean square deviation (RMSD) below 2 Å (Fig.S14).This suggested that the docking protocol followed was accurate.


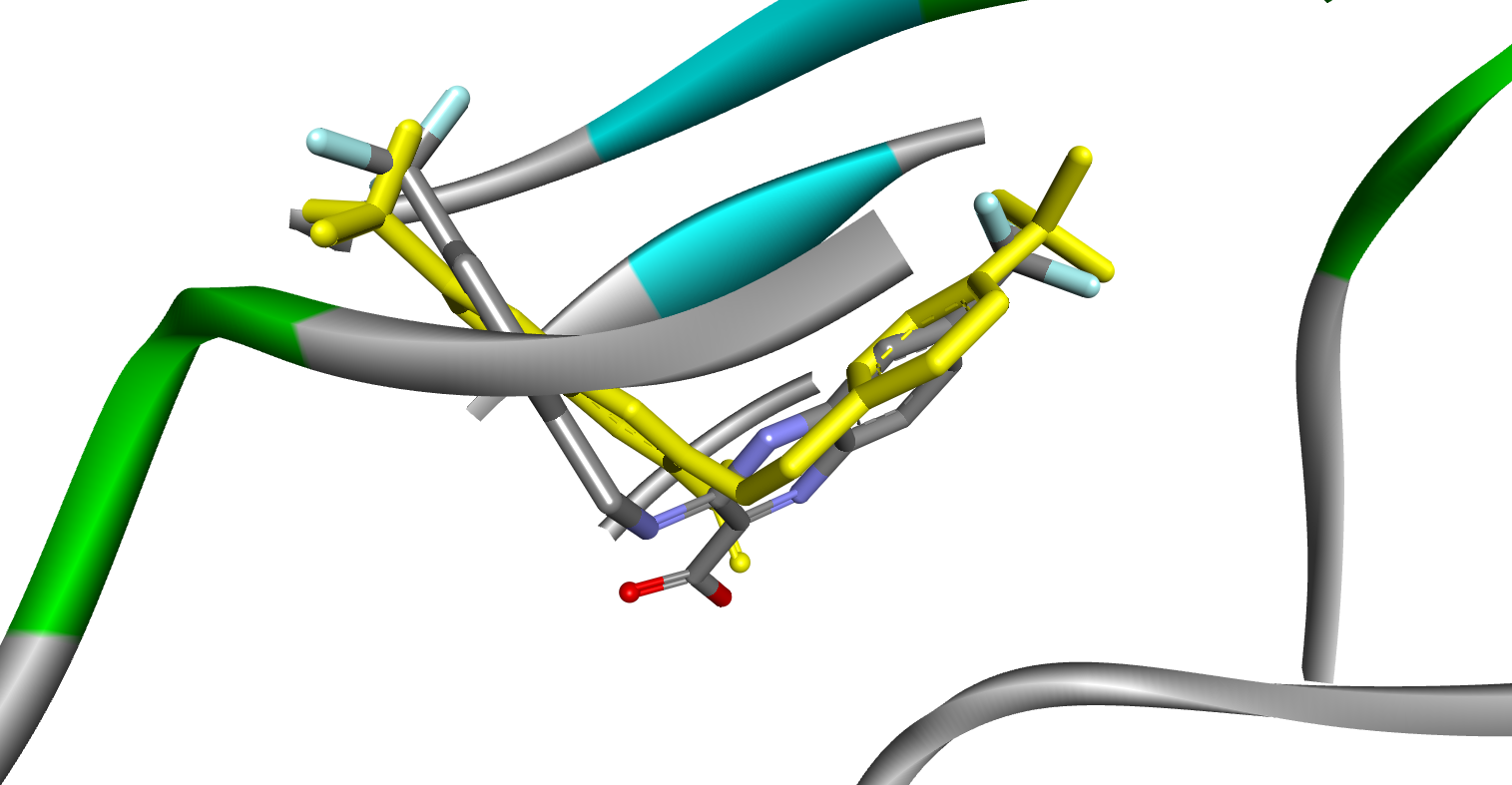


**Figure S14. Re-docking analysis of original** **co-crystalized ligand, Y22, i.e., 6-(trifluoromethyl)-3-{[4-(trifluoromethyl)benzyl]amino}quinoxaline-2-carboxylic acid with target protein PDB ID: 4P8C for docking protocol validations.** [Figure S14 has been visualized with ‘BIOVIA Discovery Studio Visualizer’ V.2022, available at: <https://discover.3ds.com/discovery-studio-visualizer-download>]

**
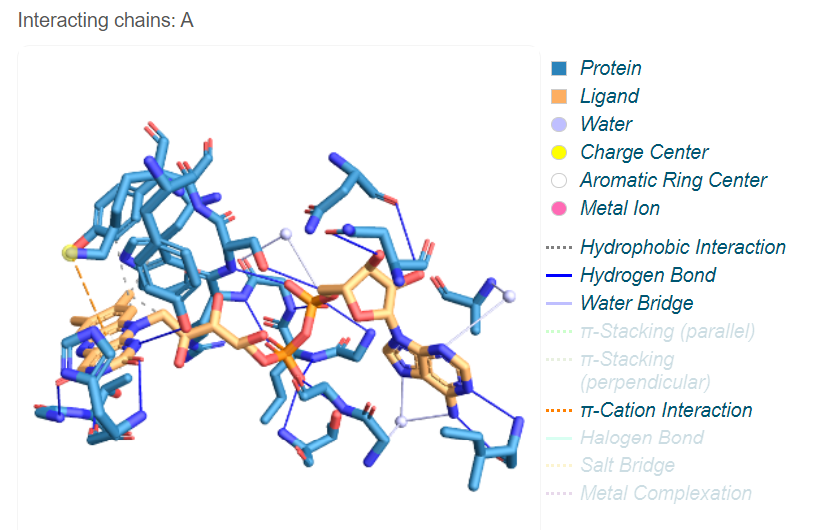
**

**Figure S15. Re-docking analysis using protein-ligand interaction profiler^1^ of original co-crystalized ligand, Y22 found that it has same binding pocket as mentioned in the literature or available in notation in RCSB database.** [Figure S15 has been visualized with ‘the PLIP web tool. V.2022, available at: https://plip-tool.biotec.tu-dresden.de/plip-web/plip/index].

^1^Adasme, M. F. et al. PLIP 2021: expanding the scope of the protein-ligand interaction profiler to DNA and RNA. Nucl. Acids Res. (2 July 2021) 49 (W1): W530-W534. doi: 10.1093/nar/gkab294
